# Design and preliminary analysis of a vaginal inserter for speculum-free cervical cancer screening

**DOI:** 10.1371/journal.pone.0177782

**Published:** 2017-05-31

**Authors:** Mercy Nyamewaa Asiedu, Júlia Agudogo, Marlee S. Krieger, Robert Miros, Rae Jean Proeschold-Bell, John W. Schmitt, Nimmi Ramanujam

**Affiliations:** 1 Department of Biomedical Engineering, Duke University, Durham, North Carolina, United States of America; 2 Duke Global Health Institute, Duke University, Durham, North Carolina, United States of America; 3 Center for Global Women’s Health Technologies, Duke University, Durham, North Carolina, United States of America; 4 3^rd^ Stone Design, San Rafael, California, United States of America; 5 Department of Obstetrics and Gynecology, Duke University, Durham, North Carolina, United States of America; Rudjer Boskovic Institute, CROATIA

## Abstract

**Objective:**

Cervical cancer screening usually requires use of a speculum to provide a clear view of the cervix. The speculum is one potential barrier to screening due to fear of pain, discomfort and embarrassment. The aim of this paper is to present and demonstrate the feasibility of a tampon-sized inserter and the POC*ke*T Colposcope, a miniature pen sized-colposcope, for comfortable, speculum-free and potentially self-colposcopy.

**Study design:**

We explored different designs using 3D computer-aided design (CAD) software and performed mechanical testing simulations on each. Designs were rapid prototyped and tested using a custom vaginal phantom across a range of vaginal pressures and uterine tilts to select an optimal design. Two final designs were tested with fifteen volunteers to assess cervix visualization, comfort and usability compared to the speculum and the optimal design, the curved-tip inserter, was selected for testing in volunteers.

**Results:**

We present a vaginal inserter as an alternative to the standard speculum for use with the POC*ke*T Colposcope. The device has a slim tubular body with a funnel-like curved tip measuring approximately 2.5 cm in diameter. The inserter has a channel through which a 2 megapixel (MP) mini camera with LED illumination fits to enable image capture. Mechanical finite element testing simulations with an applied pressure of 15 cm H_2_O indicated a high factor of safety (90.9) for the inserter. Testing of the device with a custom vaginal phantom, across a range of supine vaginal pressures and uterine tilts (retroverted, anteverted and sideverted), demonstrated image capture with a visual area comparable to the speculum for a normal/axial positioned uteri and significantly better than the speculum for anteverted and sideverted uteri (p<0.00001). Volunteer studies with self-insertion and physician-assisted cervix image capture showed adequate cervix visualization for 83% of patients. In addition, questionnaire responses from volunteers indicated a 92.3% overall preference for the inserter over the speculum and all indicated that the inserter was more comfortable than the speculum. The inserter provides a platform for self-cervical cancer screening and also enables acetic acid/Lugol’s iodine application and insertion of swabs for Pap smear sample collection.

**Conclusion:**

This study demonstrates the feasibility of an inserter and miniature-imaging device for comfortable cervical image capture of women with potential for synergistic HPV and Pap smear sample collection.

## Introduction

Invasive Cervical Cancer (ICC) is the second most common female cancer in low and middle-income countries (LMICs) and the seventh most common in high-income countries [1]. Annually, over 500,000 women are diagnosed, causing over 270,000 deaths recorded with more than 75% of cases occurring in Africa and India [1]. The World Health Organization (WHO) estimates that currently 88% of worldwide ICC mortalities occur in LMICs [2], and this rate is expected to increase to 98% by 2030, furthering the disparities [3] as the total number of annual worldwide mortalities increases to nearly 400,000 [4]. Though early diagnosis and treatment of cervical pre-cancers have been shown to significantly increase survival rates [1, 5], diagnostic tools are not widely available in LMICs. Currently, as an alternative to cytology screening, the standard-of-care screening method in most LMICs is visual inspection with acetic acid (VIA), with or without digital image capture. This method involves the use of a speculum to expand the vaginal canal to enable a clear field-of-view of the cervix, for visualization with a colposcope, camera or directly by the health provider (naked eye). Speculums are necessary mainly because of the need to expand the entire vaginal canal. During the VIA procedure, 3–5% acetic acid is applied to the surface of the cervix. A positive VIA exam shows a sharp, distinct, well-defined, dense aceto-white area, with or without raised margins [6]. If a camera or digital colposcope is available, images of the cervix can be visualized at higher magnification and can also be archived for further analysis and review.

The speculum has been identified as a significant factor in the resistance of women to undergo cervical cancer screening, largely due to anxiety, fear, discomfort, pain, embarrassment, and/or vulnerability during the procedure. A study of 354 women in Moshi, Tanzania revealed that key factors for cervical cancer screening were significant concerns about embarrassment and pain due to screening from the speculum as well as physician gender [7]. In Australia, a study seeking to determine women’s attitudes towards physician versus self–insertion of the standard speculum found that 91% of 133 women would choose self-insertion over physician insertion, and that women have indicated discomfort, embarrassment and vulnerability from having another person insert a device and examine their cervix [8]. In the U.S., even though there is greater access to health care, compliance rates to cervical cancer screening vary, and embarrassment and fear of pain during examination have been reported as potential barriers to screening [7, 9–11]. The speculum is a cause of discomfort, especially for women with vaginismus, a condition involving the involuntary tightening of the vagina often caused by sexual abuse [12]. East African countries, such as Tanzania, have among the highest sexual violence rates worldwide [13, 14] and also have the highest rate of cervical cancer incidence and mortality [15]. Thus, it is these women who are in greatest need for frequent cervical screening while simultaneously requiring a less painful and invasive screening method without the use of a speculum.

The speculum has been in existence in various shapes and forms since the tenth century and has evolved with hundreds of modifications in attempts to enhance exposure. J. Marion Sims, the “father of gynecology,” developed the first rudimentary prototype of the modern speculum out of a bent spoon [16]. The semblance to the standard bivalve speculum was put forward by the manufacturer Charriere who introduced the bivalve, tri-blade and four-blade speculum [16]. This inspired the duck bill designs of the familiar Cusco speculum in 1870 and the Graves speculum in 1878 [16]. These are cold, hard, metal devices with two bills each that expand the entire vaginal canal. Since the introduction of duck billed speculums, there have been few improvements to make them more comfortable and acceptable for women. Slight changes in design have involved introducing a variety of sizes and making the speculum out of plastic, etc. Current speculums are designed for an external user, which makes it difficult for self-insertion by women. Being able to self-insert is important in being able to re-adjust. When there is discomfort. Furthermore, in cases where women have tilted uteri or lax vaginal walls due to having a larger body size or a high parity, increased manipulation or use of an extra device, such as a side wall retractor, is needed to obtain a clear view of the cervix [17]. This further adds to discomfort and pain during vaginal examinations.

The few attempts at major changes in the redesign of the speculum have been unsuccessful. The FemSpec, a clear plastic cylinder with inflatable air pockets, was developed in 2005 by FemSuite, San Francisco [18]. The FemSpec has a tampon-sized insertion diameter and, once inserted, can be inflated to expand the vaginal walls. This was taken off the market due to the reluctance of medical professionals to embrace the device [18]. We acquired this device and found that it had sharp plastic edges and was unable to withstand high vaginal pressures. The Vedascope, designed in Australia, is an encompassing speculum/colposcopic device, which dilates the vagina with air inflow and is attached to a camera and illumination for colposcopy. Though 92% of women in the study indicated a preference for the Vedascope to the speculum, it is very bulky, expensive and requires physician placement. Additionally, it has a potential risk for air embolism, which can be fatal [19, 20].

The difficulty in redesigning the speculum has been primarily due to limitations in the external visualization of the cervix. The speculum must provide a clear view of the cervix from outside the patient’s body through the vaginal canal, which ranges from about 4–9.5 cm in length [21]. This implies that the patient’s vaginal walls must be out of the view of the visual method. Our group has addressed this challenge by developing a pen-sized trans-vaginal colposcopy device, the Point-of-care tampon (POC*ke*T) Colposcope, which uses a 5 megapixel (MP), complimentary metal-oxide-semiconductor (CMOS) camera and light emitting diode (LED) illumination. The addition of light plus a camera allows for an image of the cervix to be lit and captured, thereby obviating the need to part the vaginal walls to view the cervix. The POC*ke*T Colposcope has been proposed as a portable, low-cost colposcope for screening in low-resource settings, and can be inserted into the vagina for close-up cervical image capture in a standard speculum-based inspection [22]. A distinct advantage of this device is that it can serve as a surrogate eye and allow for a more patient-friendly alternative to the standard duck billed speculum, which would enable comfortable, speculum-free and eventually self-screening including cervical image capture by patients, either in their homes or in clinics with minimal physician/nurse guidance.

**Fig 1:**
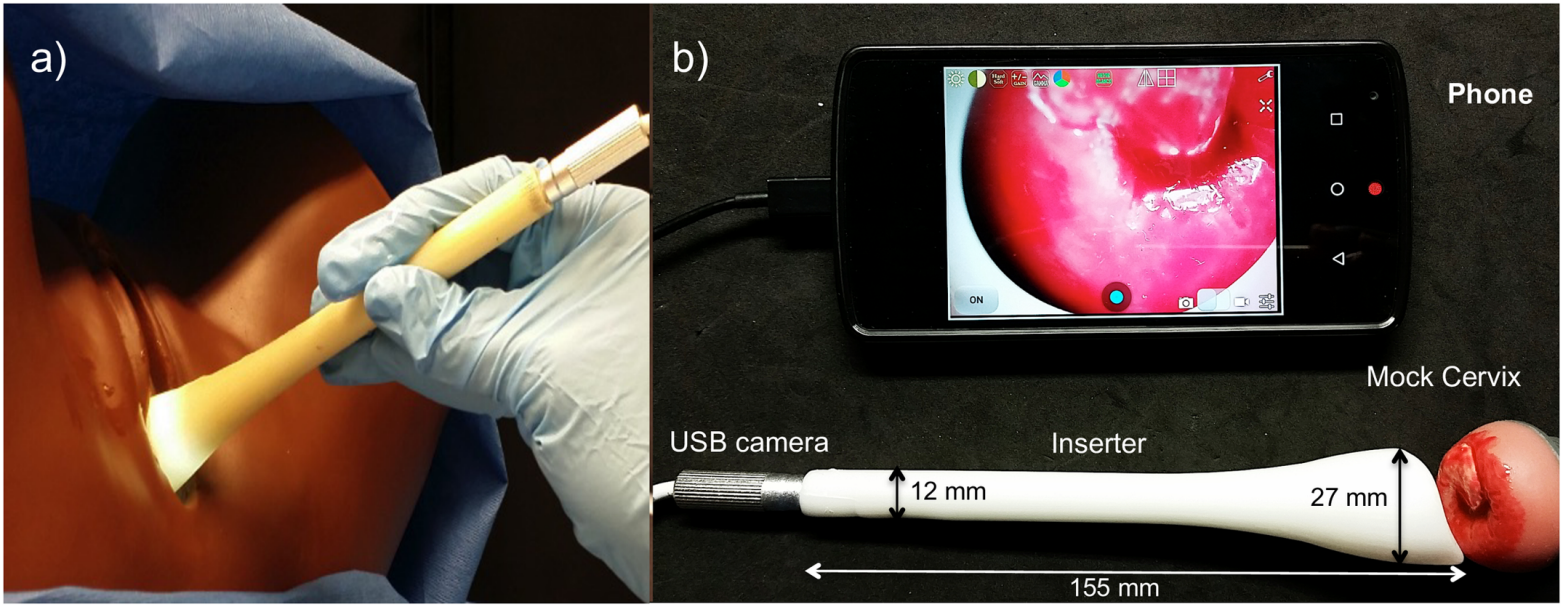
(a) Vaginal Inserter with digital colposcope inserted, imaging a mannequin cervix with white LED illumination. (b) Image of the device with dimensions with the mini USB camera inserted and interfacing with the smartphone for imaging. The image shows the camera in the inserter device imaging a mock cervix in real time.

We have developed a patient-centered vaginal inserter as an alternative to the speculum (Fig 1a), for use with our POC*ke*T Colposcope to enable speculum-free, self-insertion, and image capture of the cervix. The device has a slim tubular body of 1.0 cm diameter opening up to a curved funnel-like tip measuring approximately 2.5 cm in diameter. The curved tip enables easy manipulation of the cervix, especially in cases where the patient has a tilted uterus. The inserter has a channel through which a 2MP mini universal serial bus (USB) camera with LED illumination fits to enable cervix image capture (Fig 1b). The channel also enables acetic acid/Lugol’s iodine application and insertion of swabs for Pap smear sample collection. The camera can be connected to any USB enabling device such as a mobile phone, tablet or computer for image capture. This paper presents the computational design, fabrication, bench testing and evaluation of the device with 15 volunteers. Our results demonstrate that the device can be combined with our POC*ke*T Colposcope to enable speculum-free cervix visualization. This inserter not only has the potential to allow for patient-centered colposcopy, but can also be used to center and identify the cervix for physician-based or self-Pap/HPV testing.

## Materials and methods

### Modeling and finite element analysis of candidate inserter designs

**Fig 2:**
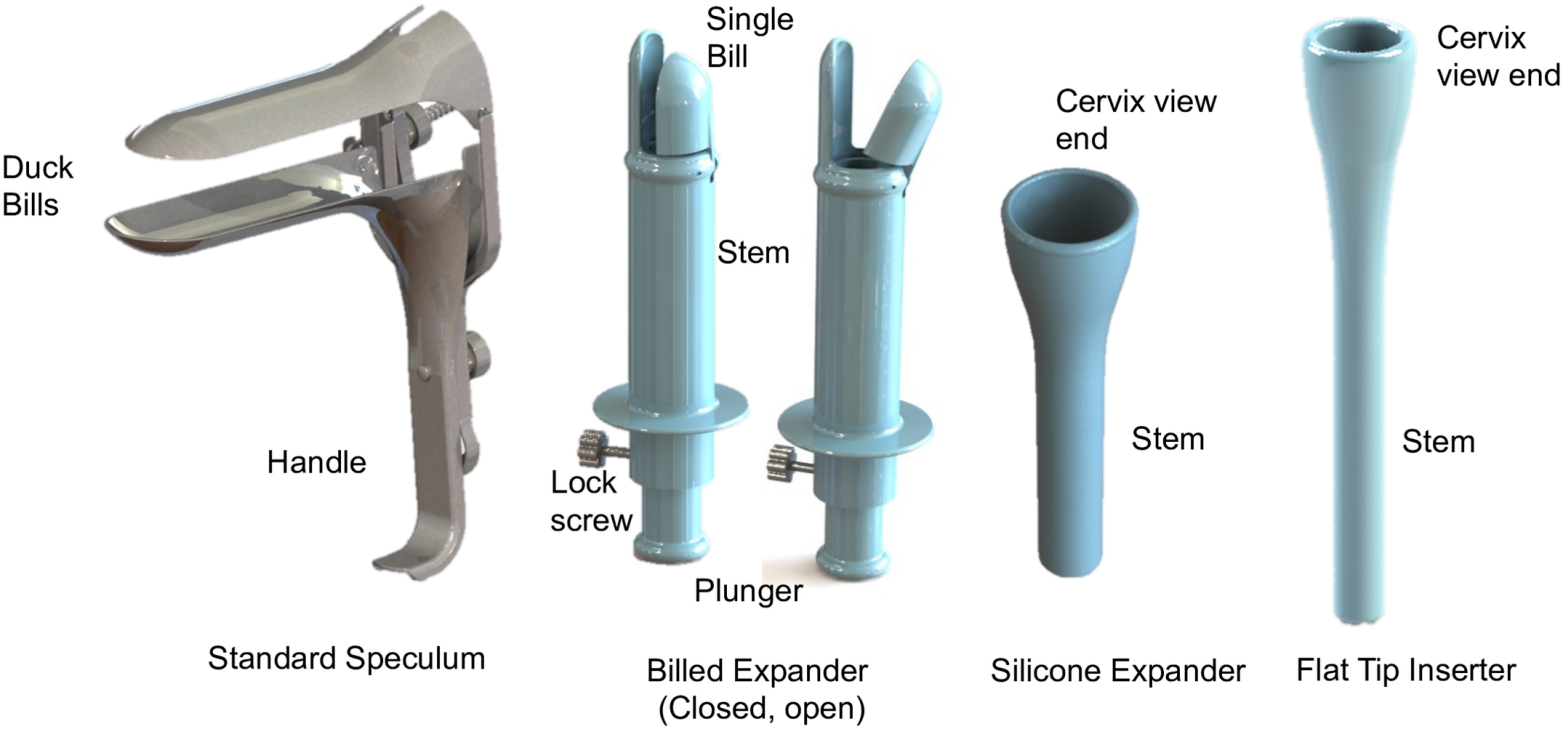
CAD designs of standard speculum (from GrabCAD) and vaginal inserter/expander prototypes.

Several inserter designs were proposed as potential replacements for the speculum to enable speculum-free self-colposcopy. Three main designs were explored: a mechanical billed expander, a silicone expander and a probe inserter (Fig 2). The idea for the billed expander was to have a mini speculum at the tip of a probe, which would be closed upon insertion and opened once inserted to expand approximately an inch of the vaginal walls closest to the cervix. Since there would only be minimal expansion of the walls, we speculated that this would be more comfortable than the speculum, which expands the entire vaginal canal for cervix visualization. We also considered a silicone expander, with similar expansion material and geometry to a menstrual cup. The head is connected to a hollow cylindrical stem, which serves as the working channel for camera insertion, acetic acid application, etc. The idea for this model was to insert the silicone component similar to how a menstrual cup is inserted (by folding over the silicone). Once inserted, the silicone cup would expand and the stem could be used to further push up the cup until it reaches the cervix. When a menstrual cup is inserted properly, it cannot be felt at all hence we anticipated that this approach would be a comfortable alternative to the speculum. The third model was based on a vaginal suppository design, enabling simple insertion and removal. It is comprised of a single hollow probe with a funneled out tip of a smaller open diameter than the silicone cup (~2.5 cm). The probe with the camera can be inserted into the vaginal canal and pushed through until the cervix comes into view. Design specifications and constraints (Table 1) were considered in the design of each model. Other important considerations included ease and simplicity of use by both physician and patients.

**Table 1:** Design specifications and constraints.

Specification/Constraints	Metric	Rationale
Visual Area	2.0cm– 3.0 cm	Average cervix diameter = 2.5cm [23]
Working distance from camera to cervix	4.5cm– 7.0cm	Imaging distance determined (specific to camera) which enables visual area specified to be achieved
Minimum working channel diameter	0.8 cm	Smallest diameter of cameras in use (Supereyes Y002)
Total length	15cm	Maximum vaginal length (10 cm) plus grip (5 cm) [21]
Range of vaginal pressures to withstand	0.1–12 cm H_2_O	Range of vaginal pressure for supine position [24]
Safety factor	4	Medical device factor of safety [25]
Biocompatibility	ISO 10993–5, 10993–10	Biological evaluation of medical device tests for irritation, sensitization and cytotoxicity [26, 27]
Sterilization compatibility	ETOH, Autoclave, sodium hypochlorite	Sterilization recommendations for medical equipment [28, 29]
Compatibility with other devices	USB Camera, Cotton swabs, cytobrush	Tools that would be used with the inserter for cervical cancer screening

The designs were modeled using 3D computer-aided design (CAD) software, SolidWorks^®^. The 3D model for the speculum was obtained from GrabCAD. The mechanical expansion design was comprised of four main parts: 1) an outer stem with an extension on one end (fixed bill) for manipulating the cervix; 2) a single bill with an extender connected to a plunger to expand and close the bill; 3) the silicone cup model comprised of a shore 40A menstrual silicone cup attached to a plastic hollow cylinder; and 4) the probe comprised of a single plastic piece with a hollow cylinder which tapers open at one end.

The different designs were tested using finite element static analysis by applying a pressure of 15 cm H_2_O, slightly higher than the vaginal pressures of a woman is in supine position [24] (protocol: http://dx.doi.org/10.17504/protocols.io.hrgb53w). Analysis was simplified by assuming the devices had been inserted and expanded (where applicable) to a fixed position and fixed boundary conditions were applied. Designs were tested using stainless steel (A316) for the speculum, ABS plastic for billed mechanical expansion and probe inserter designs and silicone (Shore 40 A durometer) for the silicone cup design. Stainless steel was selected based on prior materials used for speculum design. ABS plastic was selected based on rapid prototyping materials approved for medical use and the silicone material was selected based on existing materials used for menstrual cups. The Factor of Safety (FOS), a dimensionless mechanical term describing the capacity of a device to withstand loads greater than the expected load, was determined by dividing the yield strength by the maximum strength. The food and drug administration (FDA) does not specify any particular FOS for medical devices. For our context, we selected a factor of safety of 4 which has been previously used in the design of a speculum by a different group [25].

### Rapid prototyping and bench testing

Once the designs were finalized, each device was rapid prototyped using a 3D printer (dimension 1200es, Stratasys, Ltd.). The mechanical expansion device was assembled using stainless steel pins. The silicone device was assembled by attaching a menstrual cup to a 3D printed cylindrical stem.

**Fig 3:**
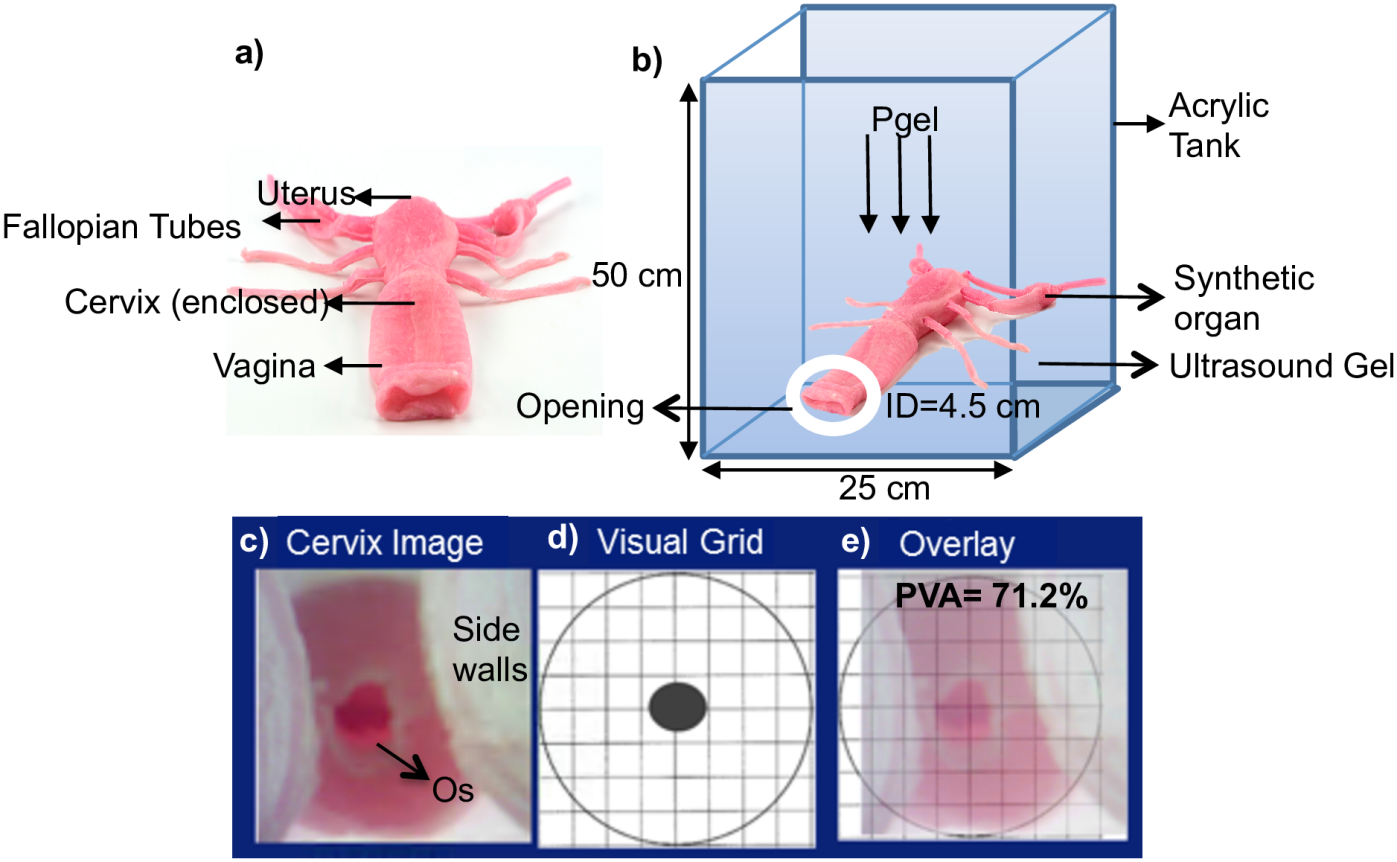
a) Vaginal organ model b) Schematic of phantom with vaginal organ suspended in acrylic tank and filled with gel. Pgel is pressure from the gel, ID is inner diameter of the opening. c) Cervix image from phantom showing central os and side walls d) Circular cervix grid for calculating percent visual area e) Cervix overlaid with circular grid with PVA calculated.

For bench testing, we created a custom-made vaginal phantom using a synthetic female reproductive organ obtained from Syndaver labs^™^. The organ was comprised of outer genitalia, labia, a vagina, cervix, inner and outer os (cervical opening into the uterus), uterus, ovaries and fallopian tubes. The structural design was based on an amalgam of CT and MRI images from actual patients and the synthetic tissues employed in construction had been validated against the mechanical, physicochemical, thermal and dielectric properties of living tissue (Syndaver labs^™^). The organ was compatible with both imaging and surgical equipment and devices, hence providing a realistic experimental testing platform for our prototypes. Different vaginal pressures were simulated by suspending the organ in a custom-made tank that was filled with ultrasound gel of known density, to appropriate heights (Fig 3a), to provide the desired pressures through the relation shown in Eq 1:

Pressure (P) = density (ρ) x acceleration due to gravity (g) x height (h) (1)

Where the density **(ρ)** was equal to 1 g/cm^3^ and acceleration due to gravity **(g)** was equal to 10 m/s^2^. Each prototype was inserted into the phantom vagina, opened to the desired position (where applicable) and images of the cervix were captured with a 2MP USB camera (Supereyes Y002) and saved for further analysis to determine the percent visual area (PVA) that each prototype allowed. It should be noted that the 2MP USB camera used for our experiments and clinical studies is a low-cost, off-the-shelf camera with no added features and is different from the 5MP POCkeT Colposcope camera on which our group previously published [22]. However, all of the proposed designs are adaptable to the 5 MP POCkeT colposcope by simply increasing the diameter of the stem diameter of the speculum-free device. Testing was performed under vaginal pressures of 0.1cm H_2_O to 15 cm H_2_O, spanning the range of supine position pressures previously cited (protocol: http://dx.doi.org/10.17504/protocols.io.hrhb536). Images were also captured with the standard Graves speculums for comparison. The percentage of unobstructed cervical area, with the os centered, was calculated from each image using a circular grid [30] and Eq 2, which provided a standardized comparison regardless of distance between the camera and the cervix.

$$PVA=\;\frac{\#\;squares\;within\;cervix\;region}{total\;no.\;of\;squares}\;x\;100$$ (2)

**Fig 4:**
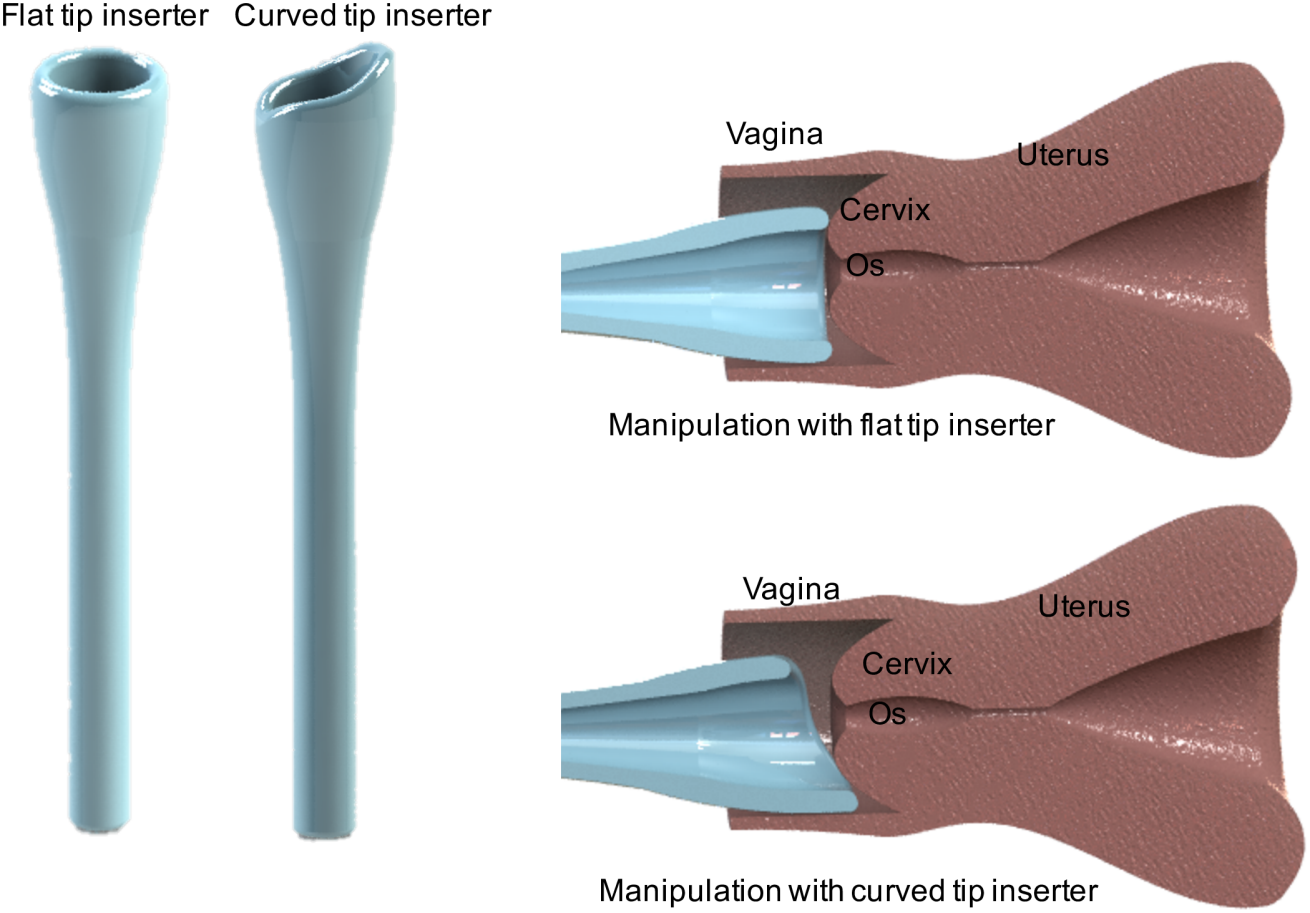
Original flat tip inserter and optimized curved tip probe inserter. Images of the devices inserter into the vagina show how the curved tip of the optimized inserter enables manipulation of the cervix.

Based on initial favorable testing with the flat tip inserter, we designed an iteration of it for further testing to enable manipulation of the cervix for effective use with women (see Results, Phantom Testing). The probe inserter was further iterated on to enable manipulation of the cervix for effective use in women with severely tilted uteri, namely retroverted (tilted posteriorly), anteverted (tilted anteriorly) and sideverted uteri (tilted to the side) which condition affects about 20% of women [31]. The main modification made to the probe inserter was the addition of a slanted curvature to the tip as shown in Fig 4. Since the cervix is shaped like a semi-sphere with the curved portion interfacing with the inserter, protrusion of the tip of the inserter is required to easily manipulate it (Fig 4). The variation of the design was tested in the phantom to assess ability to manipulate the cervix at different tilts.

To test the two probe inserters, the uterus in the phantom was tilted under constant pressure (5 cm H_2_O) 30° to the side for sideverted, 30° downwards for retroverted and 30° upwards for anteverted position. Height and angle measurements were achieved using a 30 cm ruler and a protractor attached to the acrylic walls of the phantom respectively. The prototypes were inserted and used to gently manipulate the cervix into the mid-position, with the os centered as well as possible. Images were then taken for further visual analysis to estimate how much of the cervix, with the os centered, could be visualized. The percentage of unobstructed visual area was calculated using Eq 2. The offset of the os from the center for each image was also determined by measuring the distance from the position of the os to the center of the grid. Each experiment was repeated 5 times and all results were statistically compared between designs and the standard speculum using Student t-tests (α = 0.05).

### Volunteer study

A pilot exploratory study in human subjects was conducted to demonstrate feasibility of the Inserter with a 2MP camera to capture cervix images without the speculum while providing higher level of comfort compared to the speculum. This study was approved by Duke University’s institutional review board (IRB) and performed with approved protocol, informed consent process, and data storage system (Pro00008173) at the Duke University Medical Center (DUMC). Fifteen healthy volunteers (21–65 years of age) provided written informed consent using an institutional IRB approved consent form for the insertion and image capture procedure. Since Pap smears are recommended for women starting 21 years of age and some of these women may present with an abnormal Pap and need a colposcopy, we considered women 21–65 years of age for our study [32]. Participating volunteers were also provided a copy of their signed consent form. The original consent form is kept in a secured research binder that is also used to document the consent process and procedure followed the approved protocol in a locked cabinet residing in a locked office that only IRB approved personnel on the study have access to. After informed consent was obtained, volunteers were asked to complete a pre-examination questionnaire to assess their experiences with and attitudes towards the standard speculum and vaginal examinations. Volunteers were then trained for about 5 minutes on how to use the inserter with the camera and capture images of their cervices using a pelvic mannequin. Once trained, each volunteer inserted the device herself and maneuvered it to find her cervix with the aid of the camera (for image capture) and mobile phone (for image display). The physician on the study (Schmitt, JW), assisted when the volunteer found it difficult to find her cervix. Physician assistance came in the form of guidance on how to manipulate the inserter rather than physical manipulation of the inserter. Images were captured once the cervix was in view. Since these were healthy volunteers, no acetic acid was applied during the procedure. Between patients, we performed high level disinfection by submersion in 2% hydrogen peroxide for eight minutes under room temperature, as recommended for semi critical devices (contact with mucous membranes or nonintact skin during patient use) [33, 34]. After the examination was complete, the volunteers were asked to complete post-examination questionnaires. The volunteer post-examination questionnaires assessed comfort and compared their experience with the inserter to previous examinations with a speculum. Volunteers who had no previous speculum examinations did not complete the portion of the questionnaire that asked for comparisons between previous examinations with the inserter examination. In addition, responses from one volunteer were eliminated due to failure of the volunteer to complete required sections of the questionnaires. For the study we qualitatively compared the visualization of the cervix and the centeredness of the os for both the flat tip and curved tip inserters.

## Results

### Computational mechanical testing

**Fig 5:**
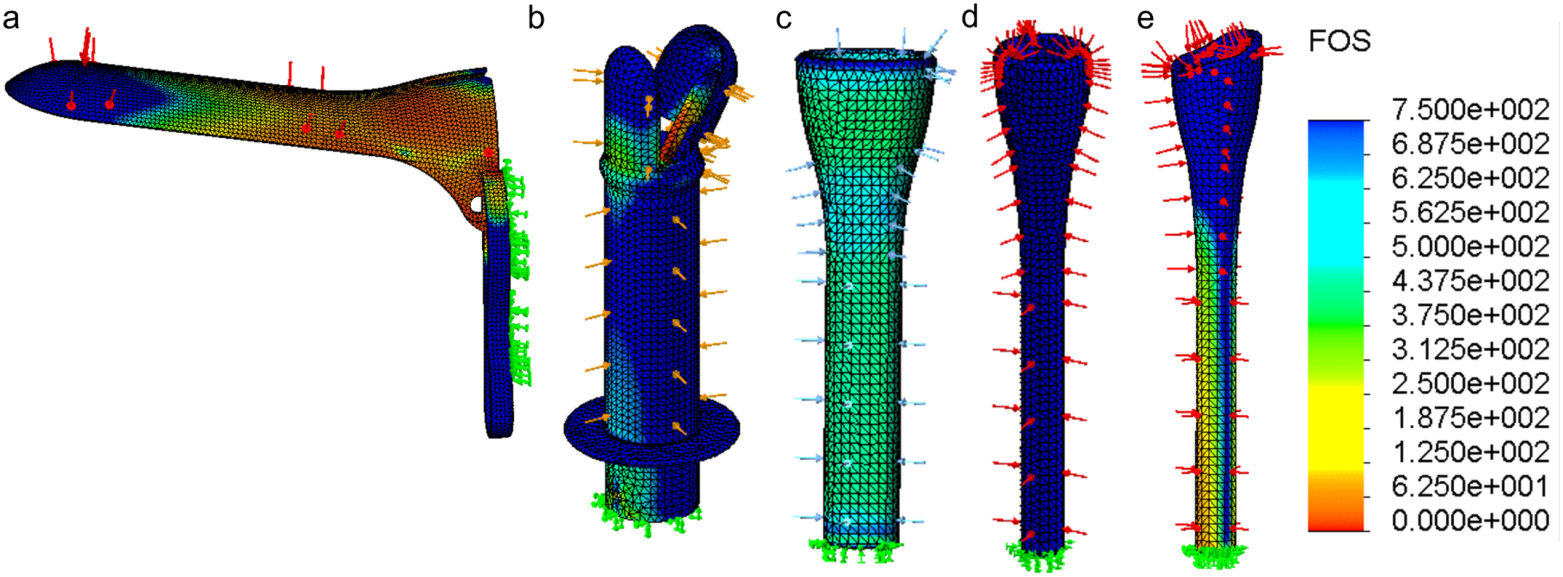
Finite element analysis and factor of safety (FOS) plots for the standard speculum and the inserter/expander prototypes. Green arrows indicate fixed surfaces and other colored arrows indicate applied pressure. All the prototypes designed have minimum FOS greater than 4 with the minimum factor of safety for the devices as follows: a) speculum = 8.07, b) billed expander = 11.67, c) silicone expander = 346, d) flat tip inserter = 3095 and e) the curved tip inserter = 90.9.

The finite element analysis showed factor of safety (FOS) plots over the entire device, ranging from relatively low values in red to high FOS values in blue (Fig 5). The lower the FOS, the more likely the device is to fail under pressure and possibly fracture within the patient’s body during use. This metric is especially important for devices that have to expand to open the vaginal walls. All the devices had a minimum FOS above the acceptable value of 4. The standard speculum had a minimum FOS of 8.07. The billed expander was rated 11.67; the silicone expander, 346; the flat tip inserter, 3095.3; and the curved tip inserter, 90.9.

### Phantom testing

**Fig 6:**
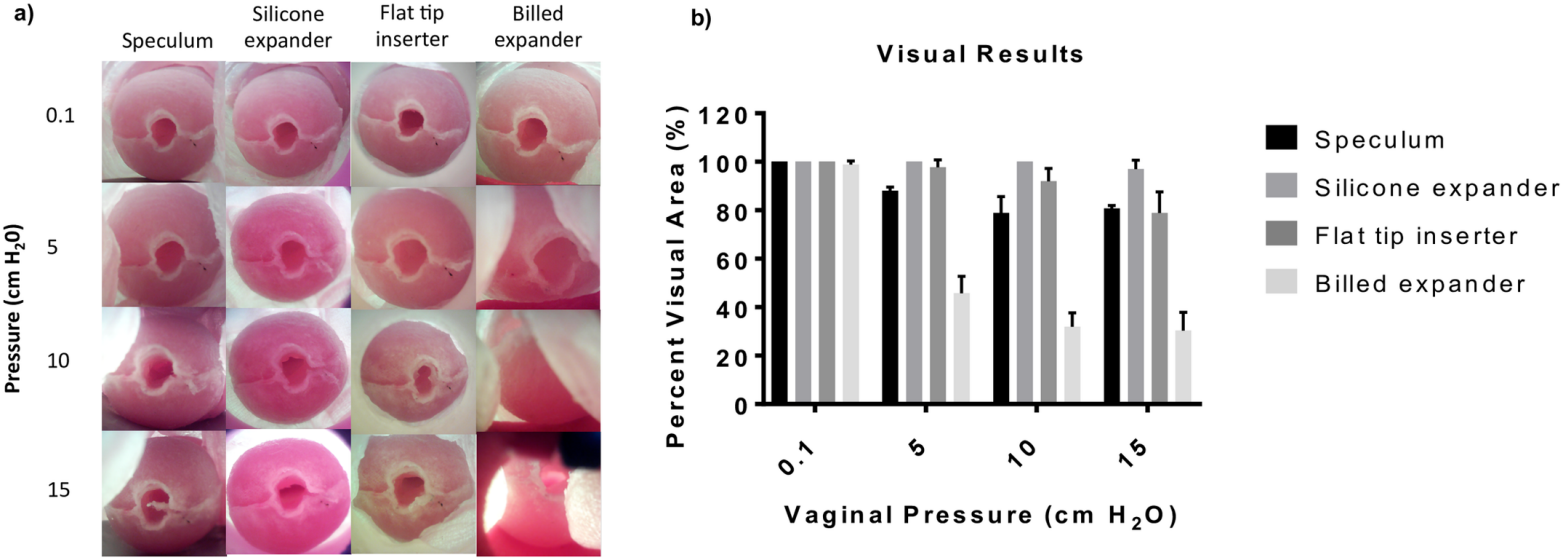
a) Results from experimental determination of percent visual area showing images of the mock cervix (with centered os) in the vaginal phantom with the graves speculum, silicone expander, flat tip inserter and the billed expander. Images are captured at different vaginal pressures 0.1, 5, 10 and 15 cm H_2_O. b) Grouped bar plot of mean percent visual area of the cervix for the different devices under the different pressures. Error bars are standard deviations.

Testing in the phantom was conducted to compare visualization of the cervix afforded by the different prototypes under different pressures (0.1–15 cm H_2_O) corresponding to and slightly above a range of vaginal pressures of 0.1–12.0 cm H_2_O measured in the supine position [24]. Images were also taken with a standard medium-sized Graves speculum to compare visualization with the standard of care. Fig 6a and 6b show representative images and a graph of the percent visual area from each prototype as well as the standard Graves speculum of the cervix phantom at an untilted position under different pressures. The images show the central os of the cervix, the cervix and the vaginal walls in some cases. Table 2 shows the mean percent visualization for the speculum, silicone expander, flat tip inserter and the billed expander.

**Table 2:** Mean percent visual area (PVA) enabled by speculum and different inserter designs under a range of supine position vaginal pressures and normal (axially) positioned uteri.

Pressure value (cm H2O)	0.1	5	10	15
**Speculum mean PVA (%)**	100 +/- 0	88.1+/-1.5	78.8 +/- 6.8	80.7 +/- 1.2
**Silicone expander mean PVA (%)**	100 +/- 0	100 +/- 0	100 +/- 0	96.9 +/- 3.8
**Flat tip inserter mean PVA (%)**	100 +/- 0	97.7 +/- 3.1	92.0 +/- 5.3	78.86 +/- 8.7
**Billed expander mean PVA (%)**	98.86 +/- 1.5	45.78 +/- 7.0	31.94 +/-5.7	30.38 +/- 7.4

Although the devices performed similarly at 0.1 cm H_2_O, we found statistically significant differences at other pressures. Statistical analysis showed that the mean PVA for the silicone expander was statistically higher (i.e. better) than the standard speculum (p<0.001) for 5, 10 and 15 cm H_2_O. The PVA for the flat tip inserter was also statistically higher than the standard speculum (p<0.01) for 5 and 10 cm H_2_O. The billed expander was worse than the speculum (p<0.00001) for 5, 10 and 15 cm H_2_O. Even though the silicone expander provided the best PVA, it was limited by the complexity of insertion and removal. Because the flat tip inserter also performed better than the speculum, we decided to further optimize this design by adding a curved tip for manipulation of the cervix and further experimental and clinical studies.

**Fig 7:**
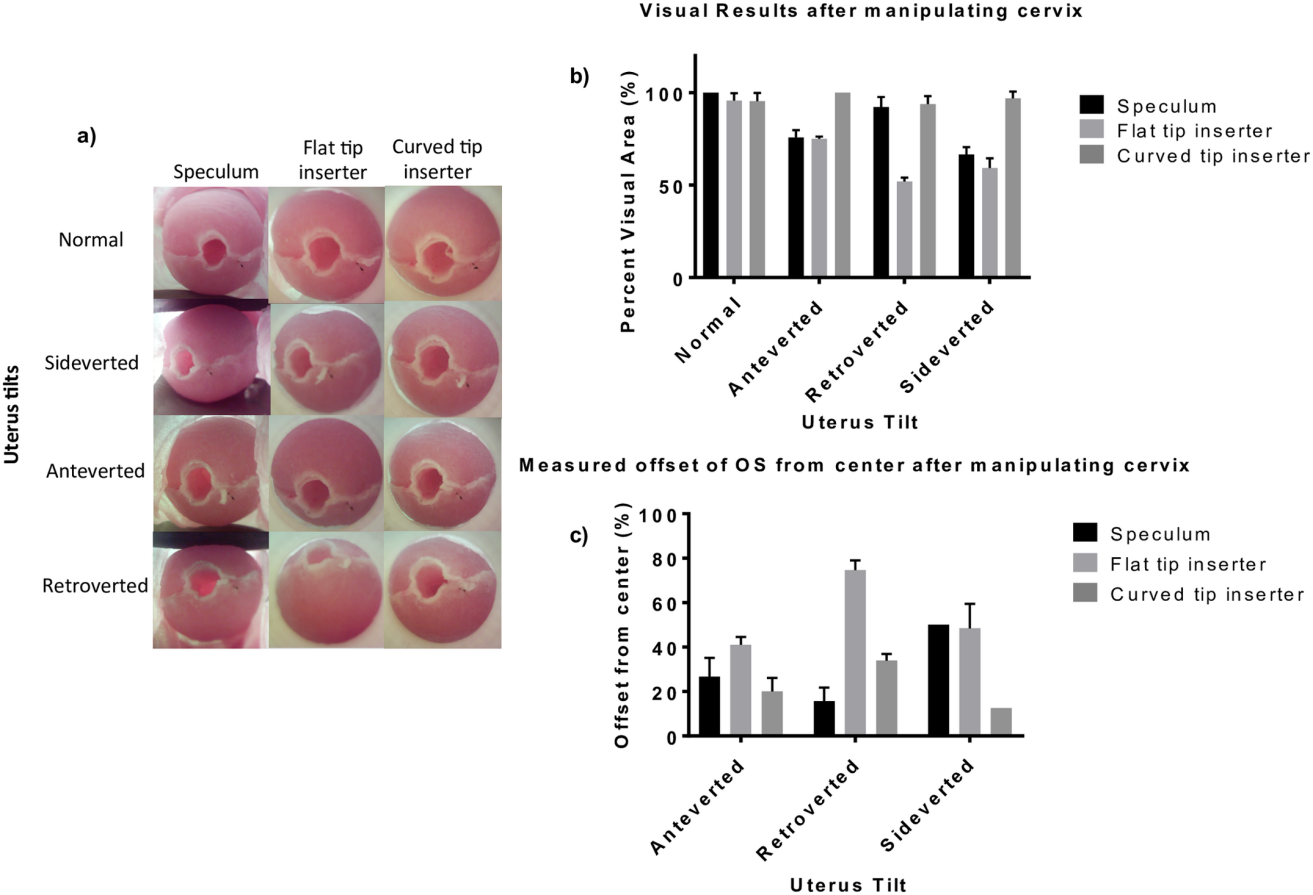
Results from comparing the ability of the speculum, flat tip and curved tip probe inserters to manipulate the cervix. a) Images of the cervix at after manipulation with the standard speculum, flat tip and curved tip inserters for normal, sideverted, anteverted and retroverted positions. b) Top: Measured mean offset of the os from the center for each device under different uterus tilts. Bottom: Mean PVA for each device under different uterus tilts. Error bars are standard deviations.

The two variations of the inserter were tested on the vaginal phantom, which had been positioned at different tilts to simulate cervices that are sideverted, anteverted and retroverted. Fig 7a shows images from testing, after the tilted cervices had been manipulated towards the center position by the Graves speculum and both the flat tip and curved tip inserters. Fig 7b and 7c show graphs of the percent visualization and percent offset of the os from the center, respectively.

Table 3 shows the mean percent visual area (PVA) of the cervix enabled by the speculum, flat tip and curved tip inserters after attempts by each device to center the tilted uterus under constant pressure. Table 4 shows the mean percent offset of the os from the center after manipulating the cervix. Results showed that the curved tip was best able to manipulate the cervix to a position with the os closest to the center, providing the lowest offsets with p <0.00001 for the sideverted position compared to the speculum and p< 0.001 for all three positions compared to the flat tip inserter. The curved tip also provided the highest PVA across the anterverted and sideverted positions compared to the speculum (p <0.00001) and across all three positions compared to the flat tip inserter (p<0.0001).

**Table 3:** Mean percent visual area (PVA) enabled by speculum, flat tip and curved tip inserter designs under 500 cm H_2_O pressure and various uterine positions (normal, anteverted, retroverted and sideverted).

Uterine Position	Normal Position	Anteverted	Retroverted	Sideverted
**Speculum mean PVA (%)**	100 +/- 0	75.8 +/-4	92.0 +/- 5.3	66.5 +/-4
**Flat tip inserter mean PVA (%)**	95.78 +/- 3.92	75+/-1.2	51.9 +/- 2.1	59.2 +/- 5.4
**Curved tip inserter mean PVA (%)**	95.4 +/-4.5	100 +/-0	93.8 +/-4.3	96.9 +/- 3.8

**Table 4:** Mean percent offset of os from central position after manipulation with the speculum, flat tip and curved tip inserter designs under 500 cm H_2_O pressure and uterine tilts (anteverted, retroverted, sideverted).

Uterine Position	Anteverted	Retroverted	Sideverted
**Speculum mean offset (%)**	26.6+/-8.4	15.6+/-6.2	50+/-0
**Flat tip inserter mean offset (%)**	41.0+/-3.6	74.7+/-4.4	48.5+/-11.0
**Curved tip inserter mean offset (%)**	20.0+/-6.1	33.9+/-3.0	12.5+/-0

### Volunteer study

Testing of the flat tip and curved tip inserter variations were performed in fifteen volunteers. Volunteer demographics for the study are outlined in Table 5. For demographics, we looked at the number of vaginal deliveries, previous history with the speculum and use of tampons, as these could affect how easy and comfortable women would find the inserter. We also determined whether women thought the speculum was a barrier to screening and what they considered to be the top three most important factors for cervical cancer screening. Most of the volunteers were under the age of 29 and had not had any vaginal deliveries Approximately half of them had had fewer than two speculum examinations, however three- quarters of the volunteers were regular users of tampons/menstrual cups. Two-thirds of the women did not consider the speculum to be a barrier to cervical cancer screening, however one-third thought it was a small to medium barrier. Most of the women thought that adequate assessment of risk was most important in cervical cancer screening, while cost (three- quarters of the women) and comfort (half of women) were the second and third most important factors, respectively. Only one woman considered physician gender to be an important factor.

**Table 5:** Participants’ (n = 15) demographics for clinical study.

Characteristics	Response	Volunteers % (n)
Age, y	• ≤ 29 • 40–44 • 45–49	86.7 (13) 6.7 (1) 6.7 (1)
Number of vaginal deliveries	• 0 • 3	93.3 (14) 6.7 (1)
Number of cervical examinations with a speculum	• 0 • 1–2 • 3–5 • 6–10 • >10	13.3 (2) 33.3 (5) 26.7 (4) 6.7 (1) 20 (3)
Regular use of tampons	• Yes • No	77.8 (7/9) 22.2 (2/9)
Barrier of speculum to getting cervical cancer screening	• Not a barrier • Small barrier • Medium barrier • Large barrier	60 (9) 26.7 (4) 13.3 (2) 0 (0)
Top 3 most important considerations for cervical cancer examination	• Adequate assessment of risk • Travel distance to provider • Cost • Comfort during screening • Procedure time • Physician gender	93.3 (14) 46.7 (7) 73.3 (11) 53.3 (8) 26.7 (4) 6.7 (1)

**Fig 8:**
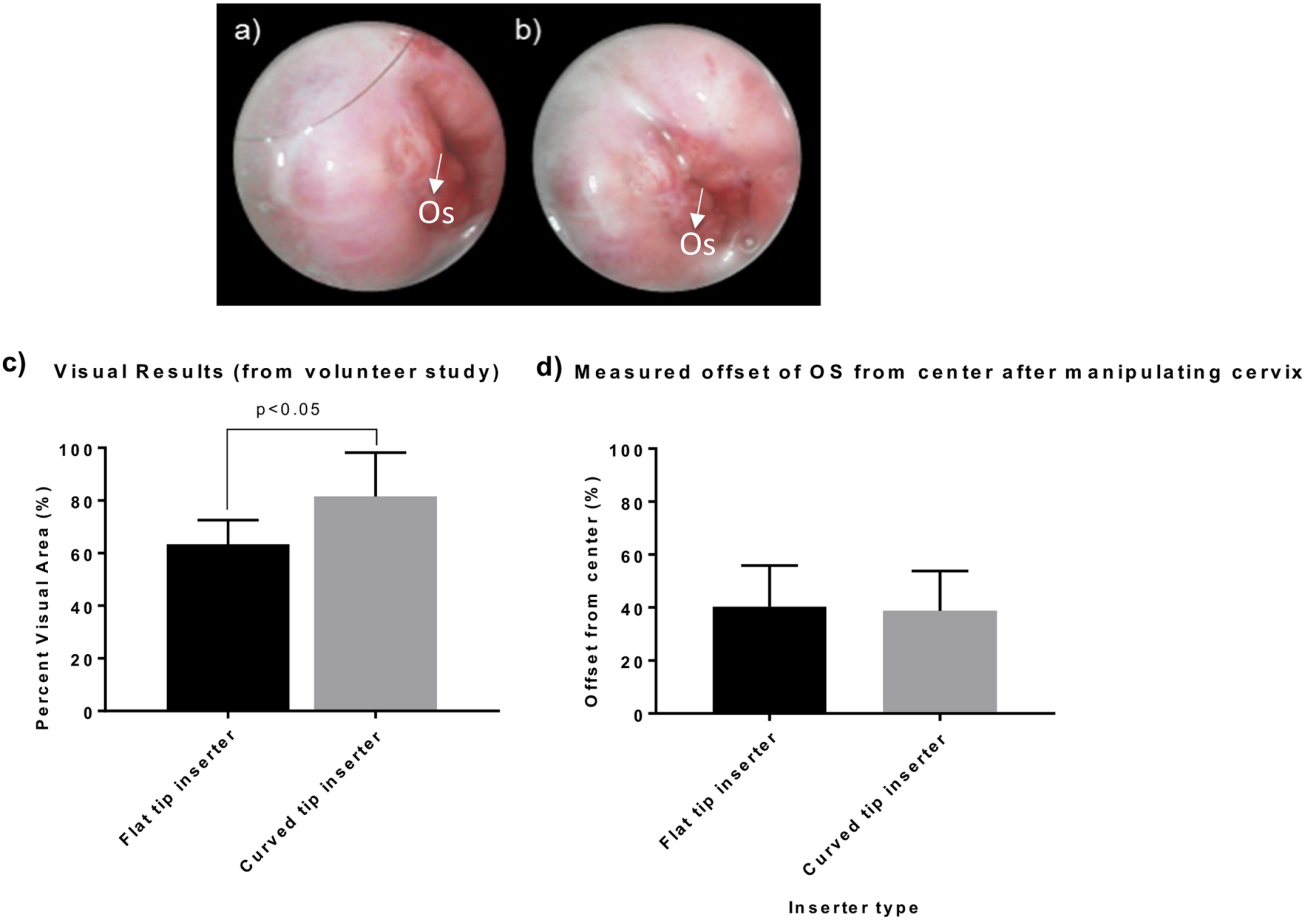
a) A sideverted cervix manipulated and imaged using the flat probe inserter, showing off centered cervix with os to the side. b) Same sideverted cervix manipulated and imaged using the curved tip probe inserter, showing a more centered cervix with os closer to the center. c) Graph comparing percent visual area for the flat and curved tip inserters. d) Graph comparing percent offset of the cervical Os from the center for the flat and curved tip inserters. Error bars are standard deviations.

Images were captured from twelve out of the fifteen volunteers. We were unable to find the cervices of two of the volunteers and cervix imaging from one volunteer was terminated due to personal reasons. Six of the acquired images were obtained from the flat tip inserter while six of the images were acquired from the curved tip inserter. Fig 8a and 8b show representative images from a single volunteer with a sideverted cervix using the flat tip inserter (8a) and the curved tip inserter (8b). The flat tip inserter was only able to sufficiently manipulate the cervix to a centered position and provide an adequate view (constitutes the entire os and a proportion of the surrounding cervix on all sides of the os) of the cervix for two out of six of the women assigned to it. In contrast, the curved tip inserter was able to center the cervix for imaging and provide an adequate view of the cervix for five out of six women, showing the os and an adequate section of the cervix. The mean percent visual area for the six patients who used the flat tip inserter was 63.4 +/- 9.1% and the mean percent offset was 40.25 +/- 15.6%. For the curved tip inserter, the mean percent visual area 81.5+/- 16.7% and the mean percent offset was 38.8+/-15% (Fig 8c and 8d)

**Fig 9:**
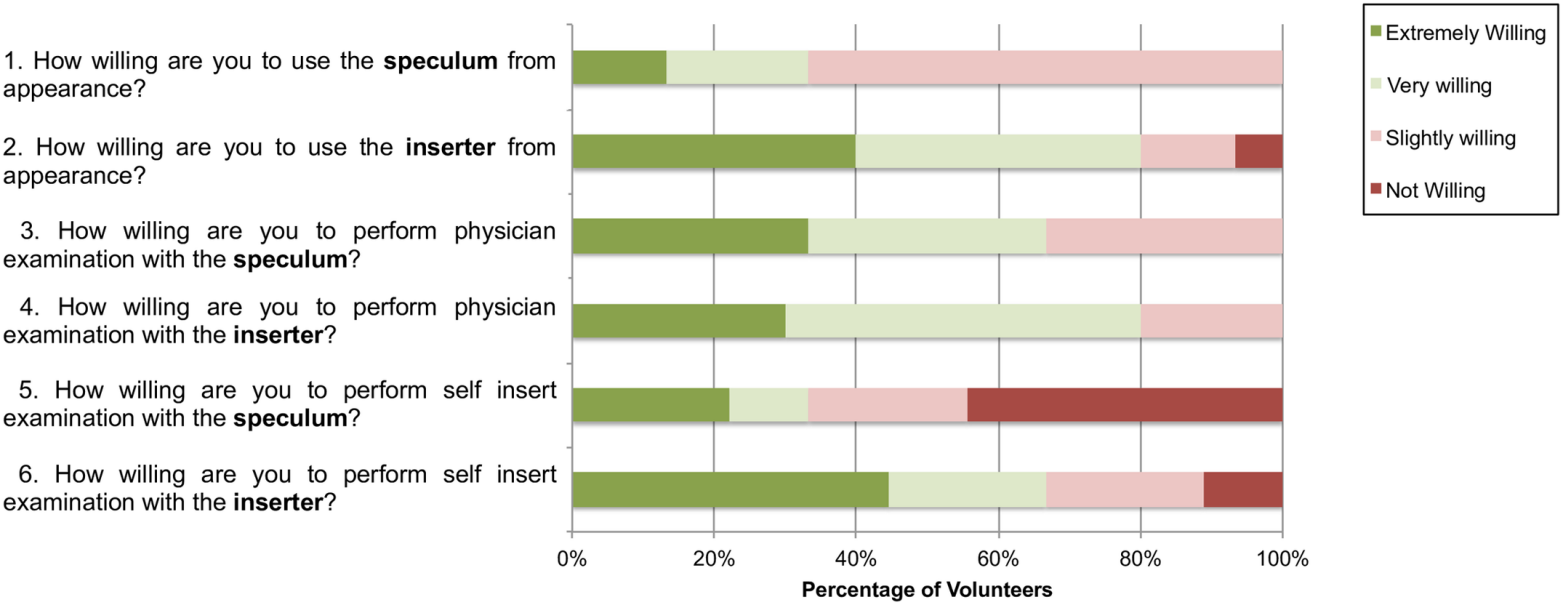
Pre-insertion questionnaire responses regarding attitudes towards cervical examinations from volunteers for self-testing (n = 15) showing responses to questions.

We provide a summary of pre- and post-insertion questionnaire results, assessing past experience with the speculum and vaginal examinations, and comfort and ease of use of the inserter compared to the speculum. The pre-insertion questionnaire responses (Fig 9) of fifteen volunteers showed that most women did not find the speculum physically appealing. Half of the women found the inserter physically more appealing than the speculum. About a quarter of the women were willing to have a physician perform an examination with the inserter rather than the speculum and about a third of the women were willing to perform self-insertion examination with the inserter rather than the speculum. Surprisingly, about a third of the women indicated a preference for physician examination over self-examination for both speculum and inserter scenarios.

**Fig 10:**
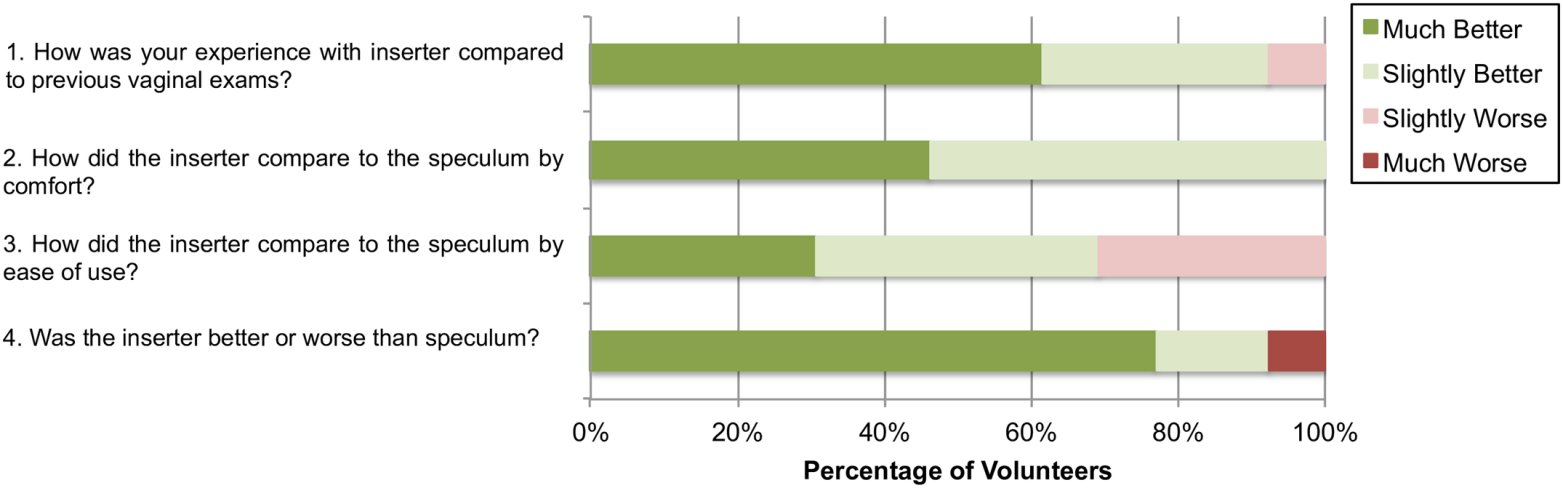
Post-insertion questionnaire responses from volunteer (n = 13) after self-testing, comparing the inserter to a speculum and self-examination to physician examination. Volunteers with no prior history of speculum examinations were excluded from answering these questions.

The post-insertion questionnaire responses (Fig 10) compared experiences with the inserter to previous experiences with the speculum. Two of the volunteers were excluded from participating in the post-insertion questionnaire as they had never had vaginal examinations with a speculum. Almost all (92.3%) of the women indicated that their experience with the inserter was “much better” or “slightly better” than previous experiences with the speculum. Only one volunteer indicated a slightly worse experience with the inserter than with the standard speculum. All of the volunteers indicated that, in terms of comfort, the inserter was either “slightly better” or “much better” than the standard speculum. Some comments from the surveys included: “this feels like using a tampon” and “if the inserter was what was used for testing I wouldn’t have been as hesitant to come in for an examination”. Almost all (92.3%) of the volunteers indicated that the inserter was overall “slightly better” or “much better” than the speculum, with only one volunteer indicating that it was “much worse” than the speculum. Even though the general experience with the inserter was better than that with the speculum, about a third (70%) of the women indicated that it was harder to perform self-examination on themselves than have a physician perform the examination.

## Discussion

We have developed an Inserter as an alternative to the standard speculum for use with our POC*ke*T Colposcope to enable speculum-free, self-insertion, and image capture of the cervix. The device has a slim tubular body of 1.0 cm diameter opening up to a curved funnel-like tip measuring approximately 2.5 cm in diameter. The materials used for manufacturing the body of the inserter will be made of similar material as standard of care plastic speculums and future work will include a soft-touch silicone tip to further reduce any vaginal discomfort, particularly for patients with vaginal atrophy. We also expect that the device will be lubricated, per standard-of-care before insertion to prevent tearing of the vaginal epithelium. The inserter has a channel through which a 2MP mini USB camera with LED illumination fits to enable cervix image capture. The channel also enables acetic acid/Lugol’s iodine application and insertion of swabs for Pap smear sample collection. The camera interfaces via a USB cable to a phone, tablet, or computer, all of which provide power to the camera and enable image capture. Thus, the camera only requires a charged phone, tablet, or computer to operate, but does not require AC power or a separate battery source. Illumination is provided by built-in white LEDs from the camera. We envision that the camera/inserter device will replace the standard colposcope and speculum in the cervical cancer screening paradigm. The cost of the 2MP USB camera is $54. This is much less compared to the cost of a standard-of-care Leisegang Optik2 digital colposcope (up to US$20,000). We estimate that the projected cost of the inserter under mass production will be less than a dollar about the same price as a plastic speculum. This provides a significantly lower cost for colposcopy examinations. Combined with the POC*ke*T Colposcope, the inserter can also be used for HPV and Pap smear sample collection by the patient herself or clinician since the cytobrushes used have similarly small diameters.

The final inserter design was selected out of four initial designs as optimal due to results obtained from mechanical simulations and phantom tests. The mechanical tests demonstrated a high factor of safety (90.9) for the selected inserter under vaginal pressure simulations and the phantom tests demonstrated adequate unobstructed visualization and ability to manipulate the cervix. The inserter was compared to the Graves speculum. Visualization between the two, though not significantly different for normal positions and retroverted uterus tilts, showed that the inserter was significantly better for sideverted and anteverted positions (p<0.00001). Pressures used for testing were obtained from a study using a vaginal pressure catheter to record vaginal pressure in fifteen women without risk of prolapse during different exercises and states of rest [24]. Testing with the range of pressures for women in a supine position demonstrated that, even though visualization from the speculum and inserter are comparable at lower vaginal pressures, the inserter is superior at higher pressures, where there is more obstruction of the cervix by the vaginal walls. Hence, our device has the potential for more comfortable cervical examination for larger women or women with multiple prior pregnancies who have lax vaginal walls that might require use of a large speculum or a side wall retractor, which adds to the pain and discomfort of the procedure. Our design also enables manipulation of the cervix for cervical imaging of women with tilted uteri, a condition that affects about 20% of women and is difficult and painful when using the standard speculum for manipulation.

Even though pre-insertion questionnaires from the clinical studies indicated that the majority of the volunteers did not consider the speculum to be a barrier to cervical cancer screening, this may be likely due to women feeling more concerned over adequate assessment of risk rather than comfort during the examination. Results from post-insertion questionnaires found that 92.3% thought the inserter was slightly better or much better than the speculum, and 100% of volunteers indicated that the inserter was slighter or much more comfortable. Additional questionnaire responses found that even though from past experience women were willing to use the speculum, from appearance most women found the speculum intimidating. Responses also revealed that women would be more likely to perform self-insertion examinations with the inserter than the speculum. One woman indicated in her comments that she could probably perform the exam herself at home once she had practiced it a couple of times.

The fixed viewing area offered by the inserter may be problematic in viewing cervices of different sizes, particularly those of larger sizes. Even though the inserter is limited by its smaller diameter, it can image a 2.5 cm diameter, the average size of a cervix, and can capture an image of the transformation zone and sufficient cervical area around the central os (which is < 0.5cm diameter). Since cervical cancer originates from the transformation zone, this would be a sufficient area for an adequate evaluation in most cases. There may also be avenues for the production of inserters of varying sizes to match cervix size. The lack of ability to see the squamocolumnar junction with the inserter and camera is another limitation but this is also a limitation to current methods of visual inspection of the cervix. It is therefore not a replacement for gold standard pathology, where cells from the squamocolumnar junction are removed for pathology. However, our device can aid in guiding biopsies and has merit for places that lack gold standard biopsy. Even though the inserter enables comfortable, self-insertion and cervical image capture, there is still a need to improve the quality of the images taken for comparable quality to standard colposcopes. The USB camera used for this study’s experiments is a low-cost, 2MP off-the-shelf camera with no added features and substandard image quality and is different from the POC*ke*T Colposcope camera we have previously published on [22]. The POC*k*eT Colposcope, a low-cost, pen-sized, transvaginal digital colposcope, has been clinically validated by physicians worldwide to have image quality concordance comparable to high-end state-of-the-art digital colposcopes [22]. The POC*ke*T Colposcope uses consumer grade lighting sources and a 5MP camera in a tampon form factor for white and green light colposcopic imaging. Our next steps will be to modify the inserter to accommodate the POC*ke*T Colposcope for added image quality for cervical cancer diagnosis and clinically validate that the use of the POC*ke*T Colposcope with the inserter does not deteriorate image quality and cancer screening and prevention.

In addition to image capture of the cervix for colposcopy examinations, the channel of the inserter enables the insertion of cotton swabs and cytobrushes typically used during HPV and Pap smear collection. Procedures to obtain these samples typically involve the use of a speculum. Due to the small diameters of the swabs and brushes used (≤ 7mm) the inserter could provide a more comfortable alternative to the speculum for cervix sample collection. Additionally the camera/inserter technology can be used at home by women to view the cervical os for determination of cervical dilation during labor and detection of false labors. Future studies will compare cervix samples collected with the inserter and the speculum for equivalency to enable use for self-sampling.

## Conclusion

We have developed a probe-like inserter device as an alternative to the speculum for use with the POC*ke*T Colposcope to image the cervix for cervical cancer screening. The inserter currently works with a 2MP mini USB camera with LED illumination. It has been mechanically tested with simulations and shown to provide a high factor of safety (90.9). Phantom testing demonstrated the ability to withstand a range of supine vaginal pressures and manipulate cervices for image capture. Testing the inserter *in vivo* with fifteen volunteers showed that the inserter enables image capture of the cervix in a more comfortable manner and with less intimidation than the speculum. Our results have encouraged us to proceed with optimizing the inserter device for the 5MP POC*ke*T Colposcope designed by our group, for higher image quality and optimal cervical cancer diagnosis.

## Supporting information

S1 File Images of prototype CAD designs. This contains the images of the billed expander, silicone expander, flat-tip inserter and curved-tip inserter.
(ZIP)

S2 File Images of custom phantom cervix taken at different pressures for each prototype. (ZIP)

S3 File Images of custom phantom cervix taken at different tilted uterus positions. (ZIP)

S4 File Volunteer questionnaire form. (PDF)

S5 File Volunteer questionnaire responses. (XLSX)

S6 File Cervix images from clinical study with volunteers. (ZIP)
