# Supplementary figures and images for: Design and preliminary analysis of a vaginal inserter for speculum-free cervical cancer screening
Source: PLoS One. 2017 May 31;12(5):e0177782. doi: 10.1371/journal.pone.0177782 (PMC5451045; doi:10.1371/journal.pone.0177782)

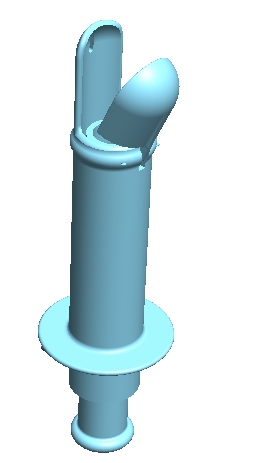

Supplement: S1 File — This contains the images of the billed expander, silicone expander, flat-tip inserter and curved-tip inserter. (ZIP) [file pone.0177782.s001.zip › S1 Fig 1. Billed Expander.PNG]

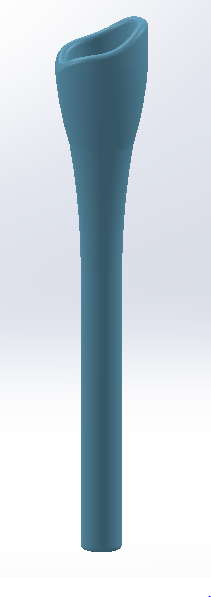

Supplement: S1 File — This contains the images of the billed expander, silicone expander, flat-tip inserter and curved-tip inserter. (ZIP) [file pone.0177782.s001.zip › S1 Fig 2. Curved Tip Inserter.PNG]

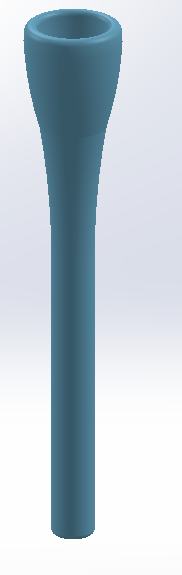

Supplement: S1 File — This contains the images of the billed expander, silicone expander, flat-tip inserter and curved-tip inserter. (ZIP) [file pone.0177782.s001.zip › S1 Fig 3. Flat Tip Inserter.PNG]

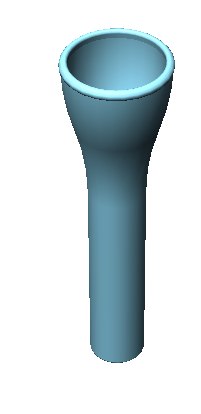

Supplement: S1 File — This contains the images of the billed expander, silicone expander, flat-tip inserter and curved-tip inserter. (ZIP) [file pone.0177782.s001.zip › S1 Fig 4. Silicone Inserter.PNG]

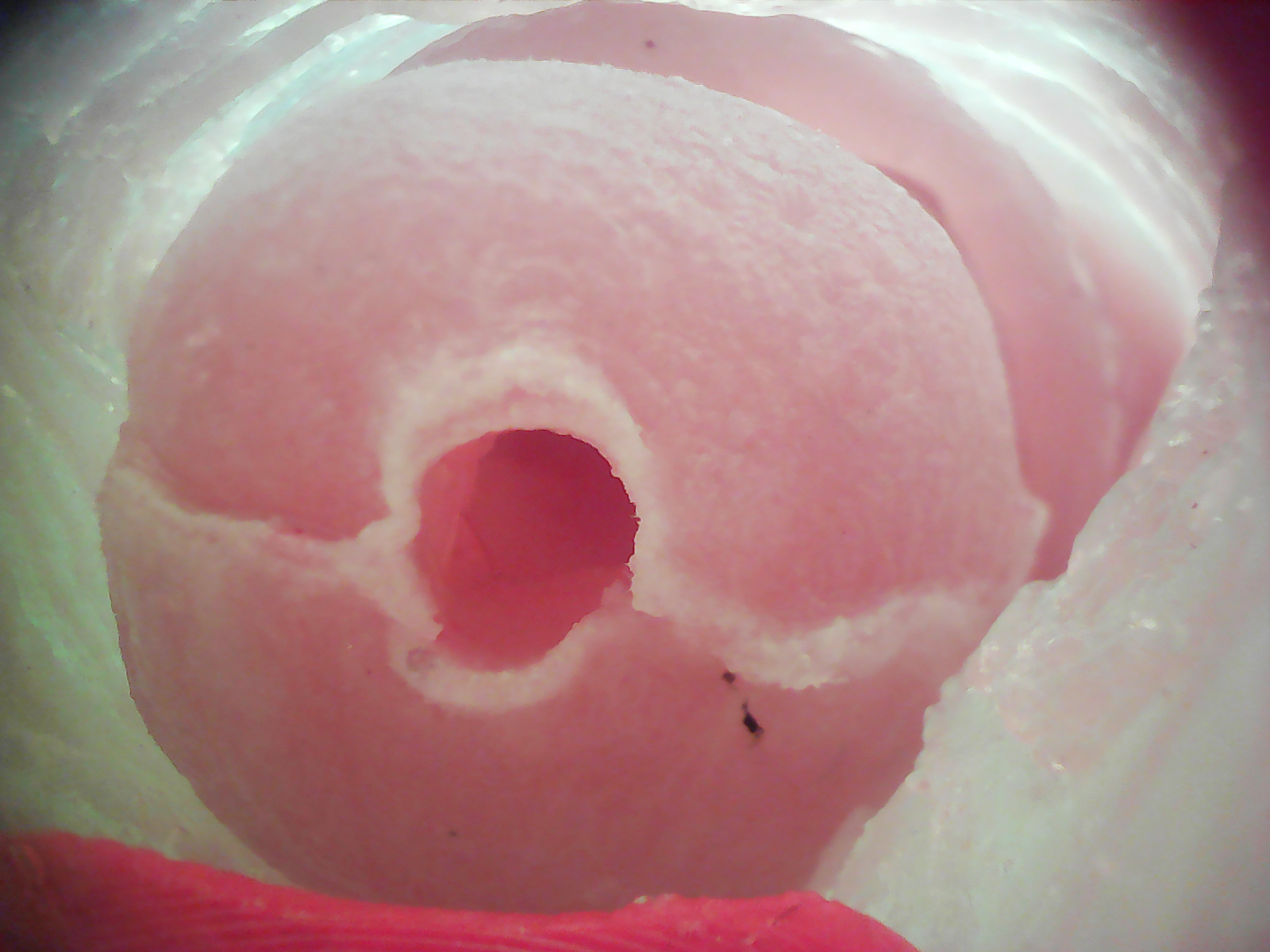

Supplement: S2 File — (ZIP) [file pone.0177782.s002.zip › S2 Billed expander at 0.01 cmH2O.tif]

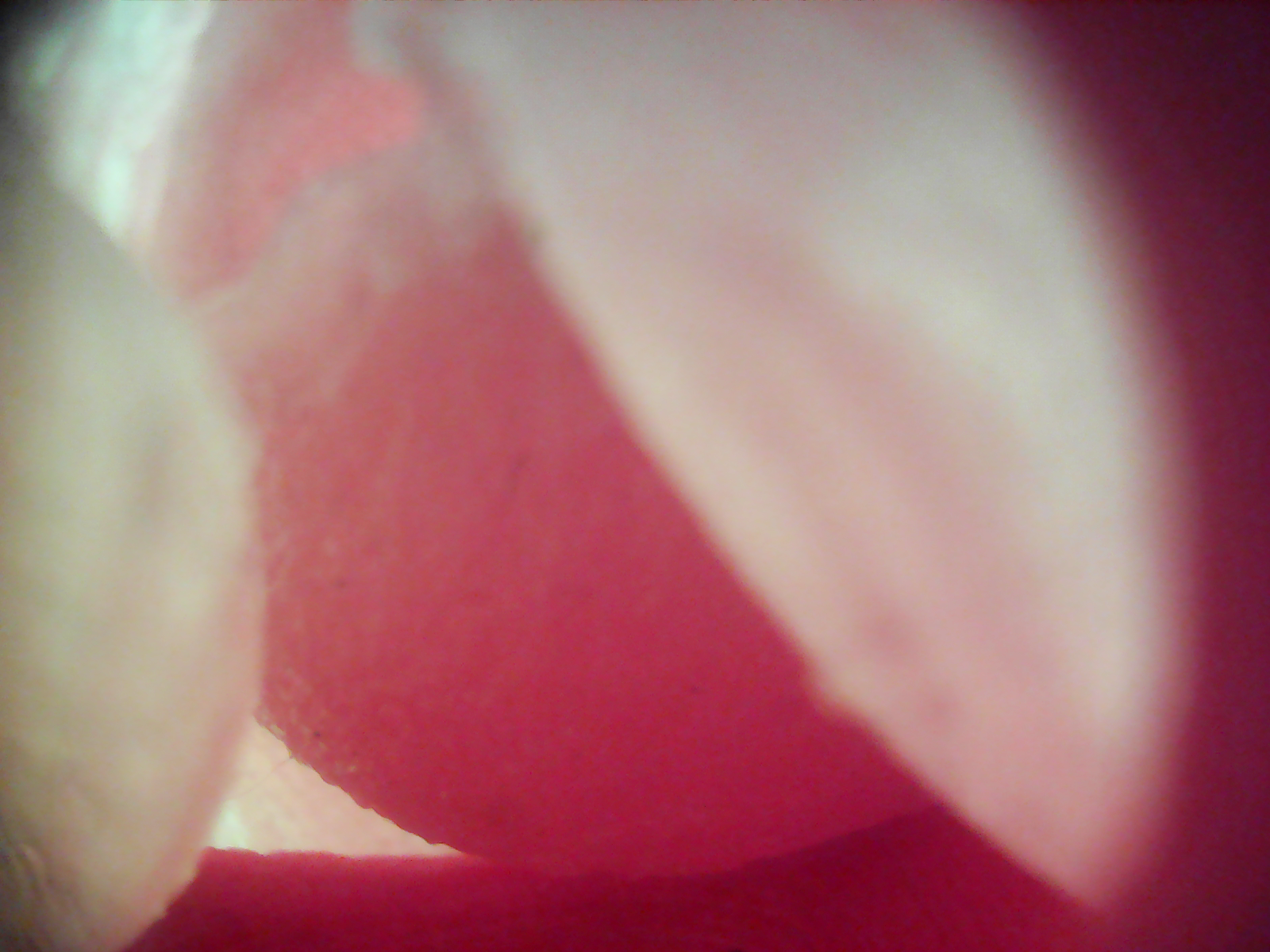

Supplement: S2 File — (ZIP) [file pone.0177782.s002.zip › S2 Billed expander at 10 cmH2O.tif]

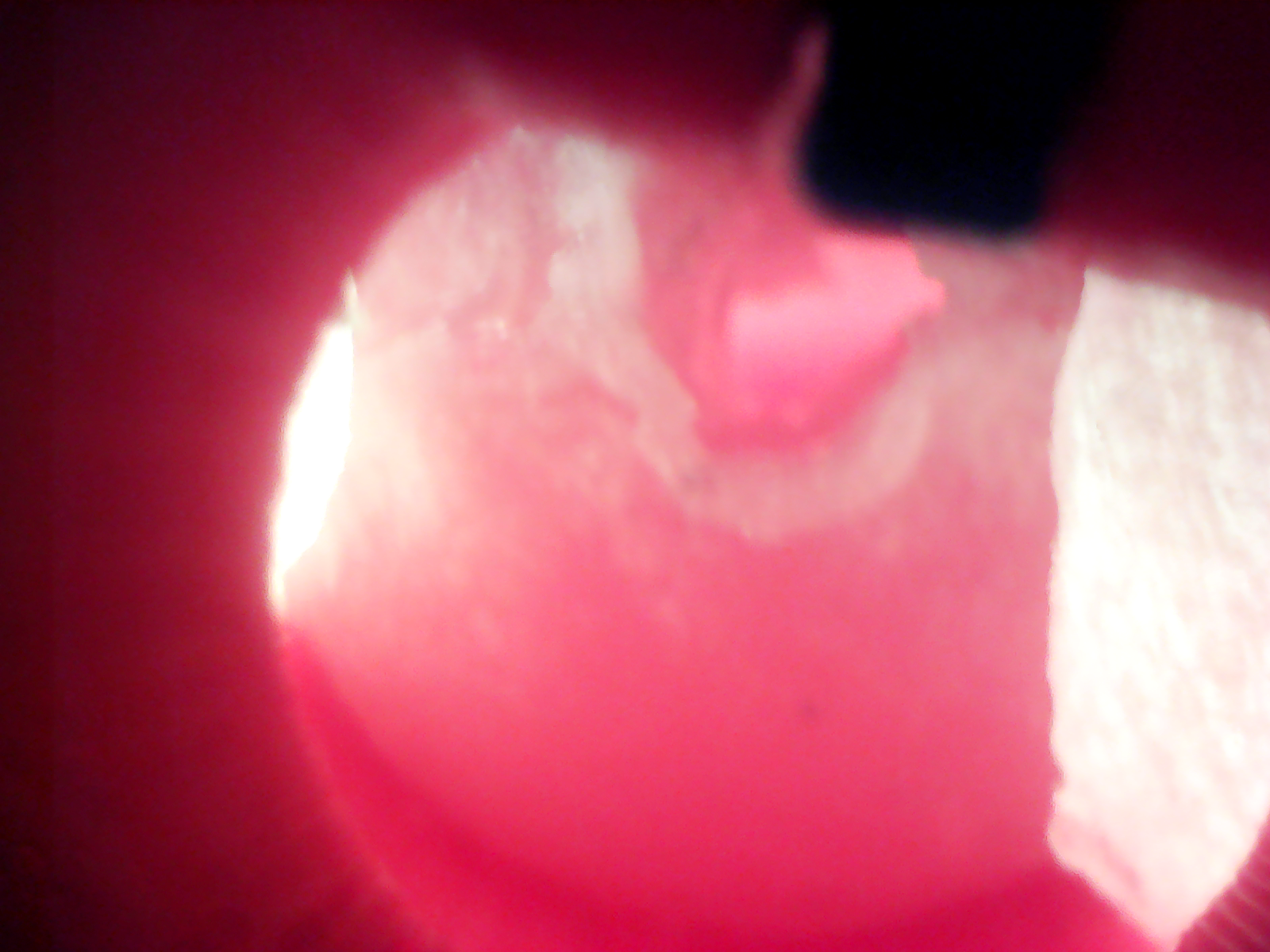

Supplement: S2 File — (ZIP) [file pone.0177782.s002.zip › S2 Billed expander at 15 cmH2O.tif]

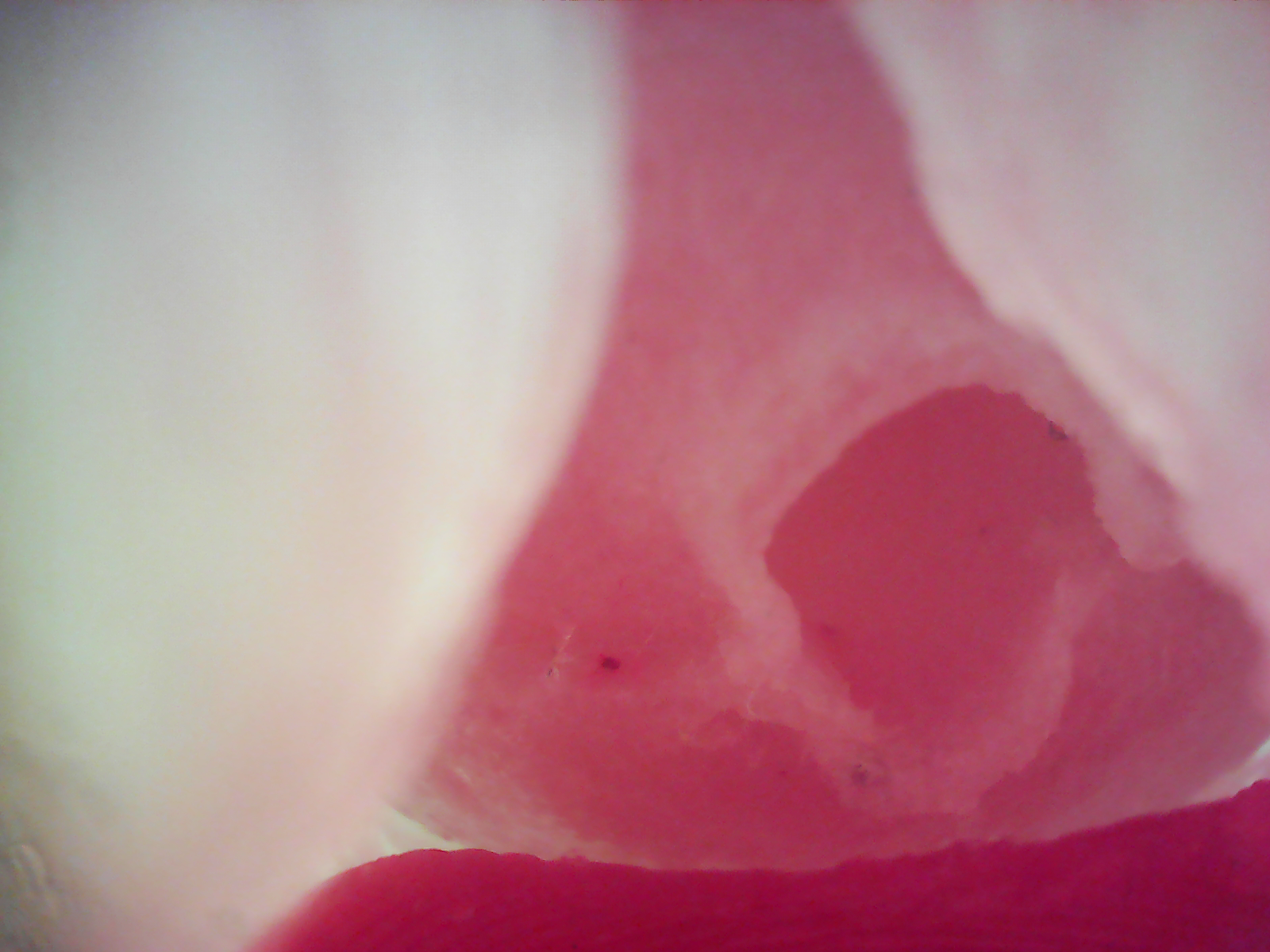

Supplement: S2 File — (ZIP) [file pone.0177782.s002.zip › S2 Billed expander at 5 cmH2O.tif]

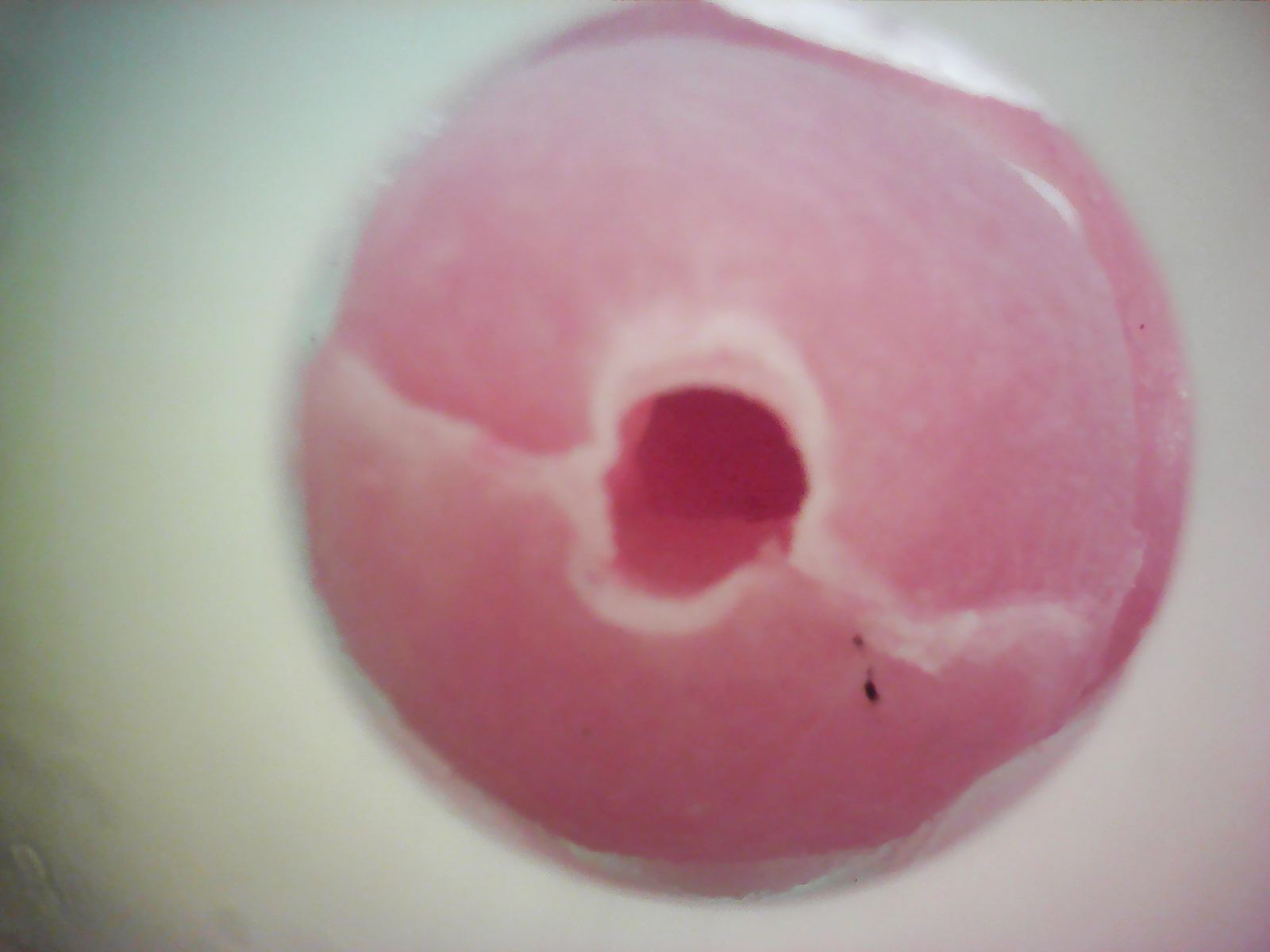

Supplement: S2 File — (ZIP) [file pone.0177782.s002.zip › S2 Flat-tip inserter at 0.01 cmH2O.tif]

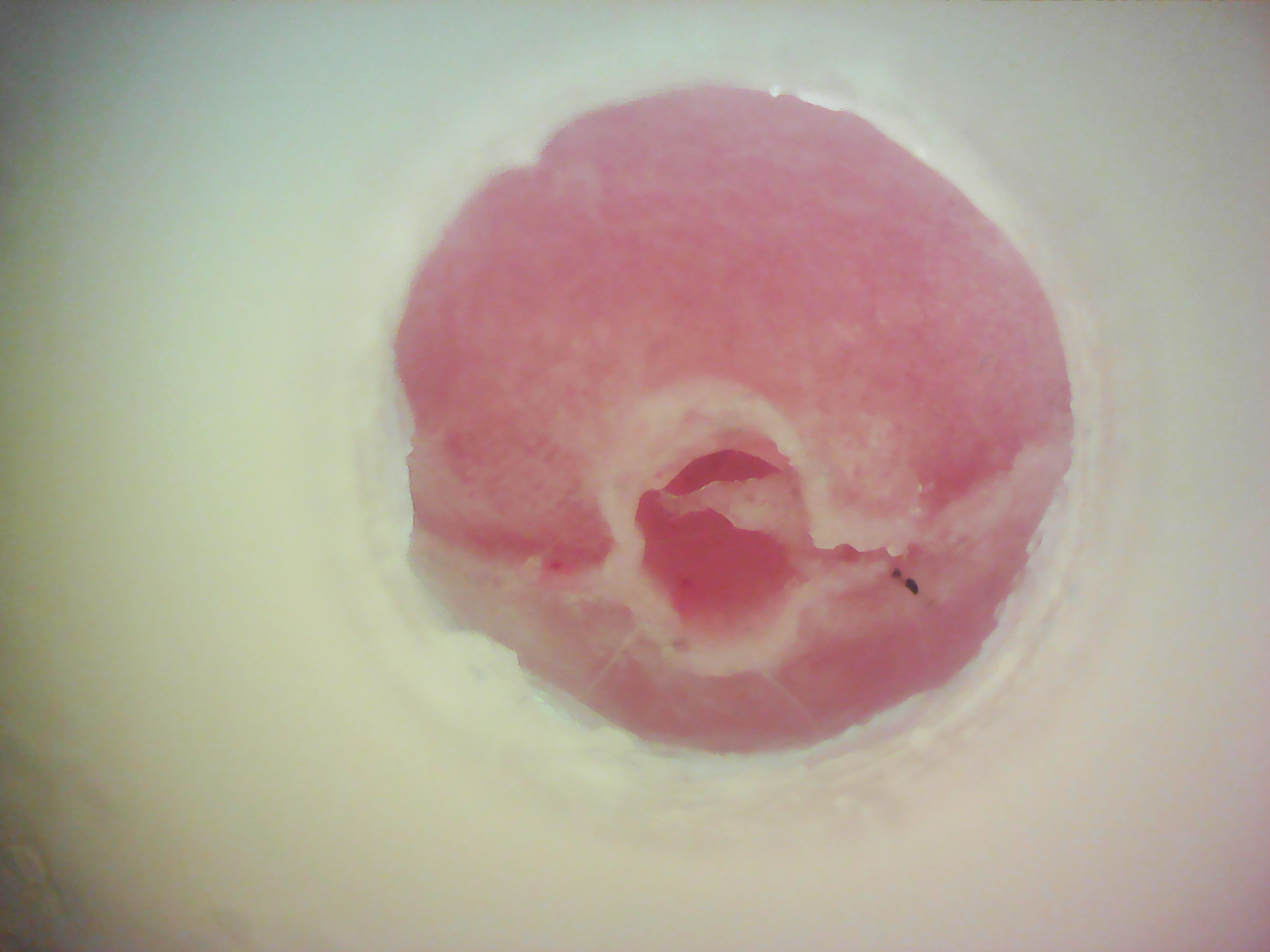

Supplement: S2 File — (ZIP) [file pone.0177782.s002.zip › S2 Flat-tip inserter at 10 cmH2O.tif]

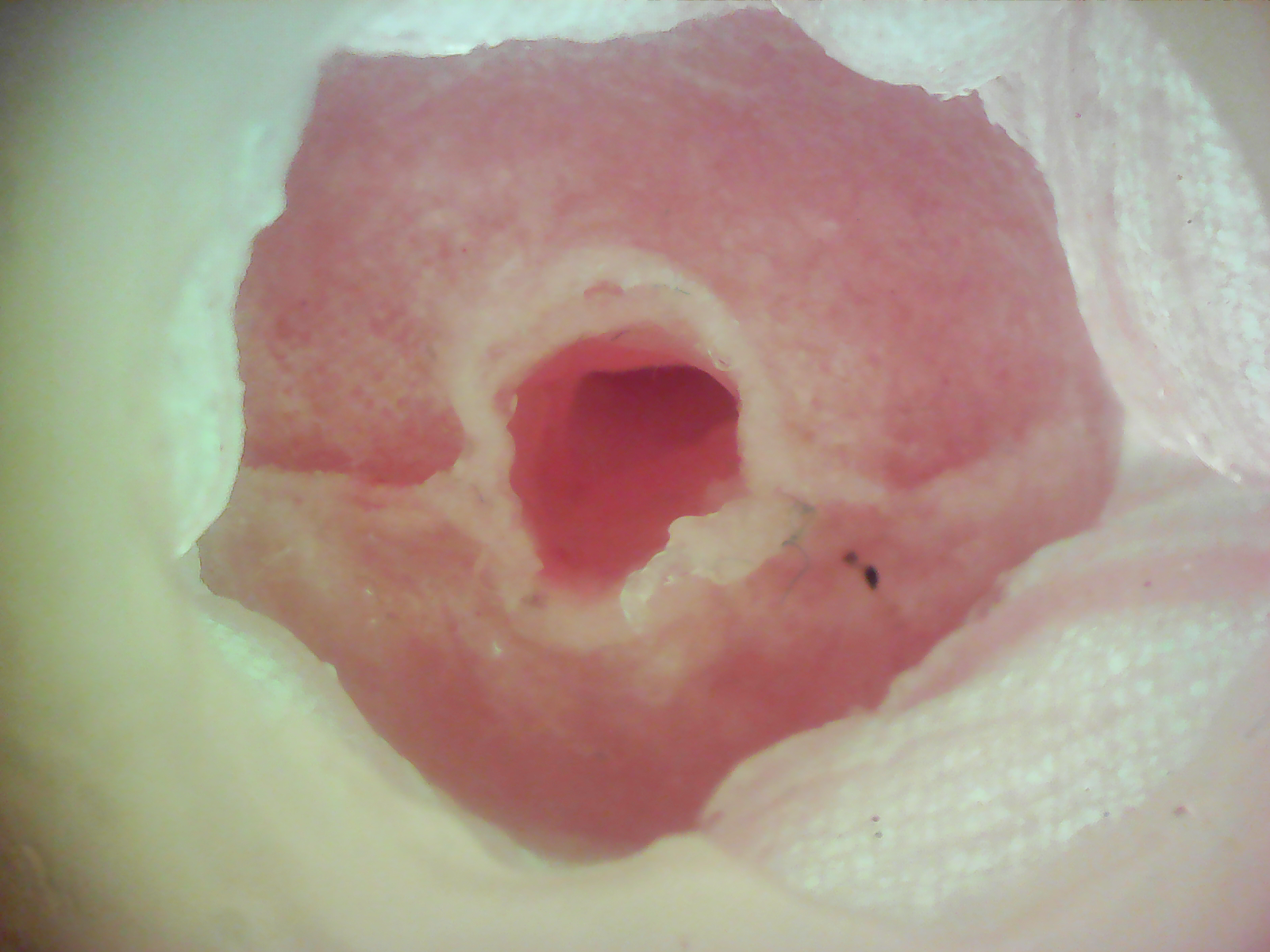

Supplement: S2 File — (ZIP) [file pone.0177782.s002.zip › S2 Flat-tip inserter at 15 cmH2O.tif]

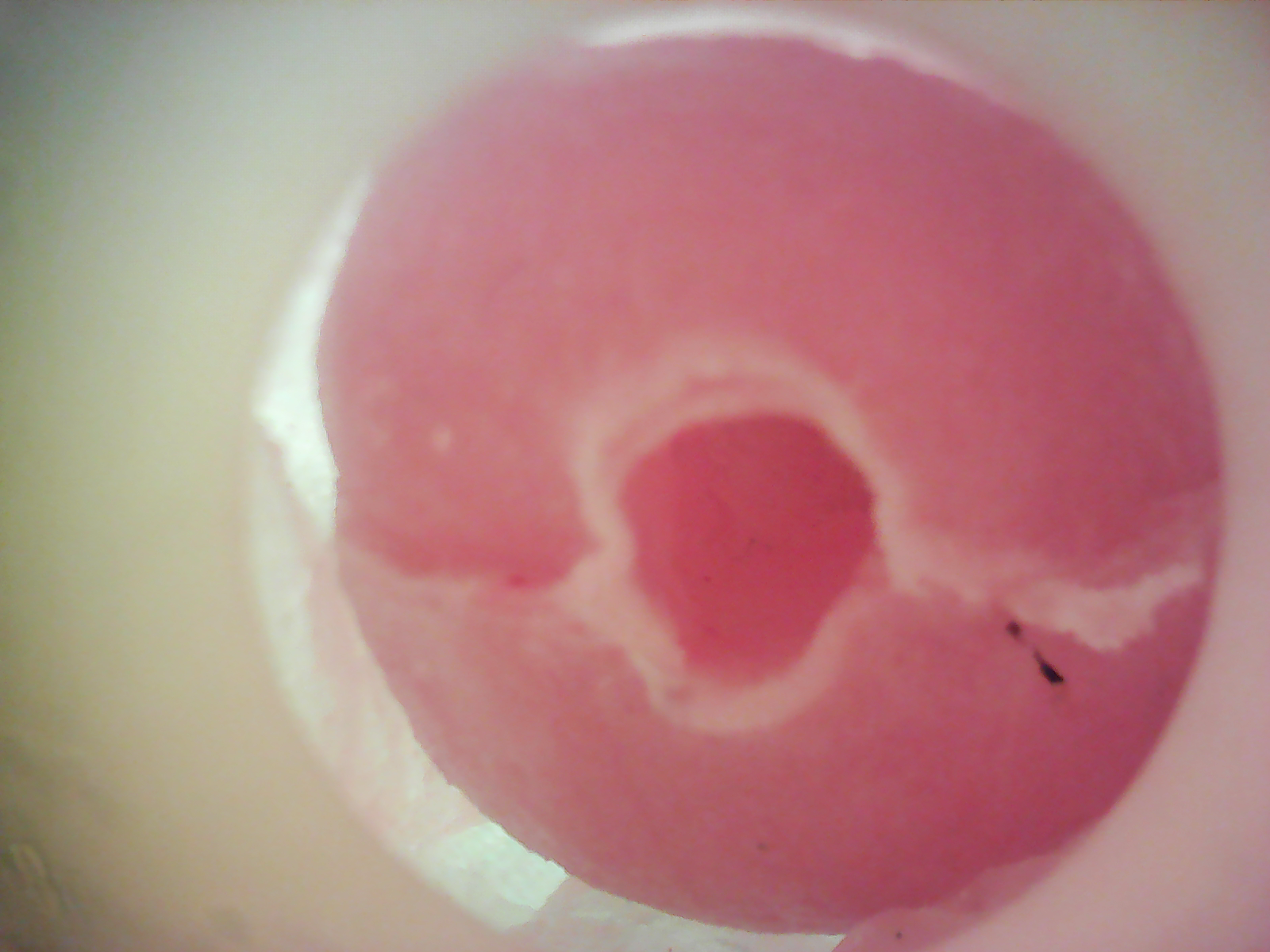

Supplement: S2 File — (ZIP) [file pone.0177782.s002.zip › S2 Flat-tip inserter at 5 cmH2O.tif]

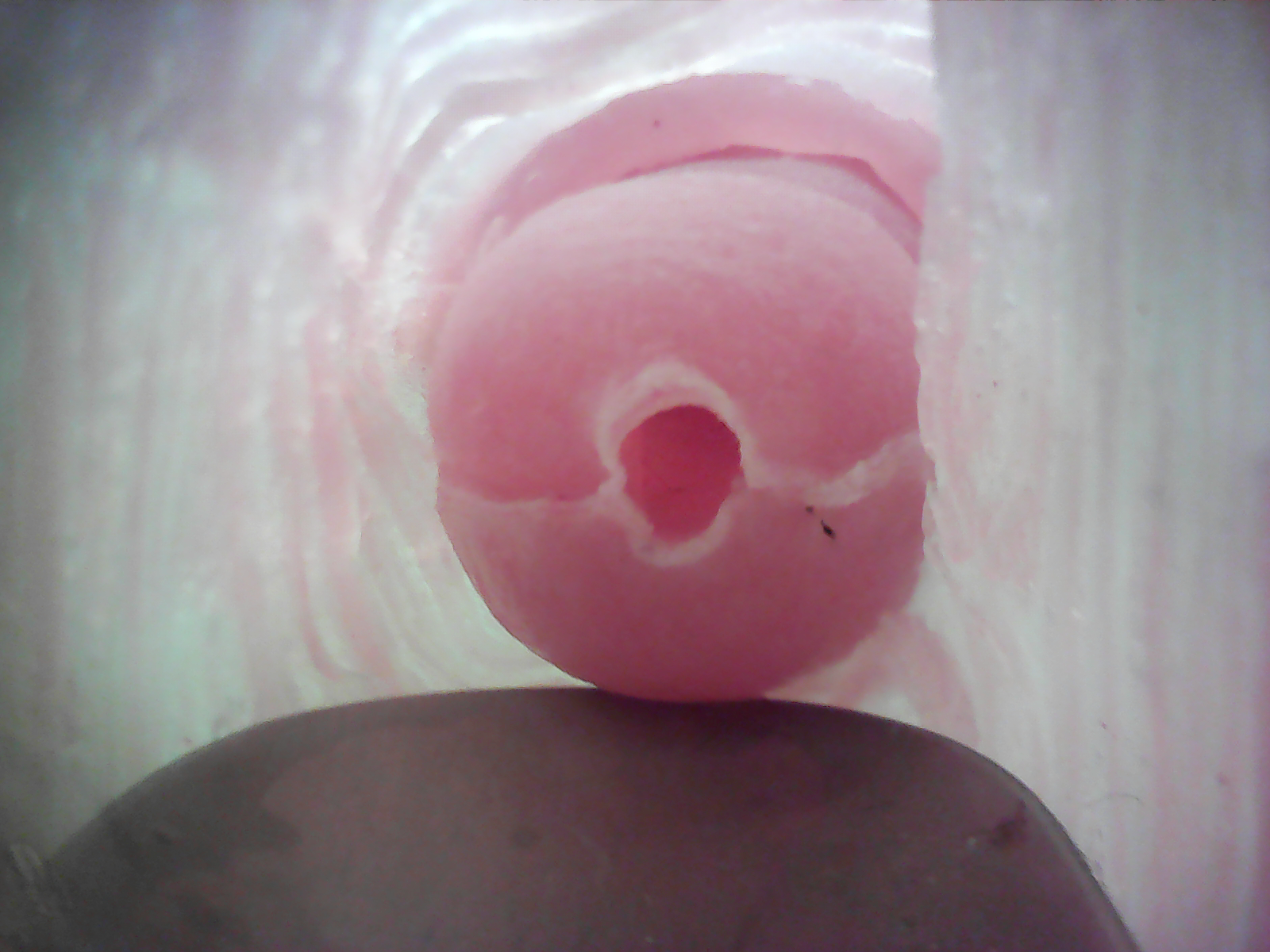

Supplement: S2 File — (ZIP) [file pone.0177782.s002.zip › S2 Graves speculum at 0.01 cmH2O.tif]

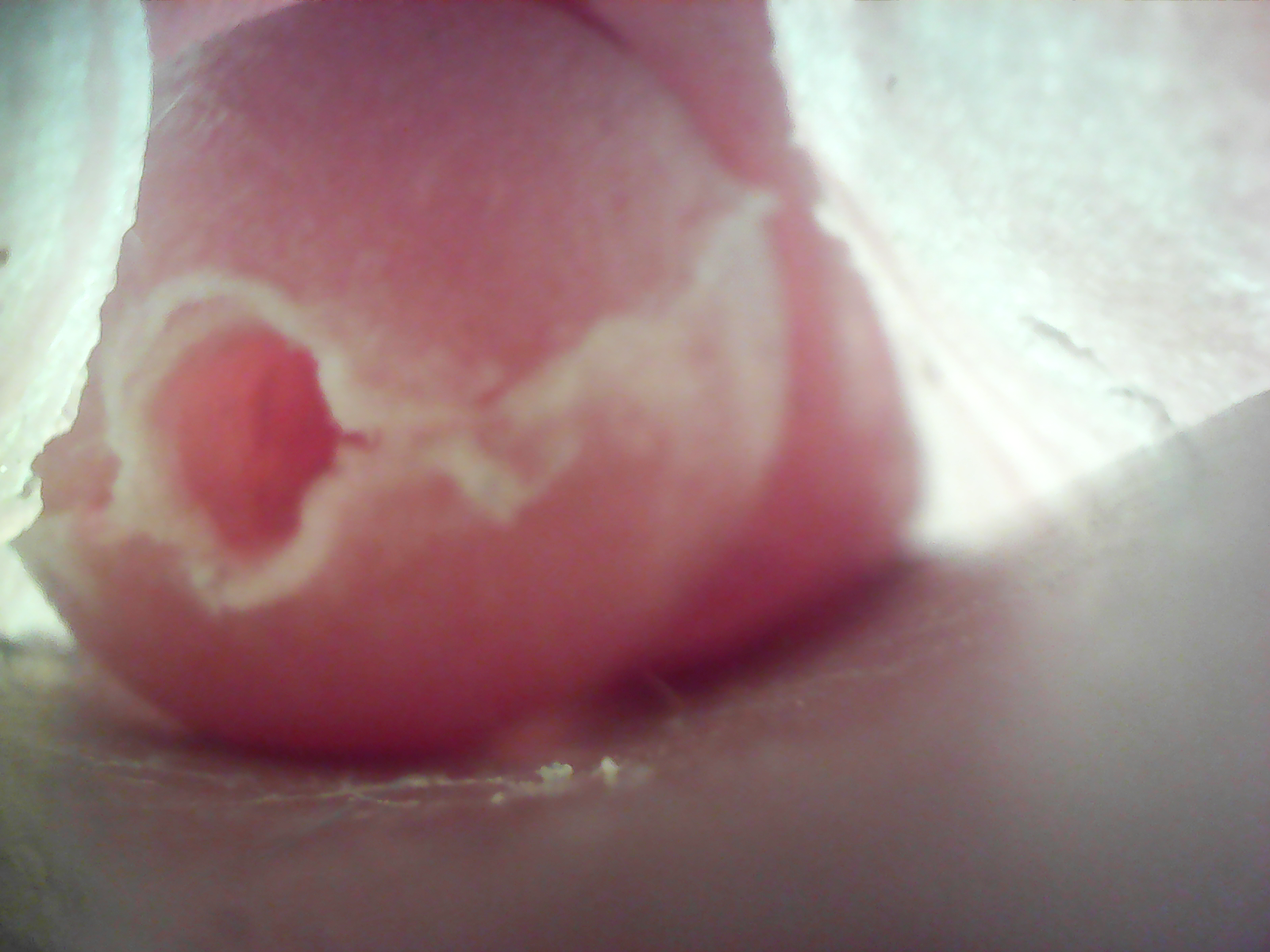

Supplement: S2 File — (ZIP) [file pone.0177782.s002.zip › S2 Graves speculum at 10 cmH2O.tif]

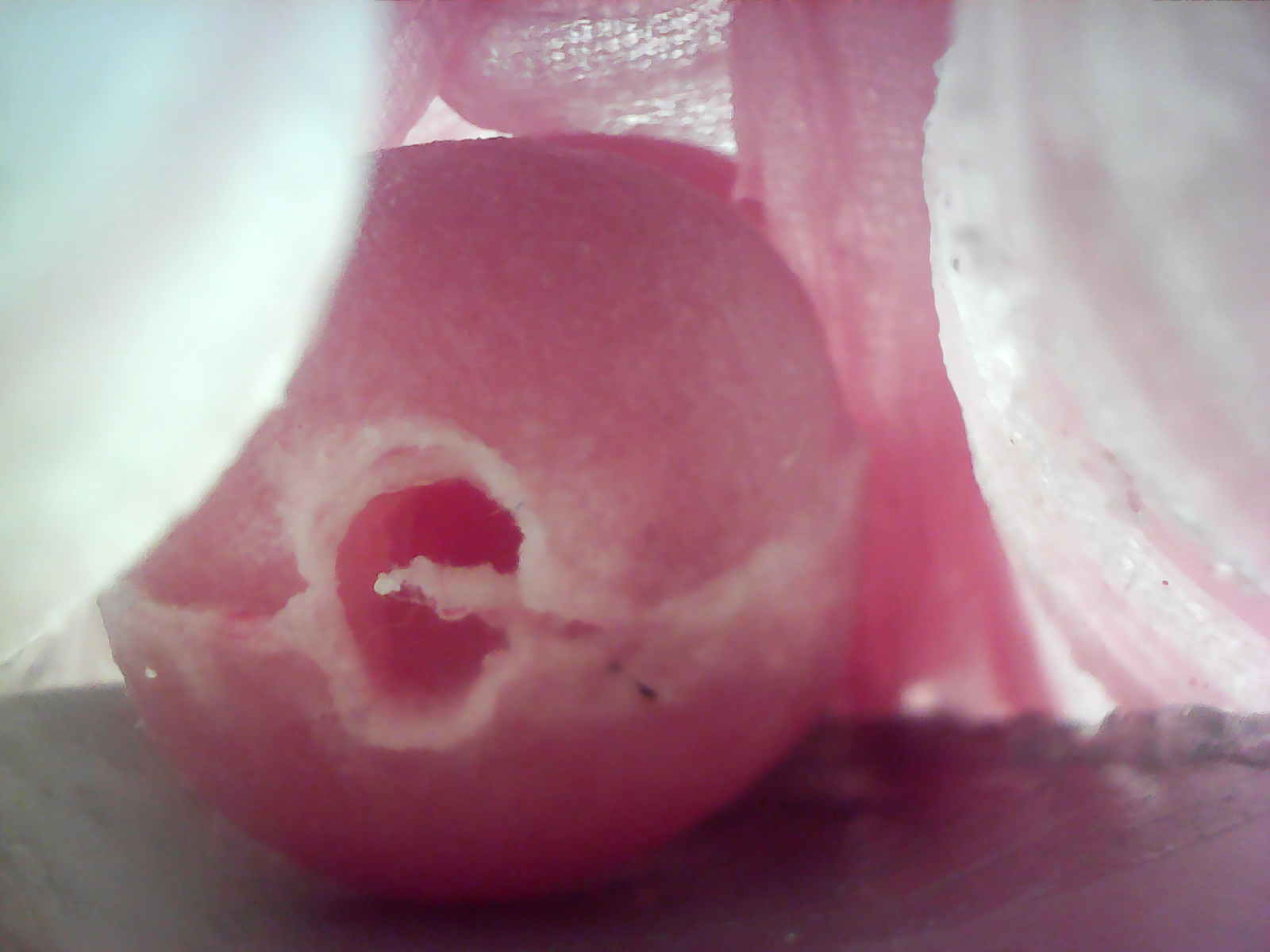

Supplement: S2 File — (ZIP) [file pone.0177782.s002.zip › S2 Graves speculum at 15 cmH2O.tif]

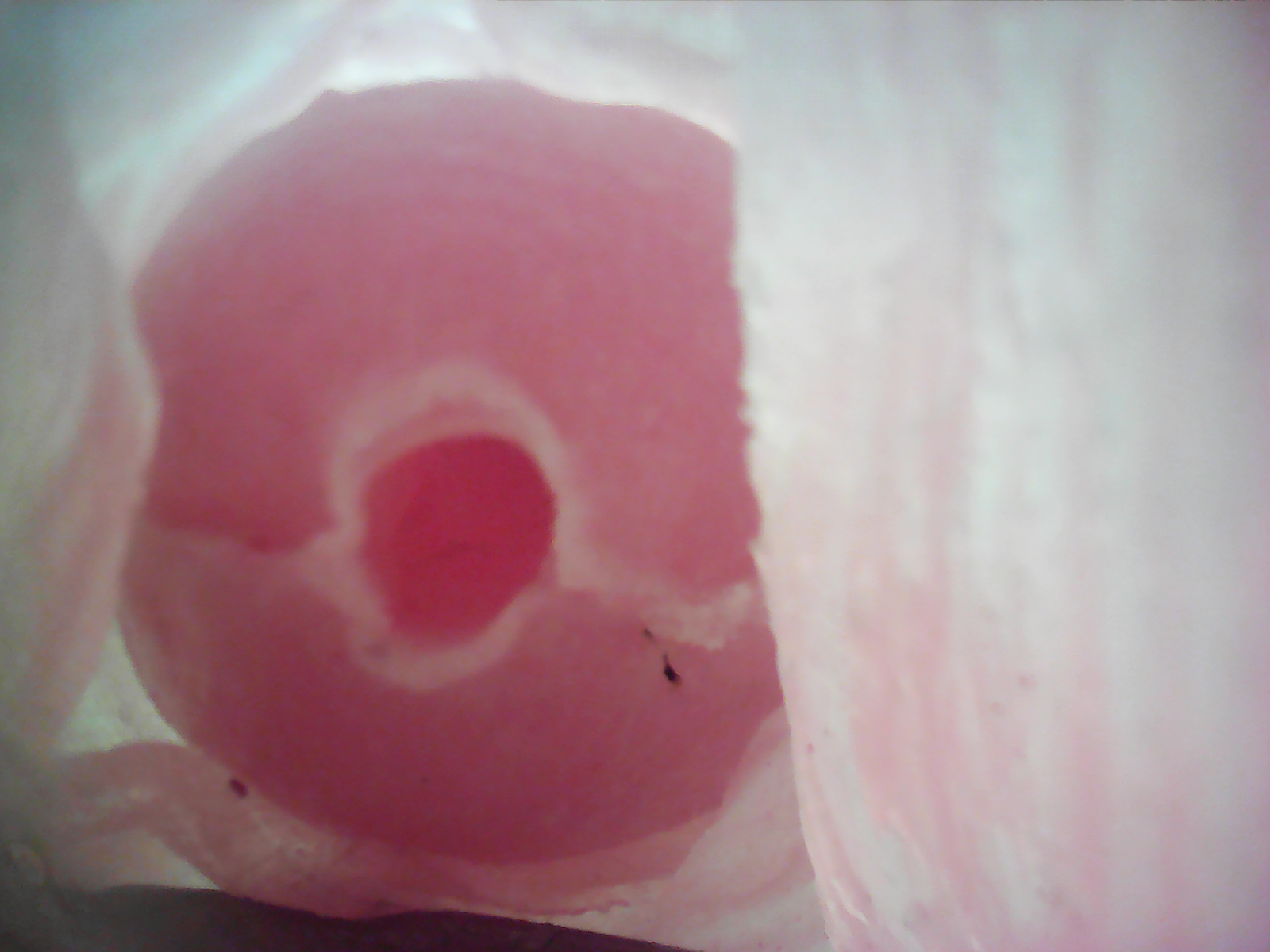

Supplement: S2 File — (ZIP) [file pone.0177782.s002.zip › S2 Graves speculum at 5 cmH2O.tif]

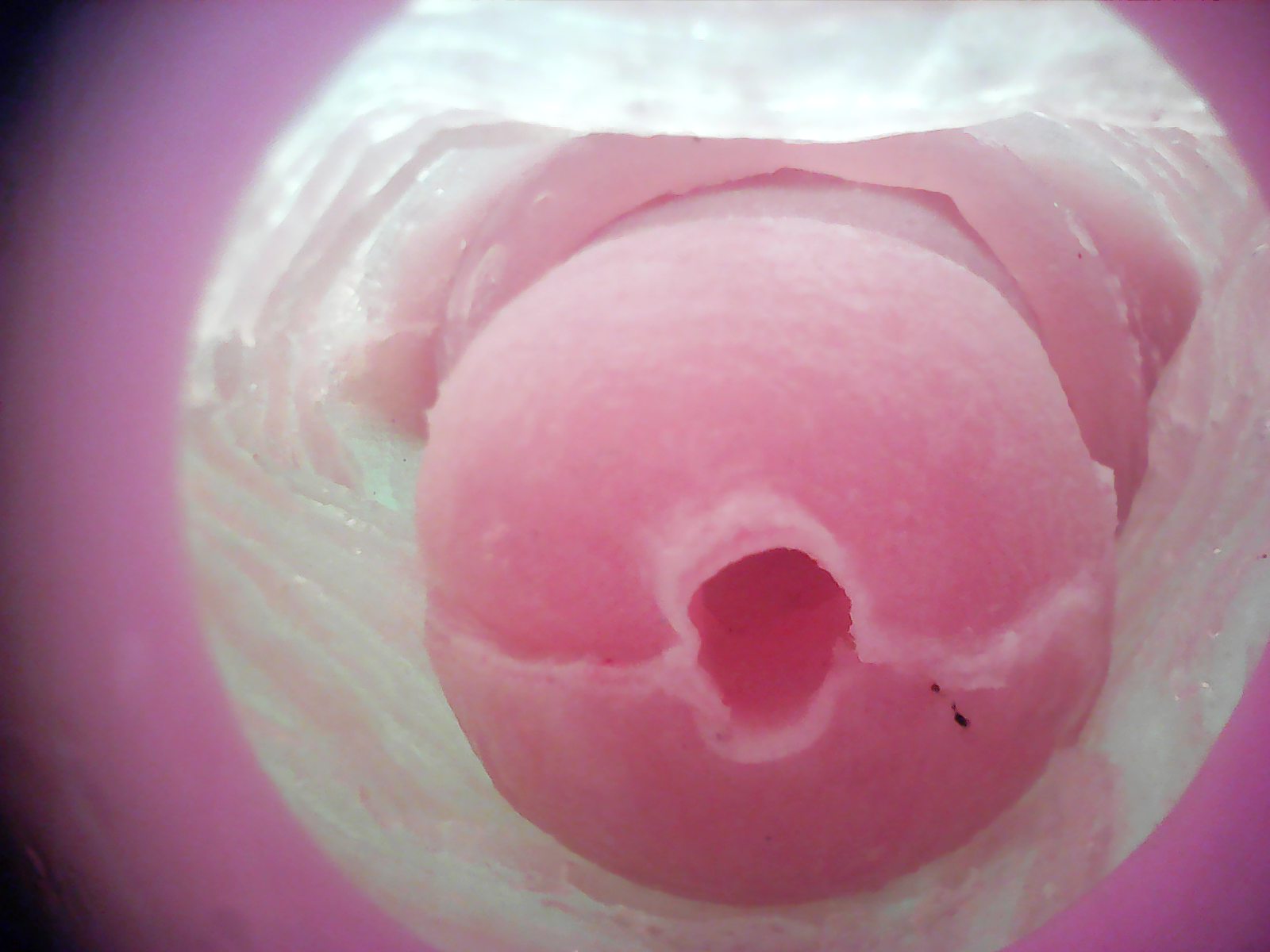

Supplement: S2 File — (ZIP) [file pone.0177782.s002.zip › S2 Silicone expander at 0.01 cmH2O.tif]

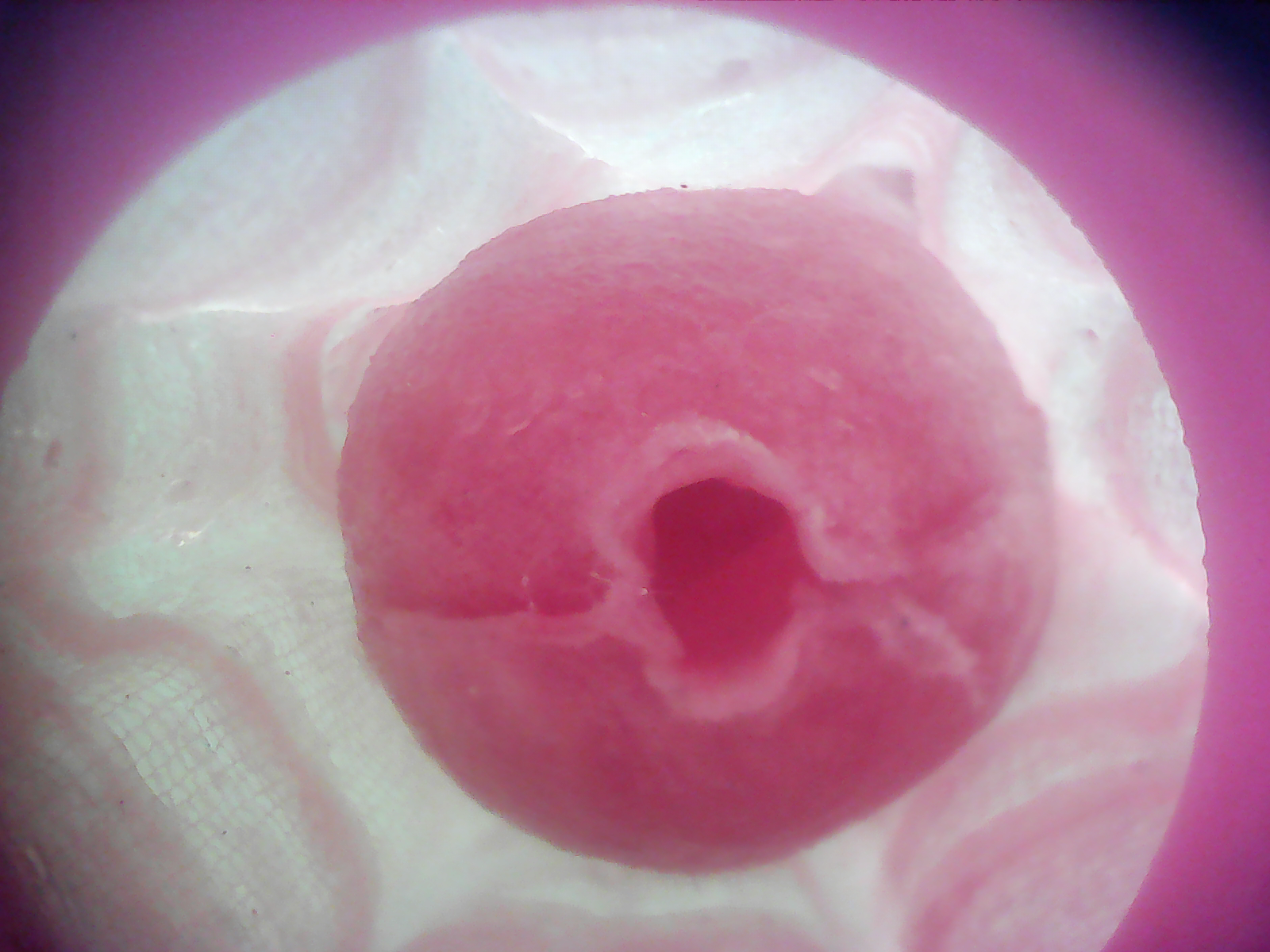

Supplement: S2 File — (ZIP) [file pone.0177782.s002.zip › S2 Silicone expander at 10 cmH2O.tif]

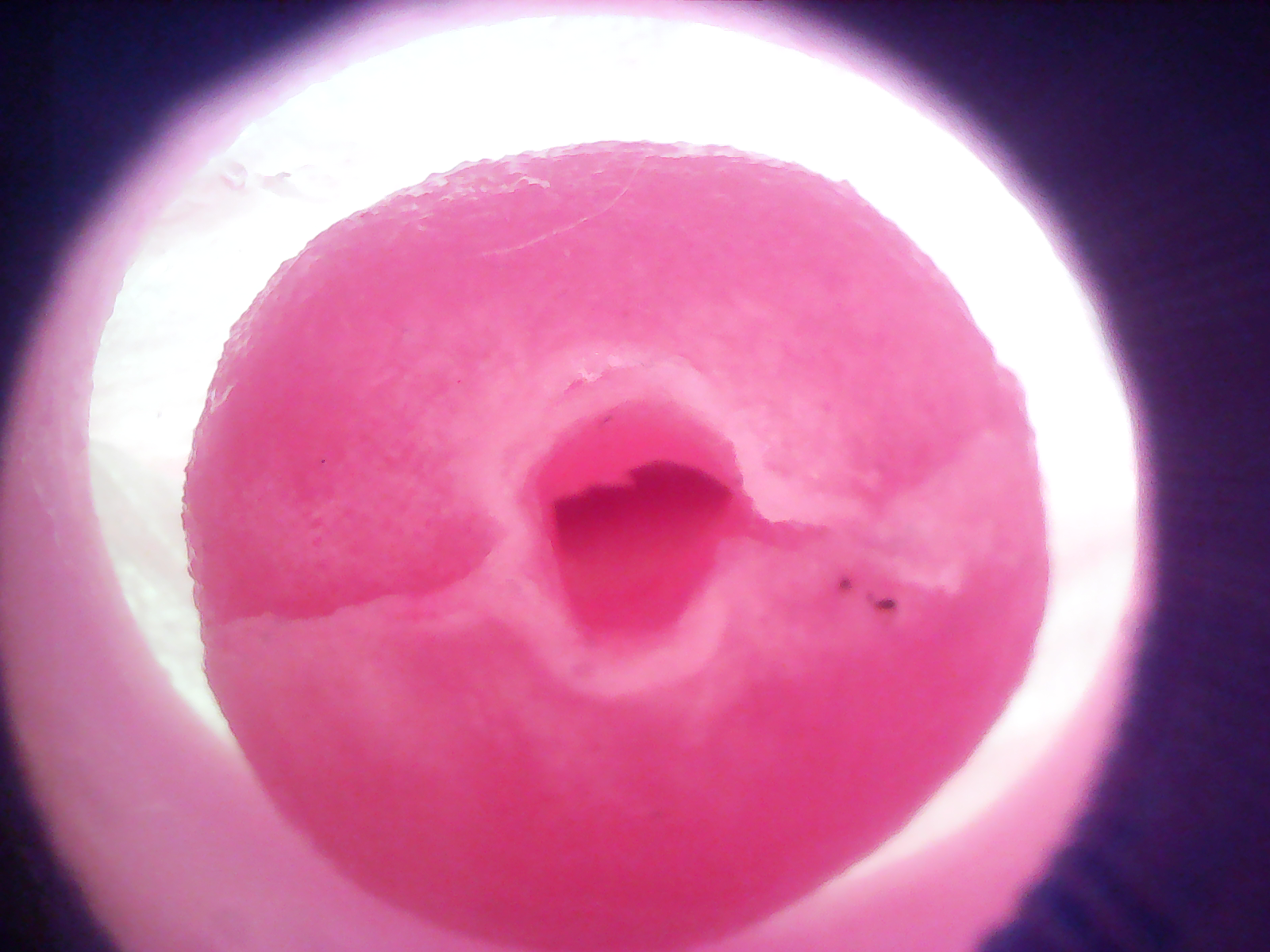

Supplement: S2 File — (ZIP) [file pone.0177782.s002.zip › S2 Silicone expander at 15 cmH2O.tif]

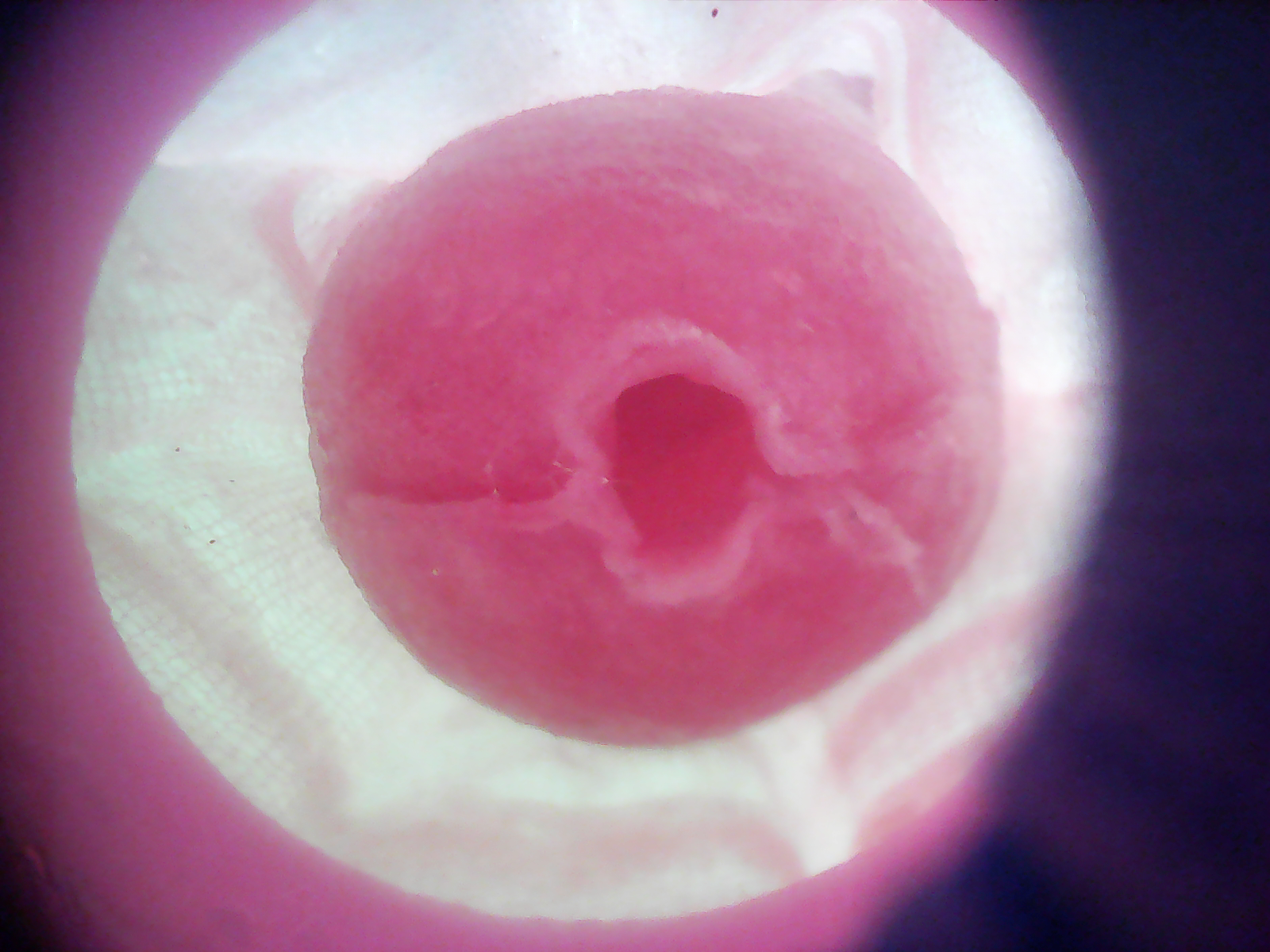

Supplement: S2 File — (ZIP) [file pone.0177782.s002.zip › S2 Silicone expander at 5 cmH2O.tif]

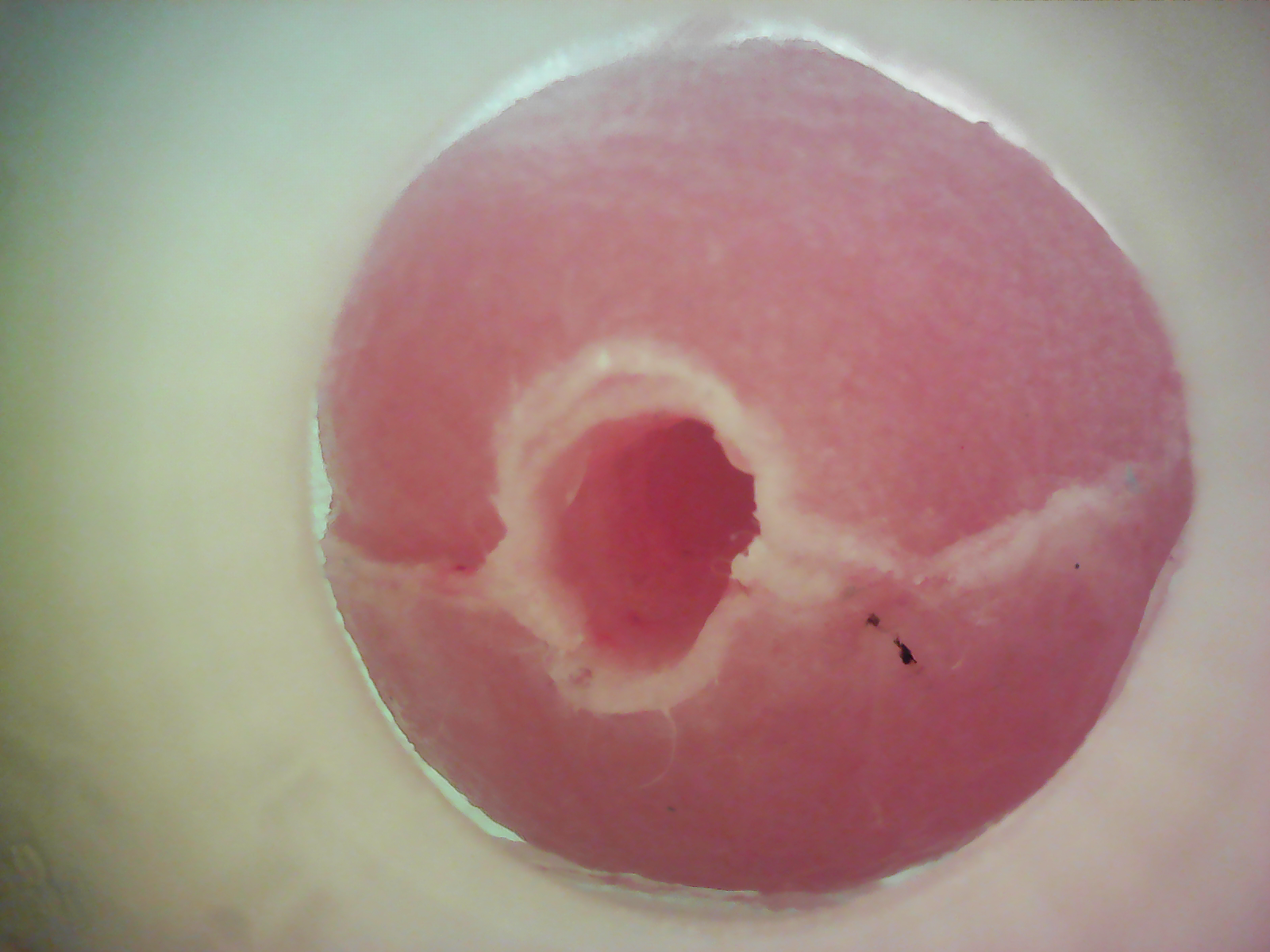

Supplement: S3 File — (ZIP) [file pone.0177782.s003.zip › S3 Fig 1. Curved-tip inserter anteverted.tif]

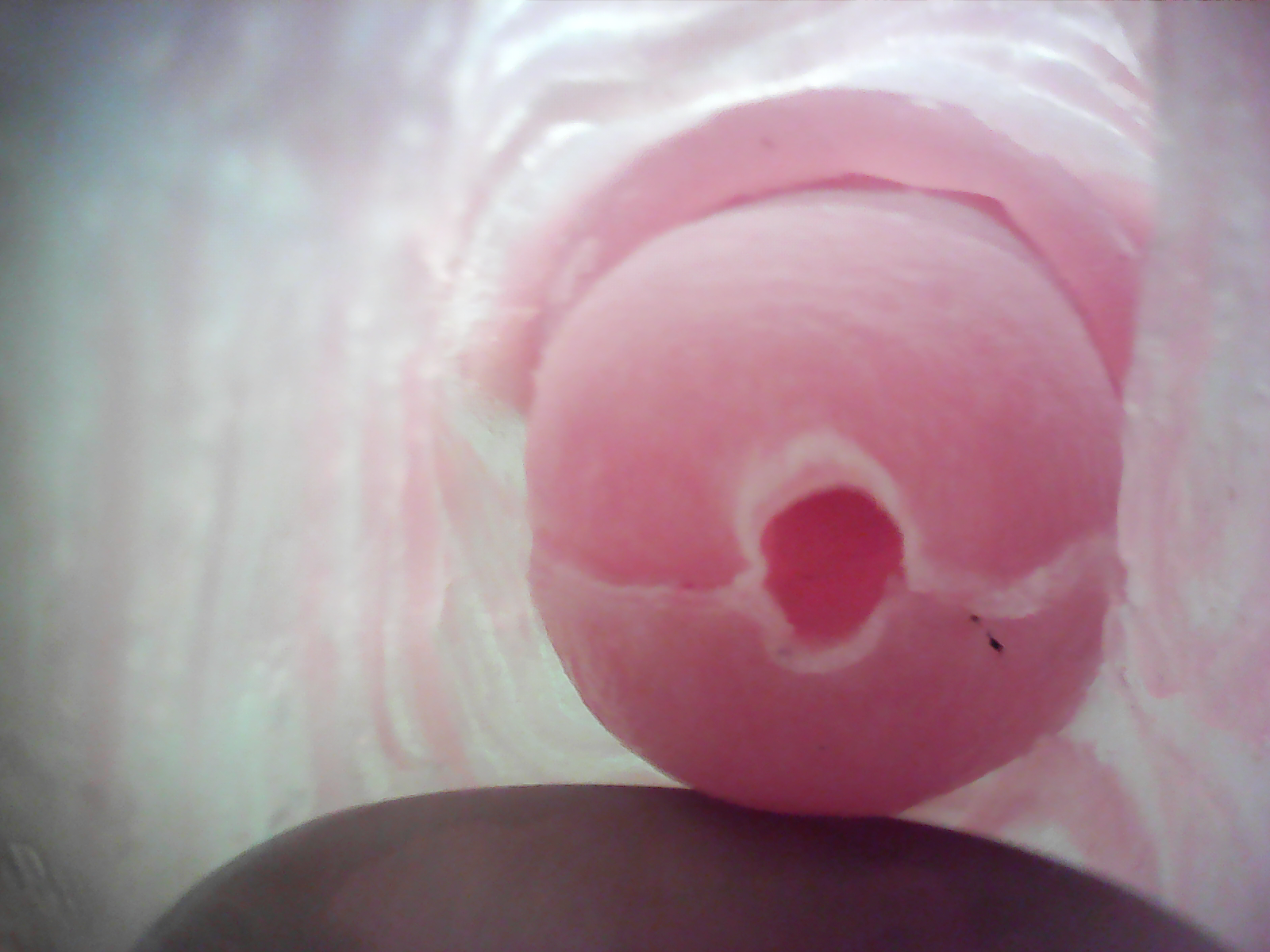

Supplement: S3 File — (ZIP) [file pone.0177782.s003.zip › S3 Fig 10. Graves speculum mid-position.tif]

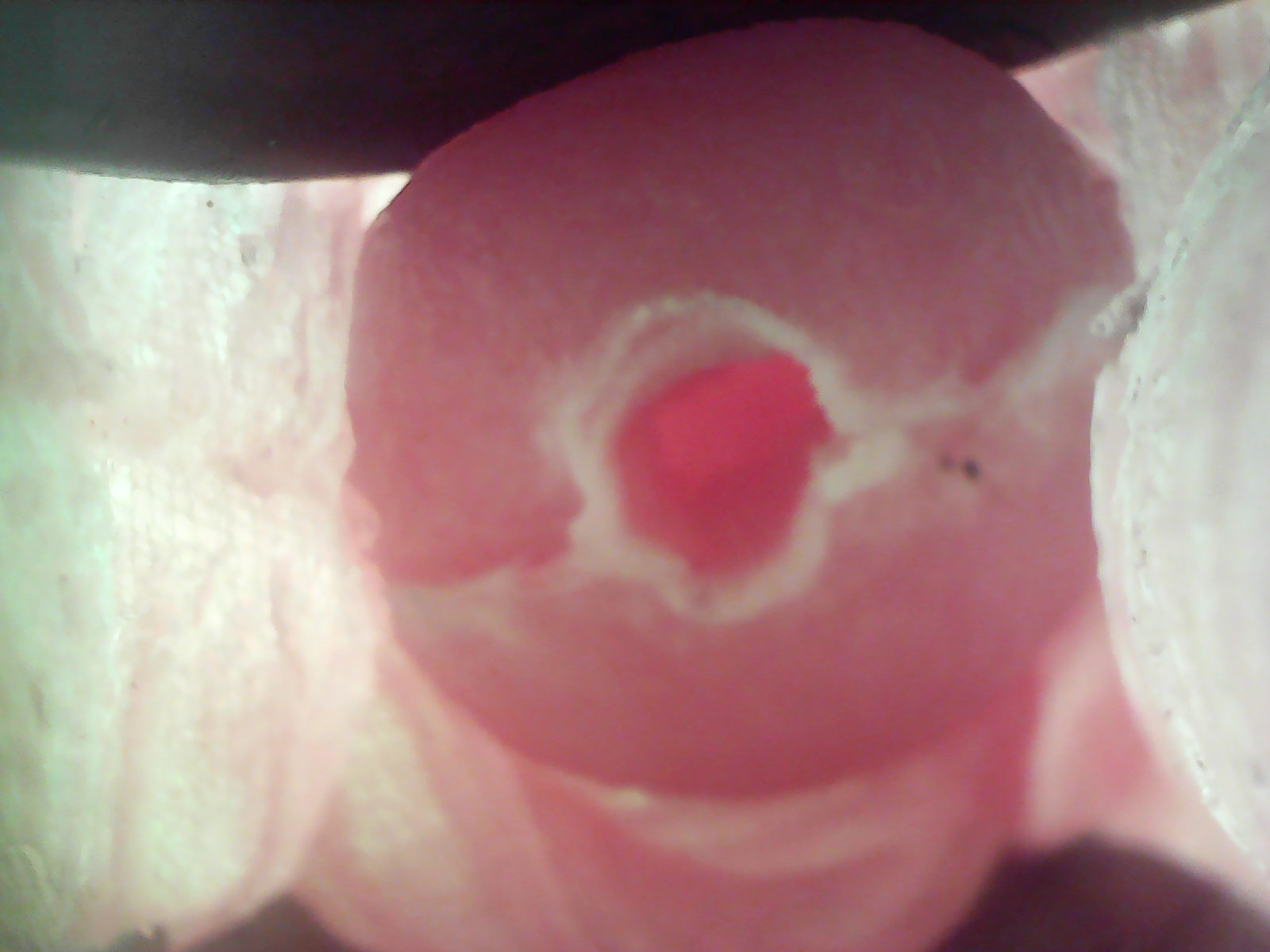

Supplement: S3 File — (ZIP) [file pone.0177782.s003.zip › S3 Fig 11. Graves speculum retroverted.tif]

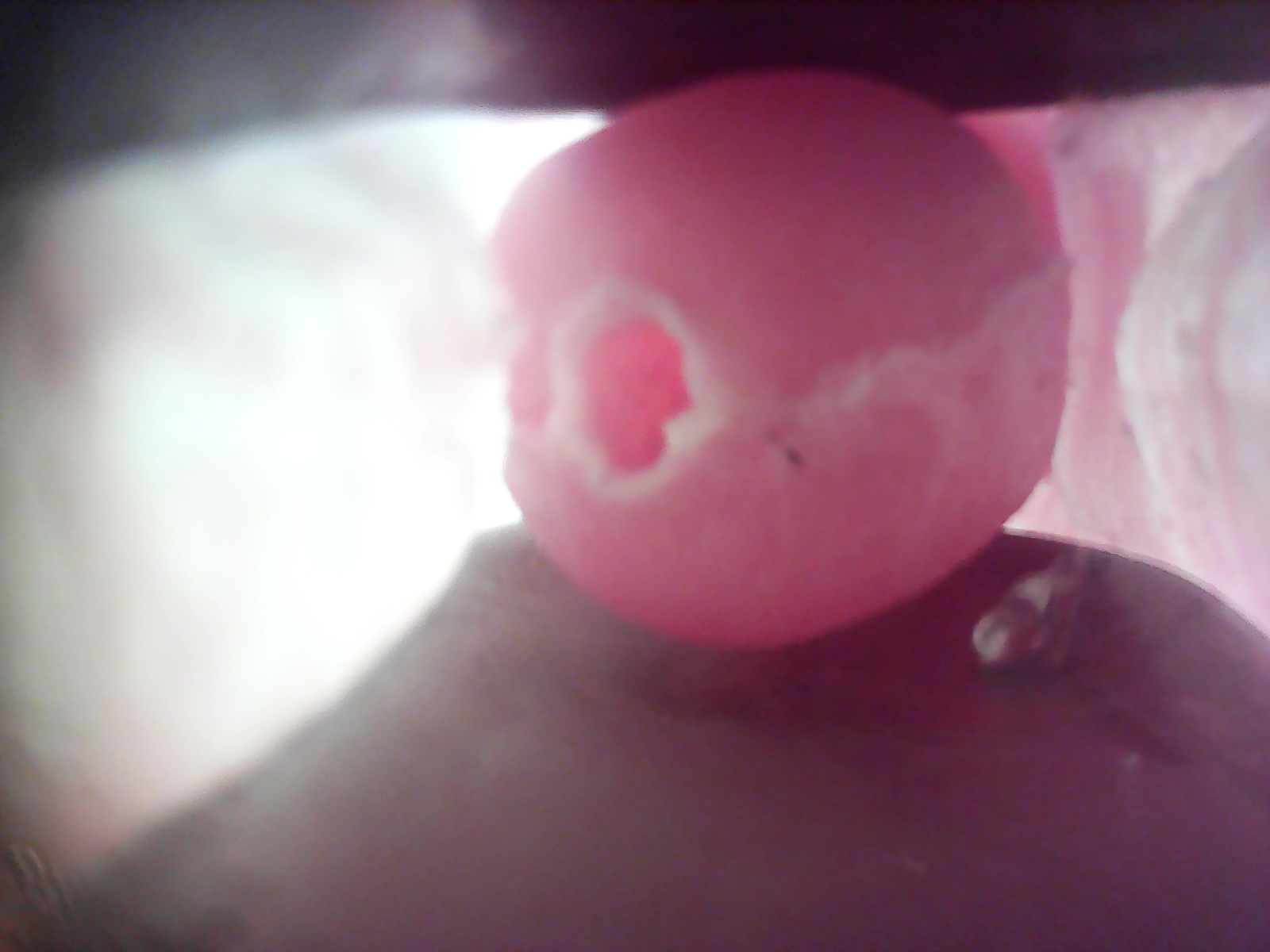

Supplement: S3 File — (ZIP) [file pone.0177782.s003.zip › S3 Fig 12. Graves speculum sideverted.tif]

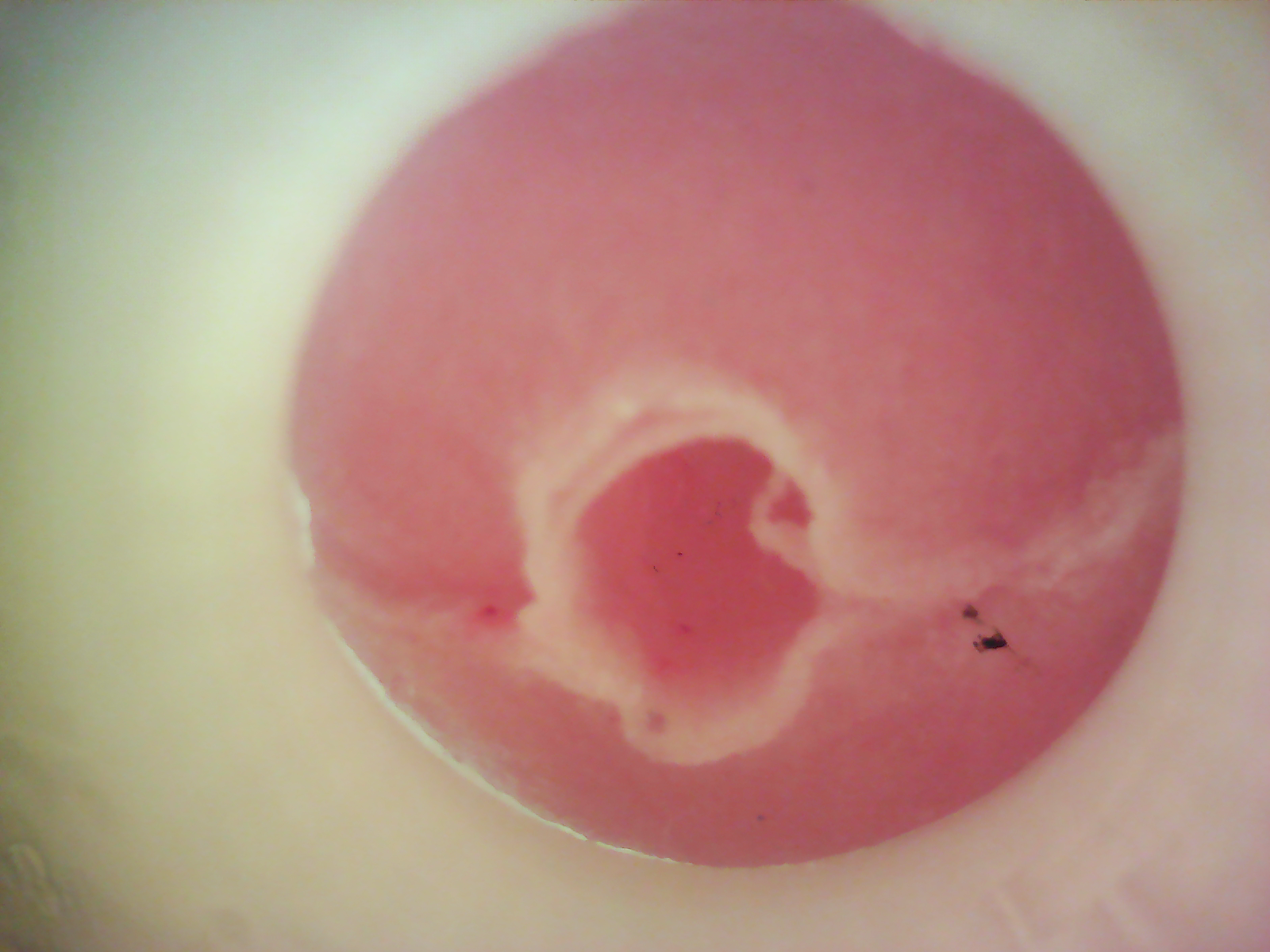

Supplement: S3 File — (ZIP) [file pone.0177782.s003.zip › S3 Fig 2. Curved-tip inserter mid-position.tif]

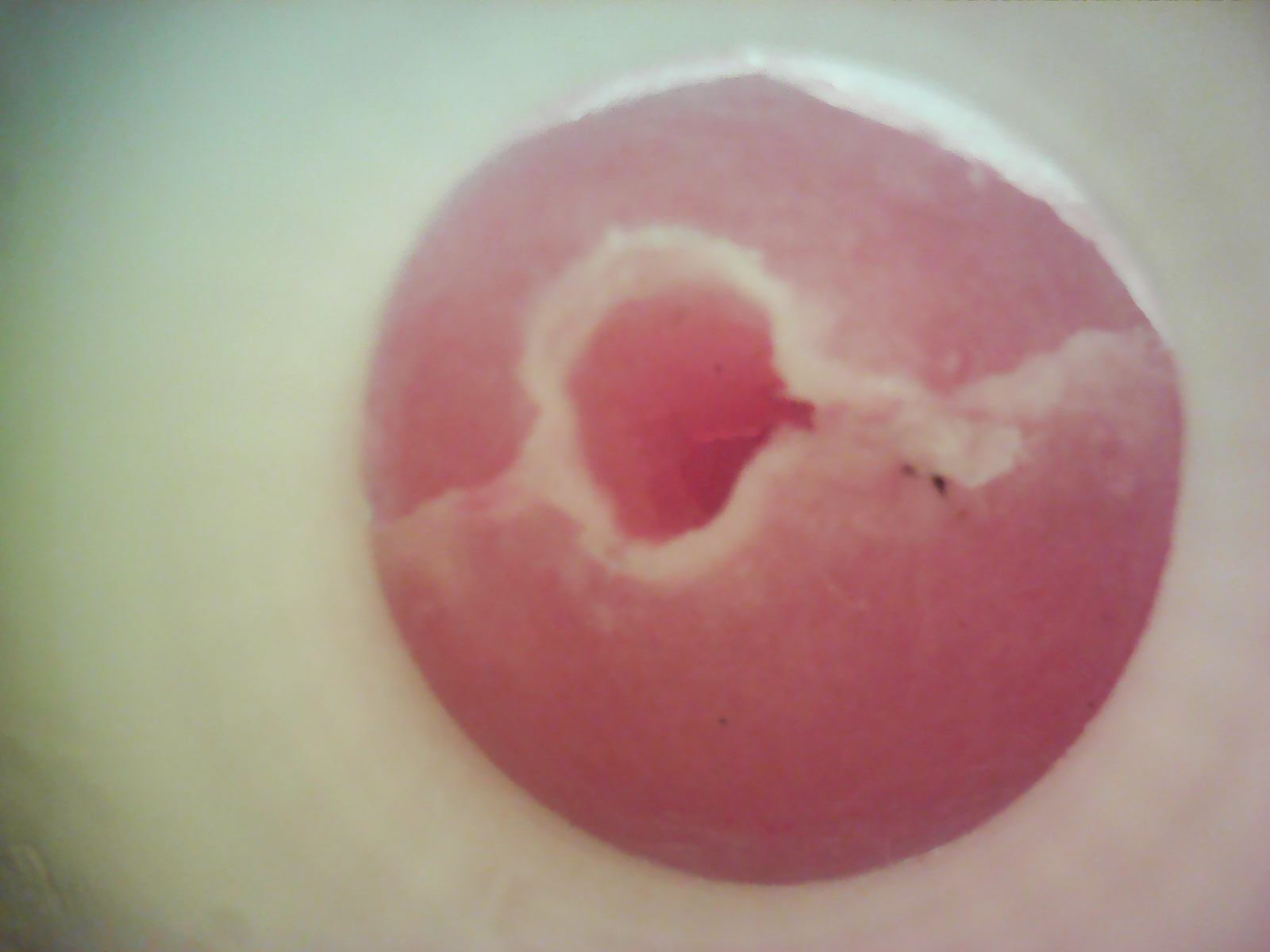

Supplement: S3 File — (ZIP) [file pone.0177782.s003.zip › S3 Fig 3. Curved-tip inserter retroverted.tif]

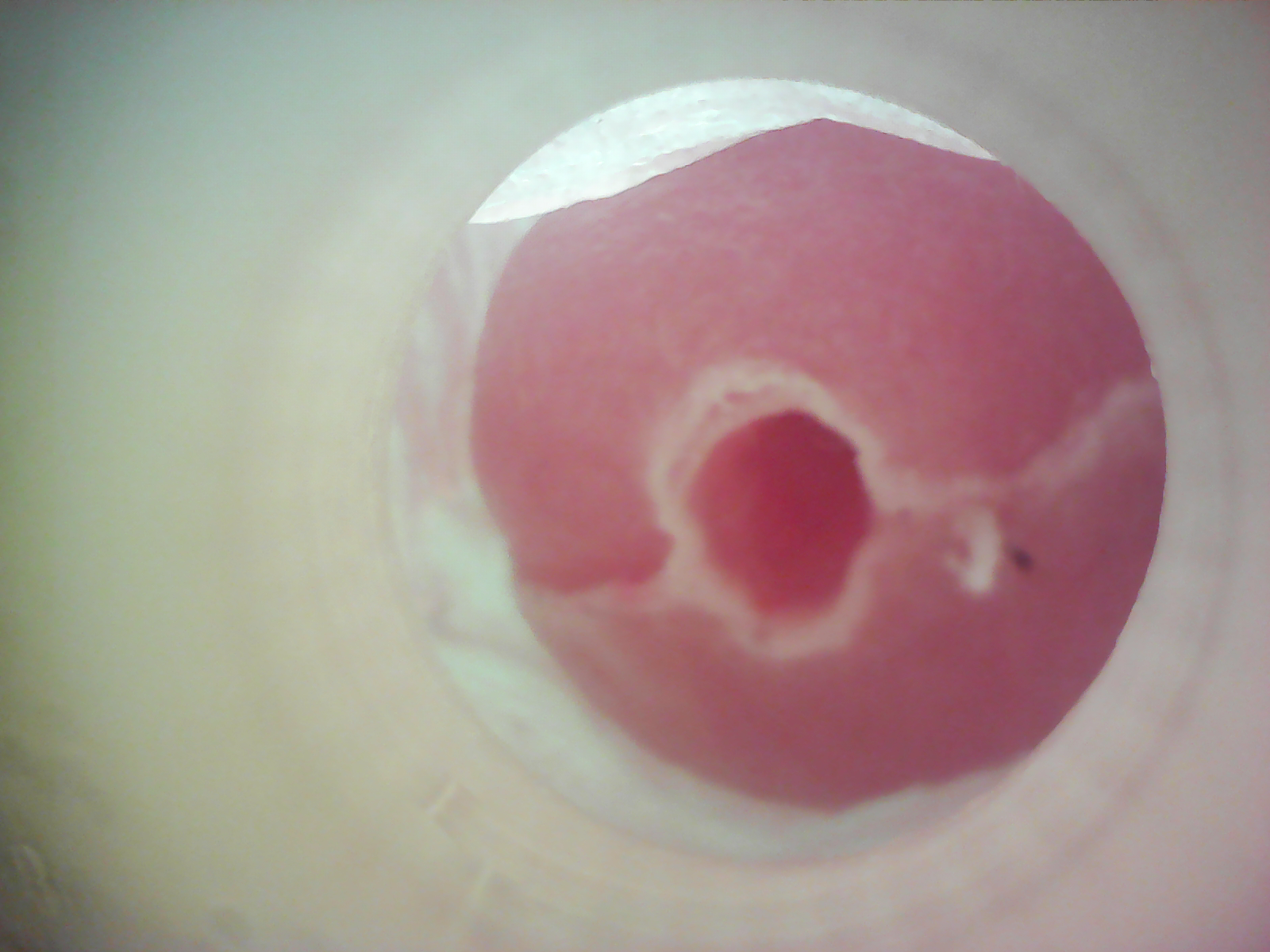

Supplement: S3 File — (ZIP) [file pone.0177782.s003.zip › S3 Fig 4. Curved-tip inserter sideverted.tif]

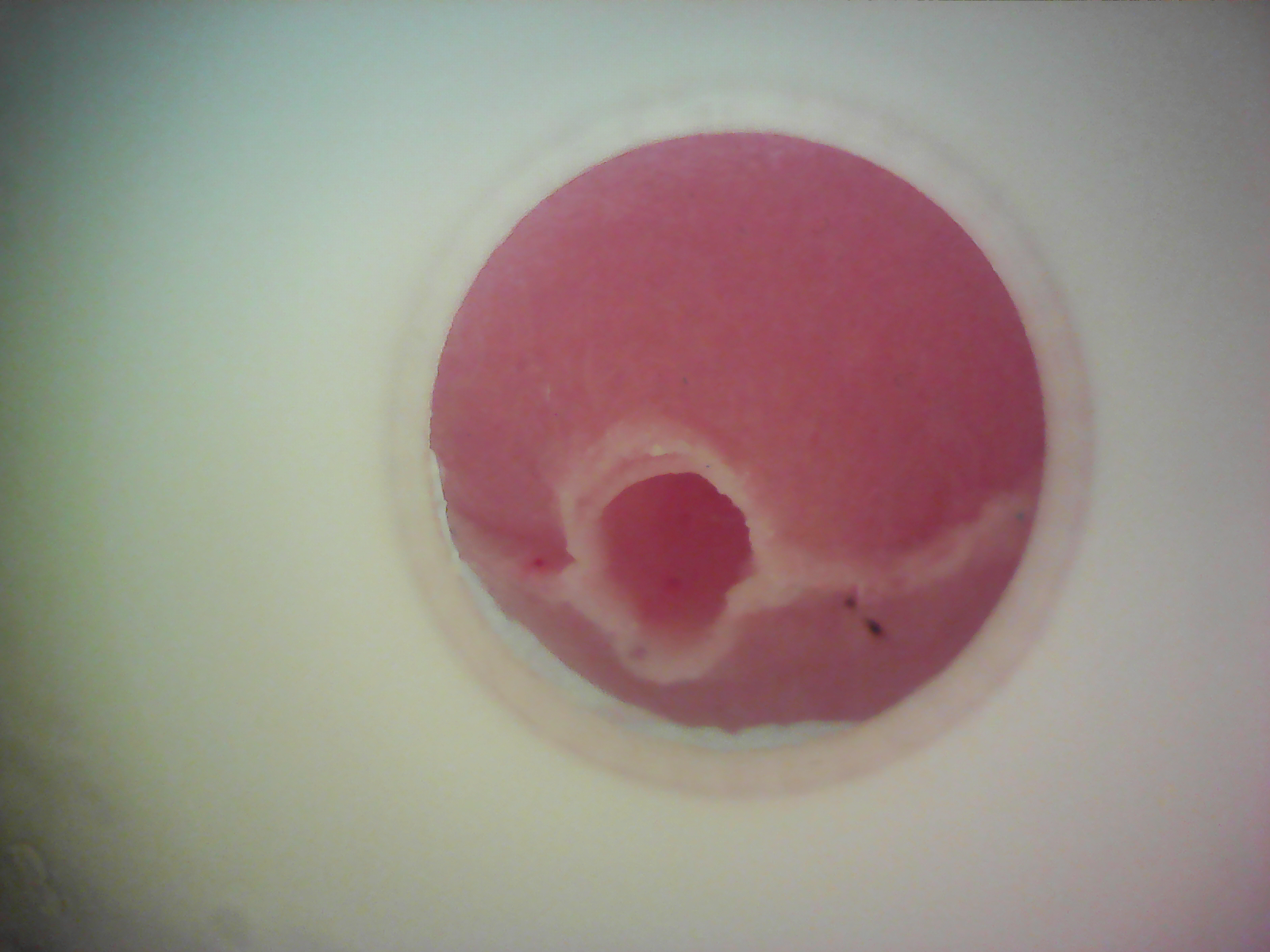

Supplement: S3 File — (ZIP) [file pone.0177782.s003.zip › S3 Fig 5. Flat-tip inserter anteverted.tif]

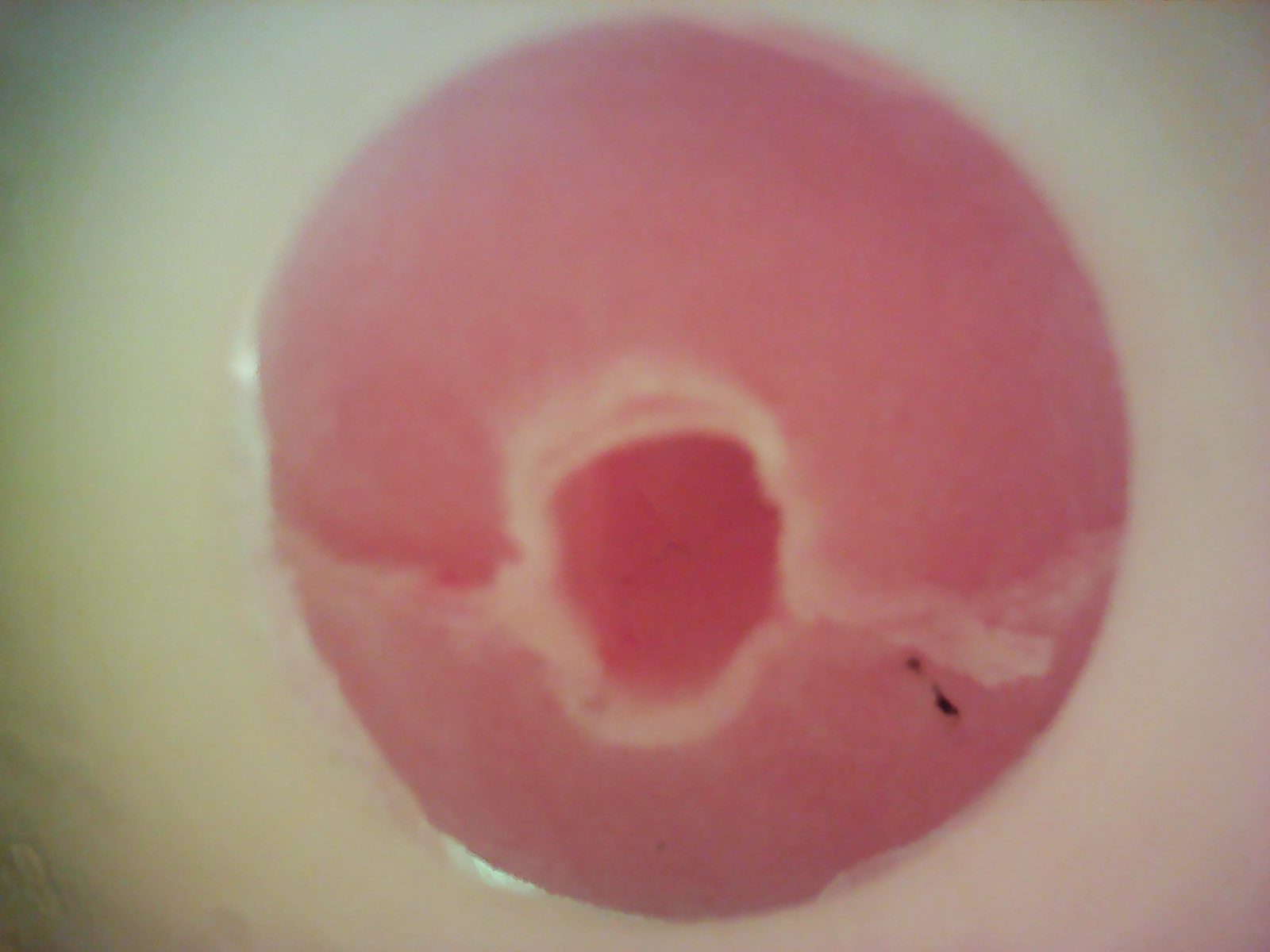

Supplement: S3 File — (ZIP) [file pone.0177782.s003.zip › S3 Fig 6. Flat-tip inserter mid-position.tif]

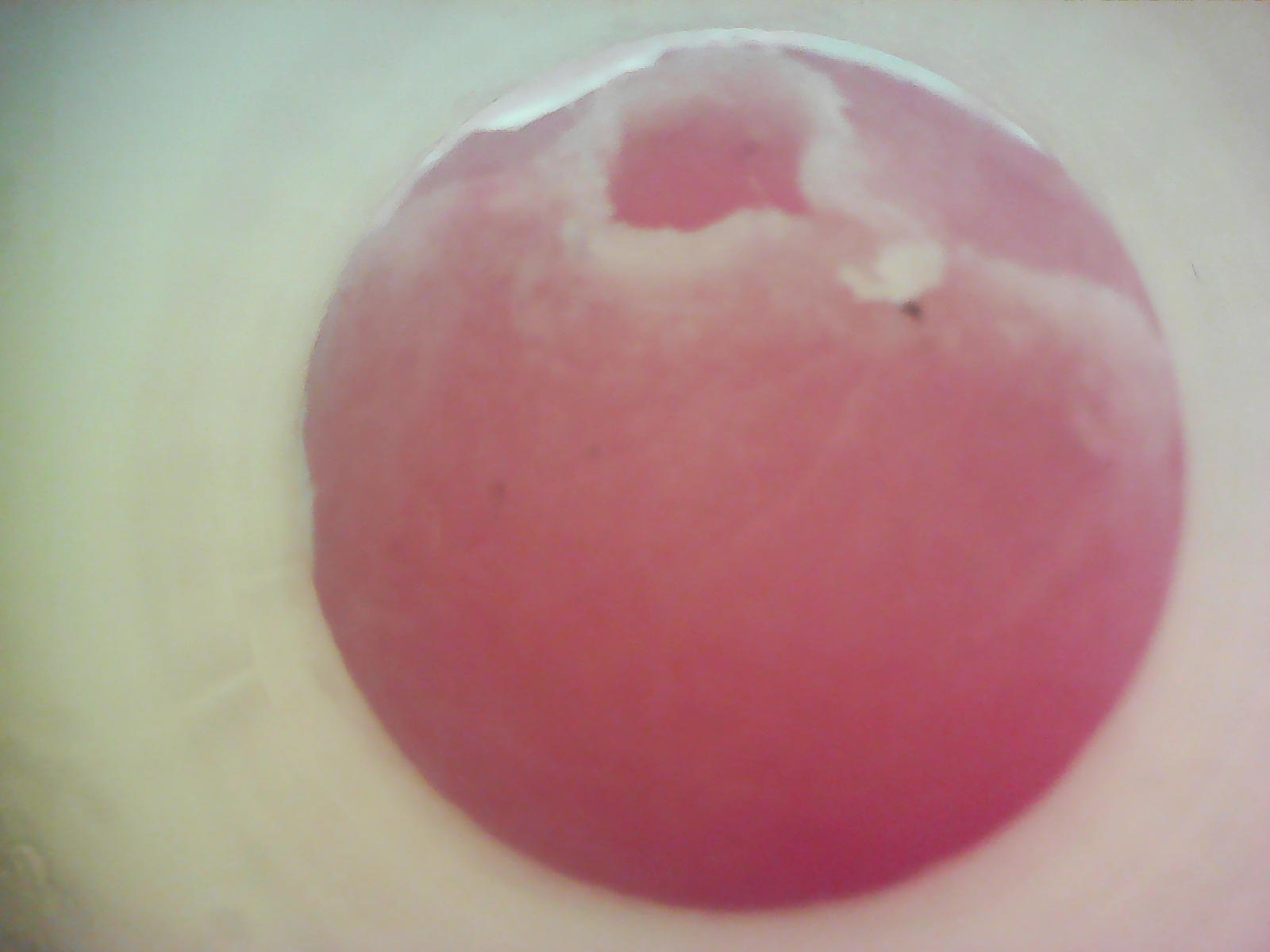

Supplement: S3 File — (ZIP) [file pone.0177782.s003.zip › S3 Fig 7. Flat-tip inserter retroverted.tif]

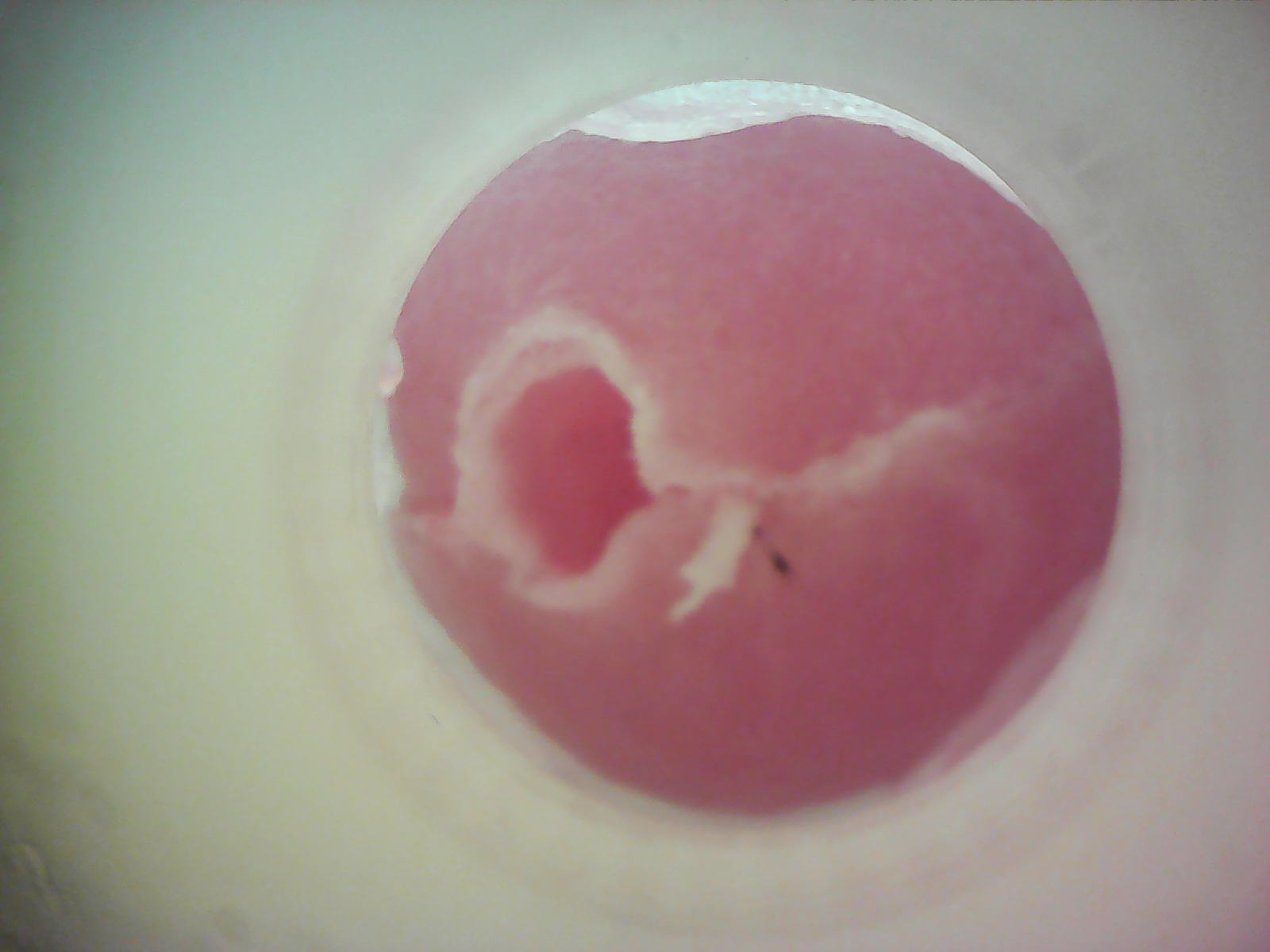

Supplement: S3 File — (ZIP) [file pone.0177782.s003.zip › S3 Fig 8. Flat-tip inserter sideverted.tif]

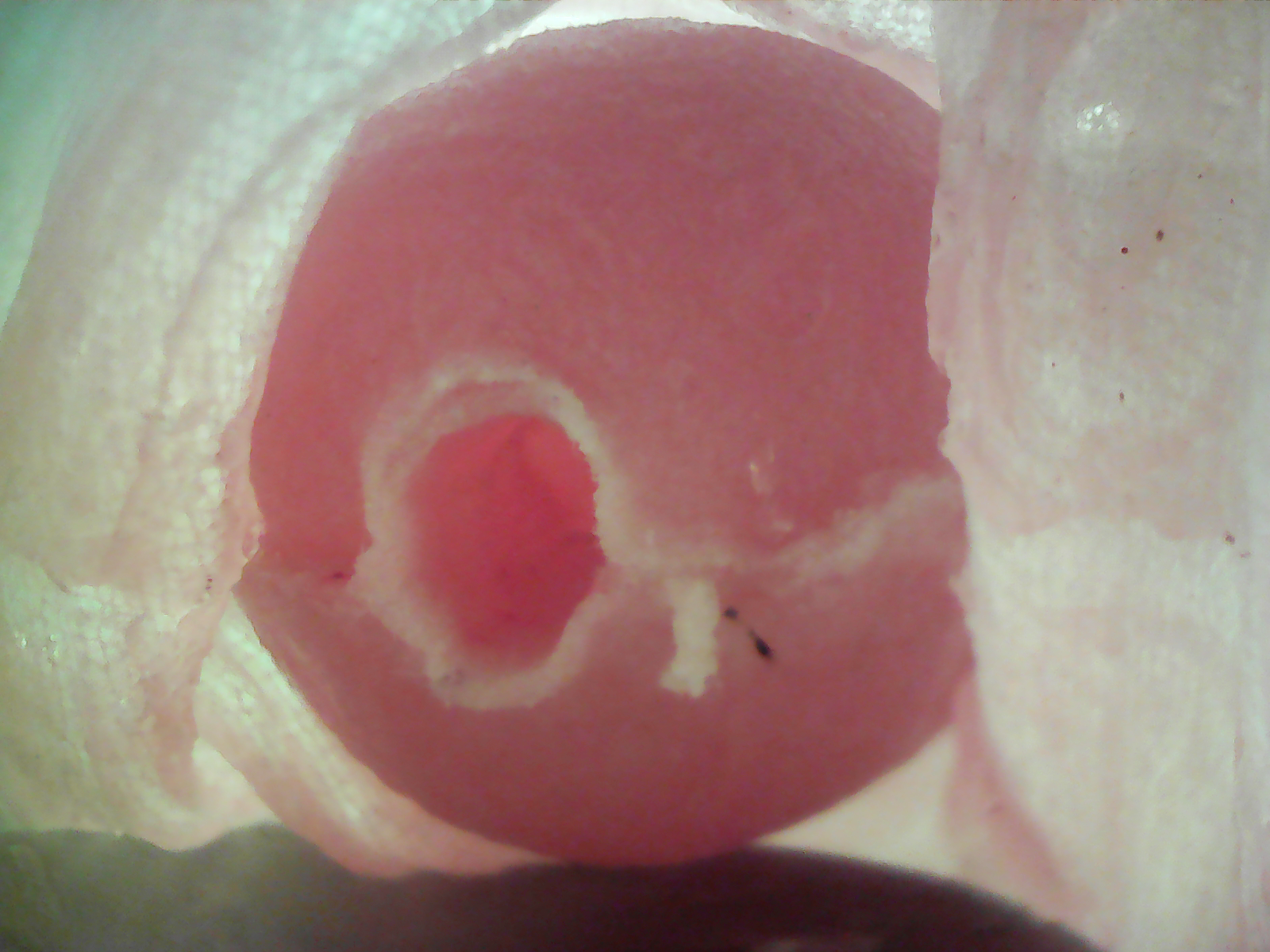

Supplement: S3 File — (ZIP) [file pone.0177782.s003.zip › S3 Fig 9. Graves speculum anteverted.tif]

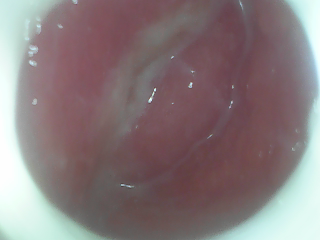

Supplement: S6 File — (ZIP) [file pone.0177782.s006.zip › S6 Fig 1.png]

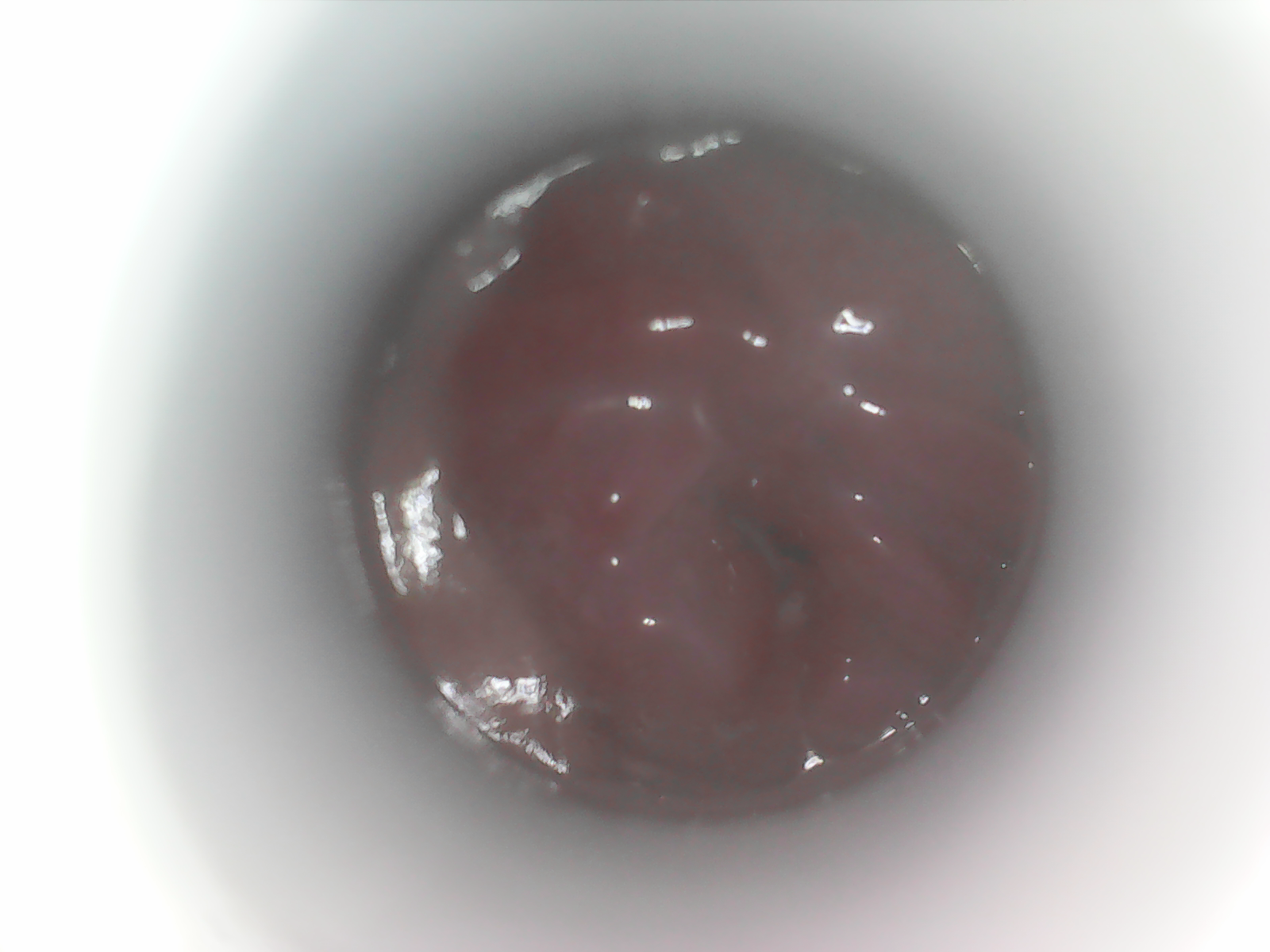

Supplement: S6 File — (ZIP) [file pone.0177782.s006.zip › S6 Fig 10.png]

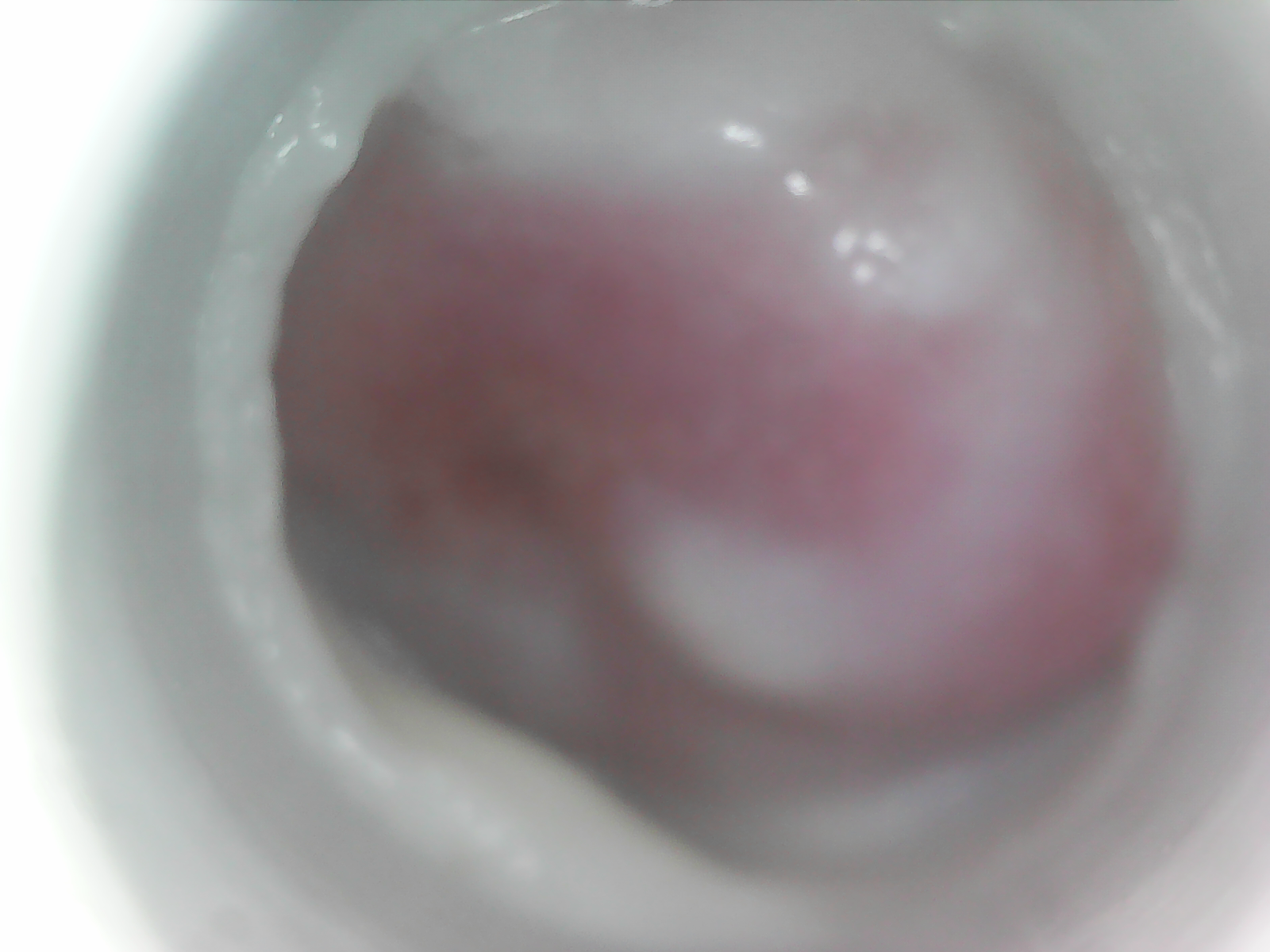

Supplement: S6 File — (ZIP) [file pone.0177782.s006.zip › S6 Fig 11.png]

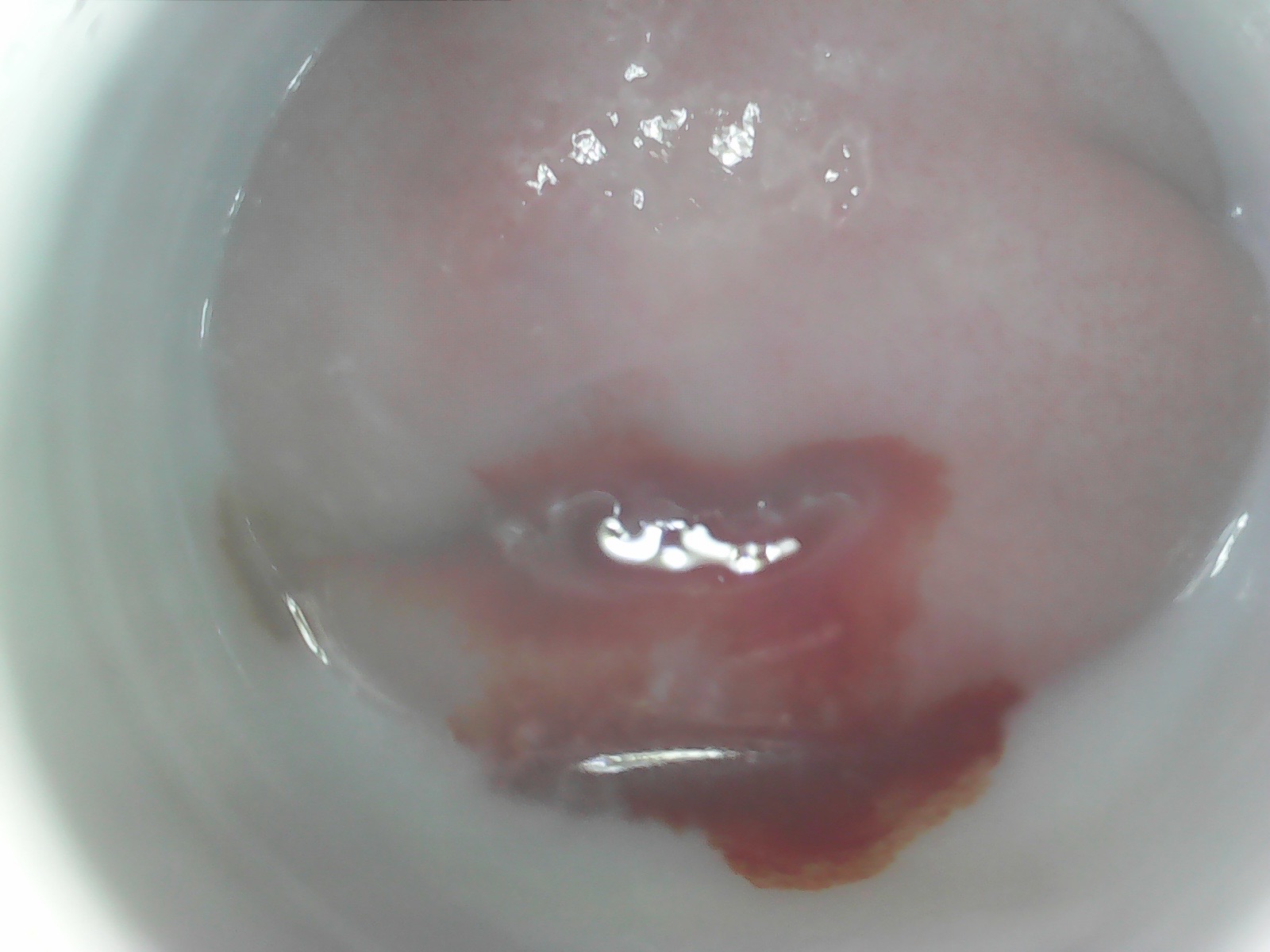

Supplement: S6 File — (ZIP) [file pone.0177782.s006.zip › S6 Fig 12.jpg]

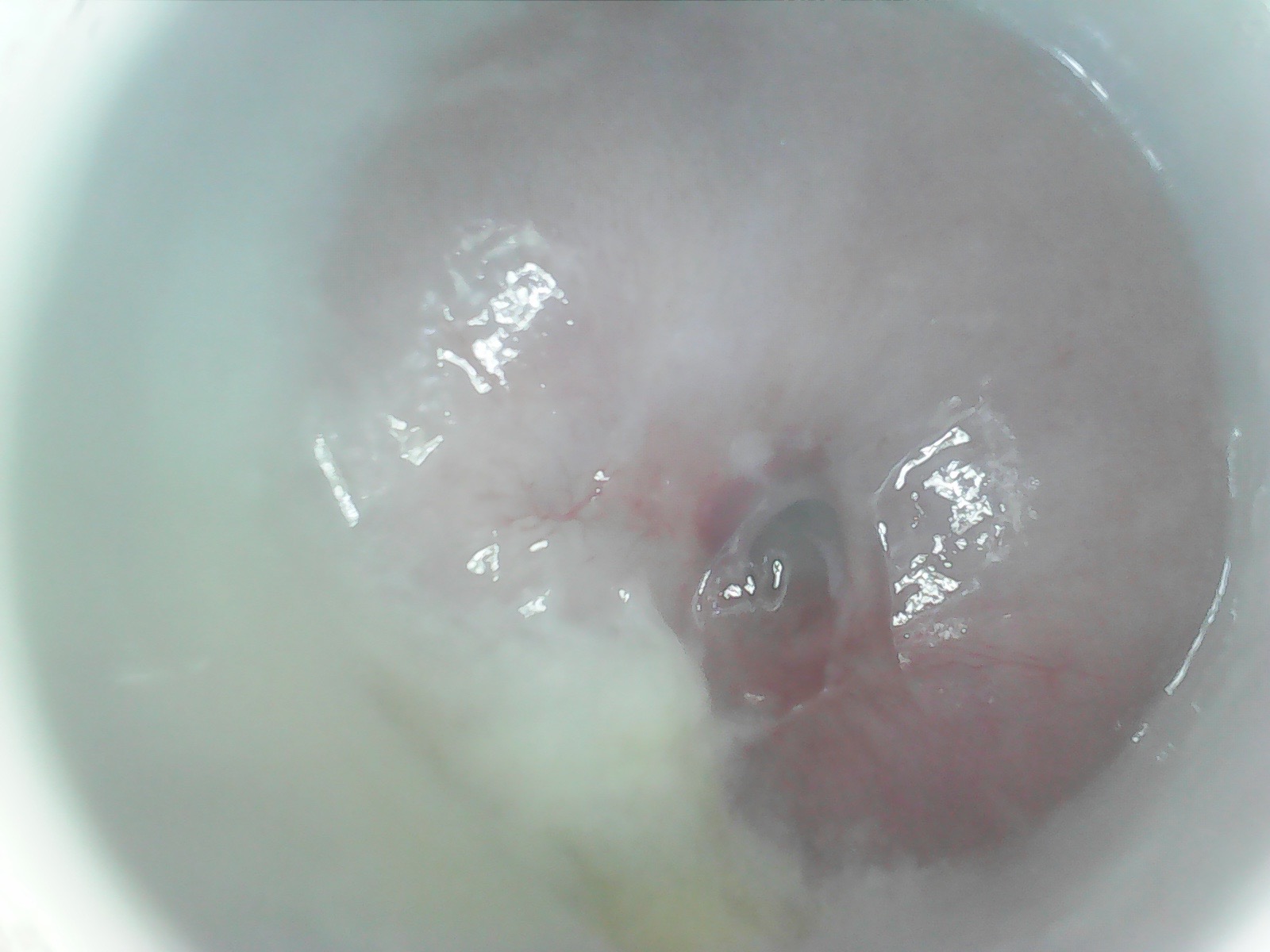

Supplement: S6 File — (ZIP) [file pone.0177782.s006.zip › S6 Fig 13.jpg]

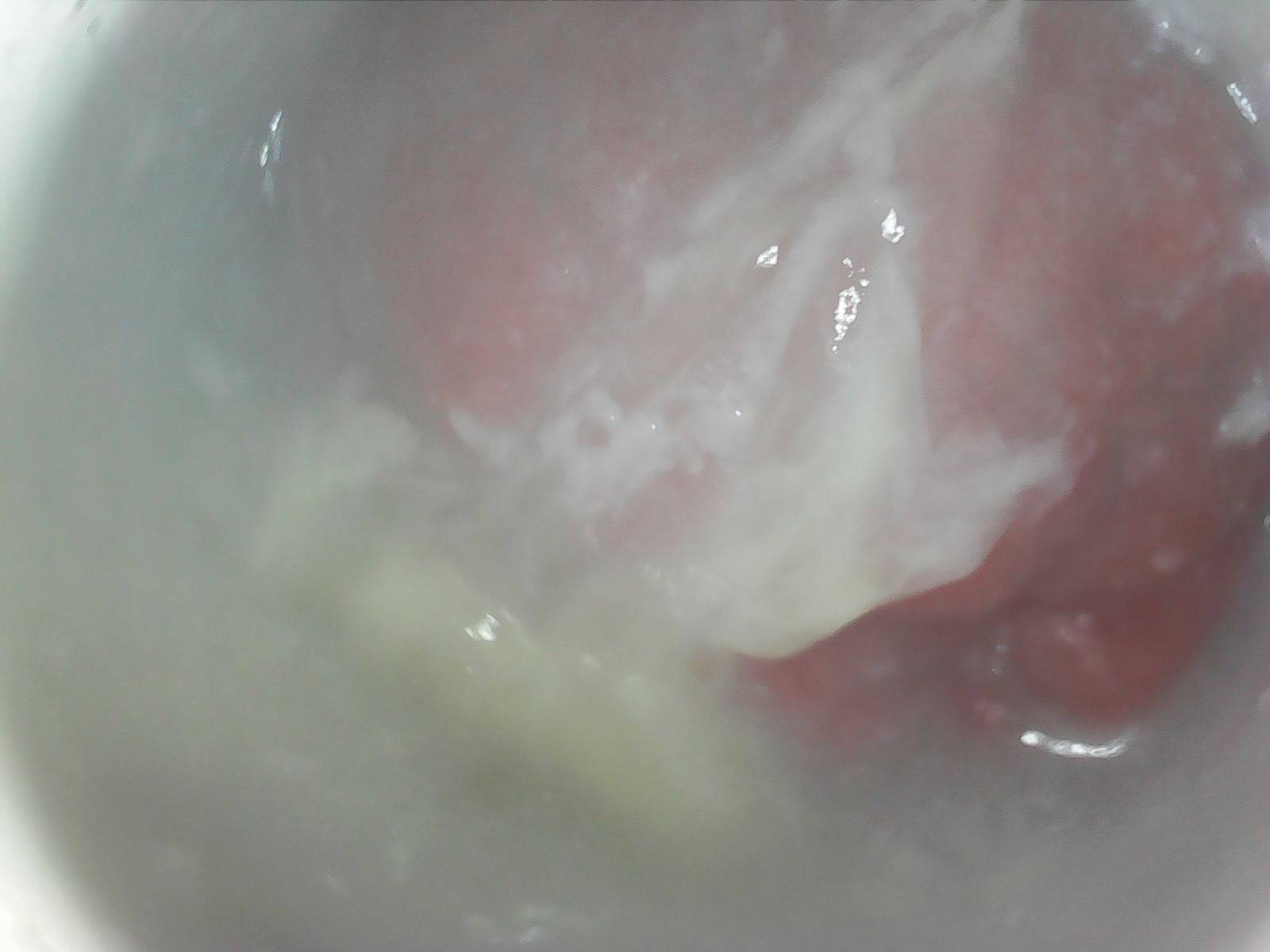

Supplement: S6 File — (ZIP) [file pone.0177782.s006.zip › S6 Fig 14.jpg]

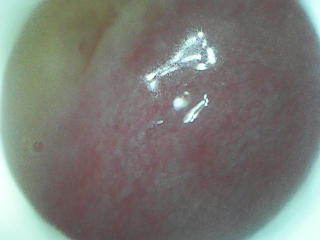

Supplement: S6 File — (ZIP) [file pone.0177782.s006.zip › S6 Fig 2.png]

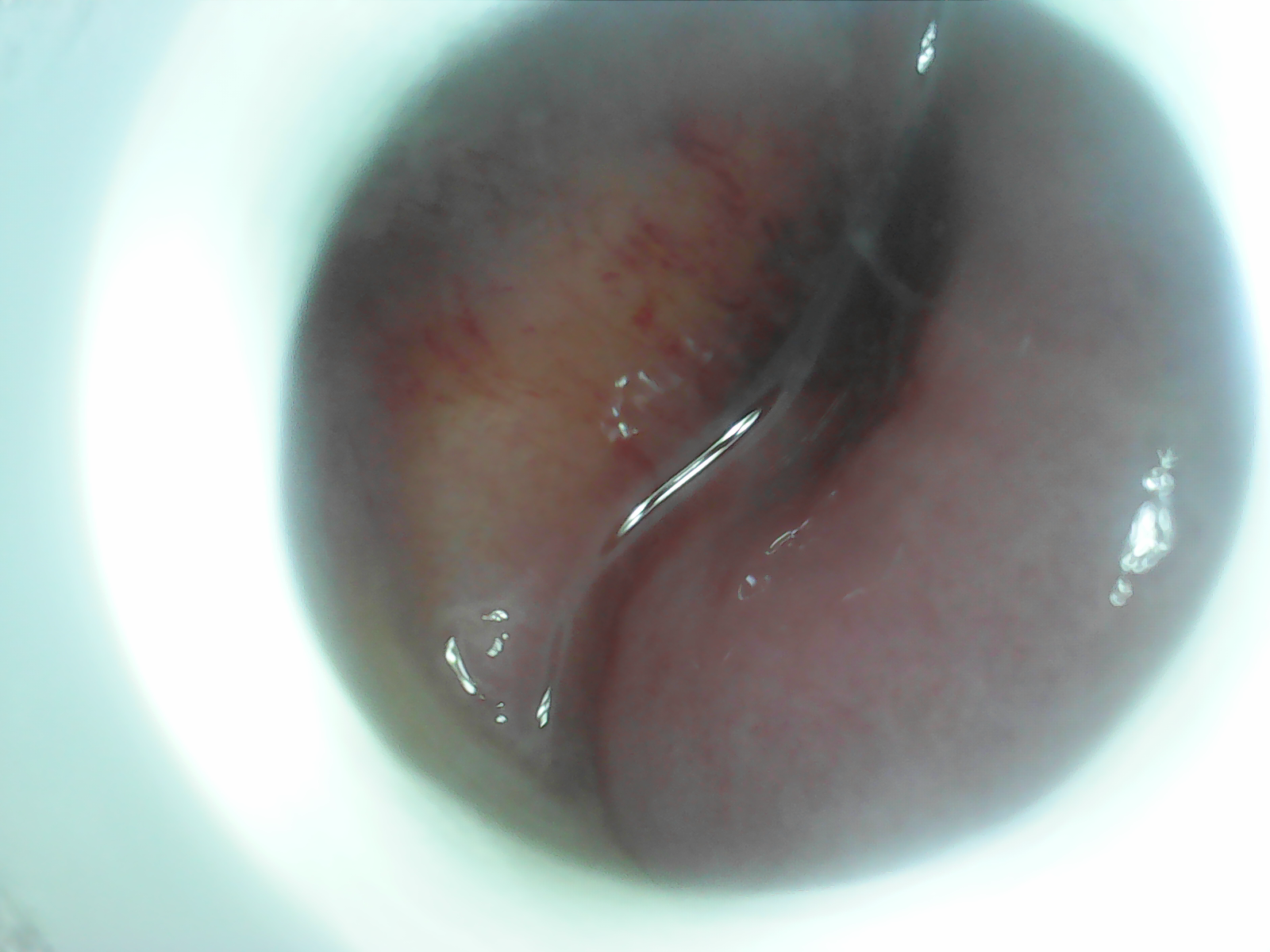

Supplement: S6 File — (ZIP) [file pone.0177782.s006.zip › S6 Fig 3.png]

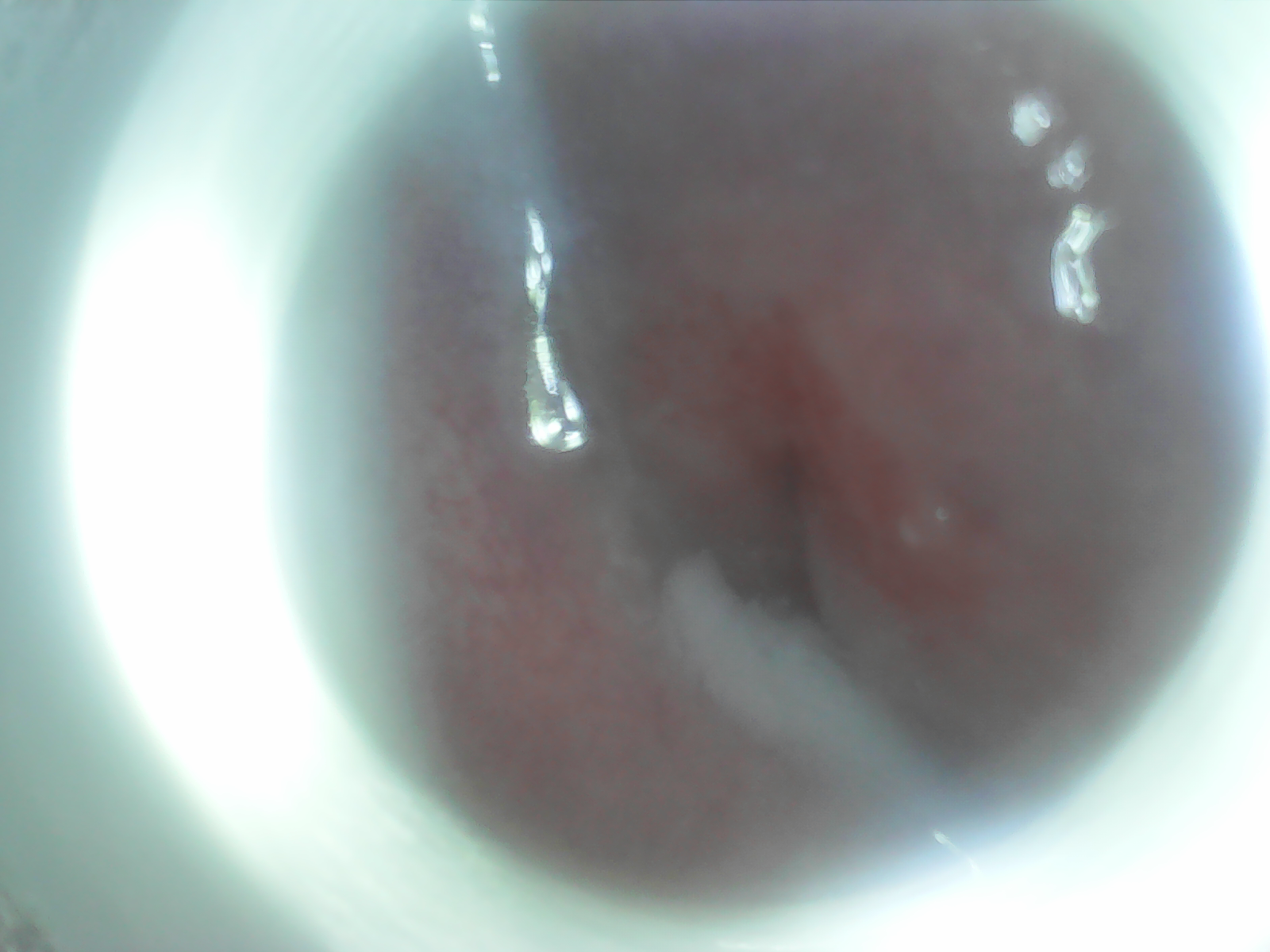

Supplement: S6 File — (ZIP) [file pone.0177782.s006.zip › S6 Fig 4.png]

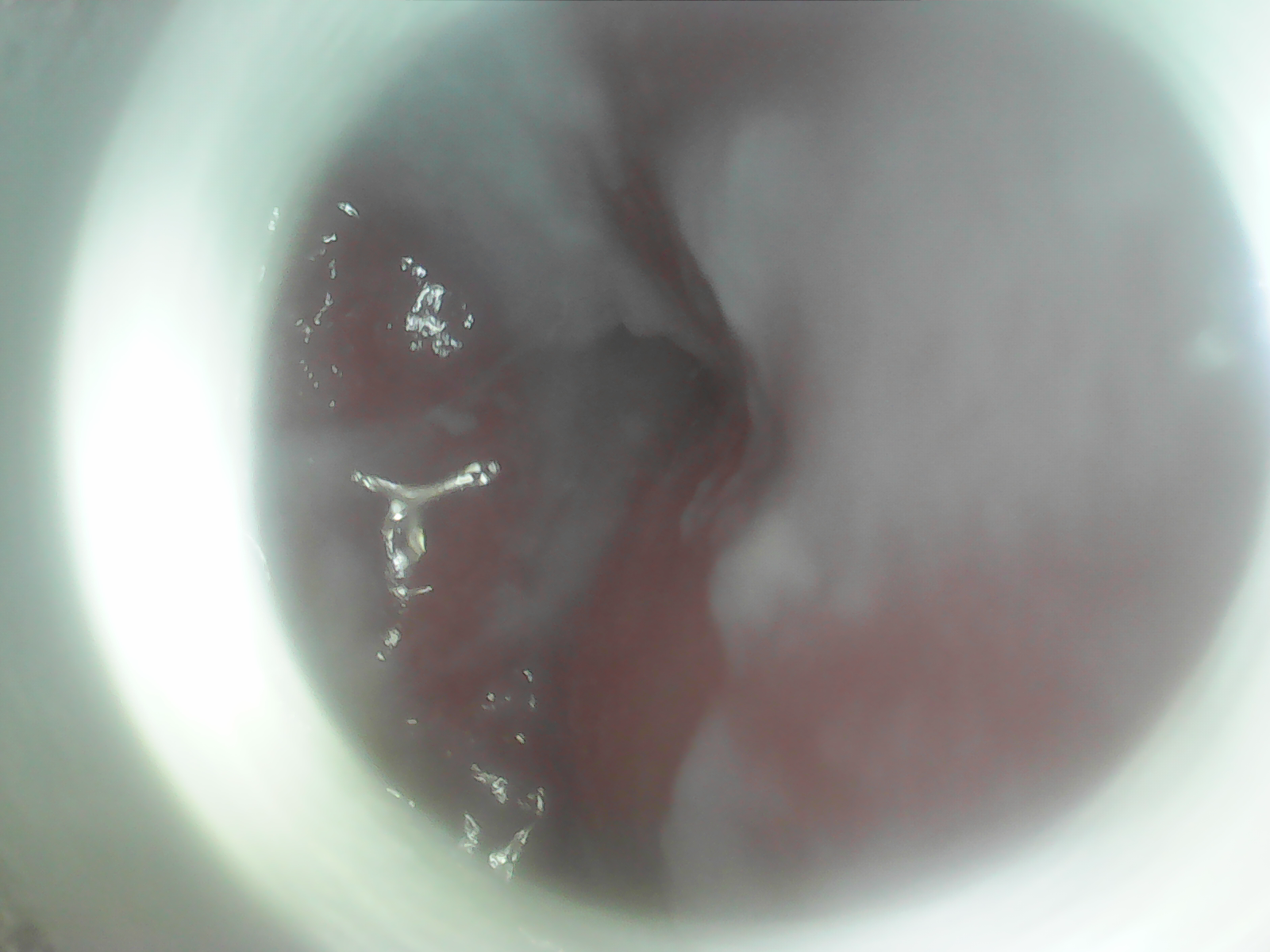

Supplement: S6 File — (ZIP) [file pone.0177782.s006.zip › S6 Fig 5.png]

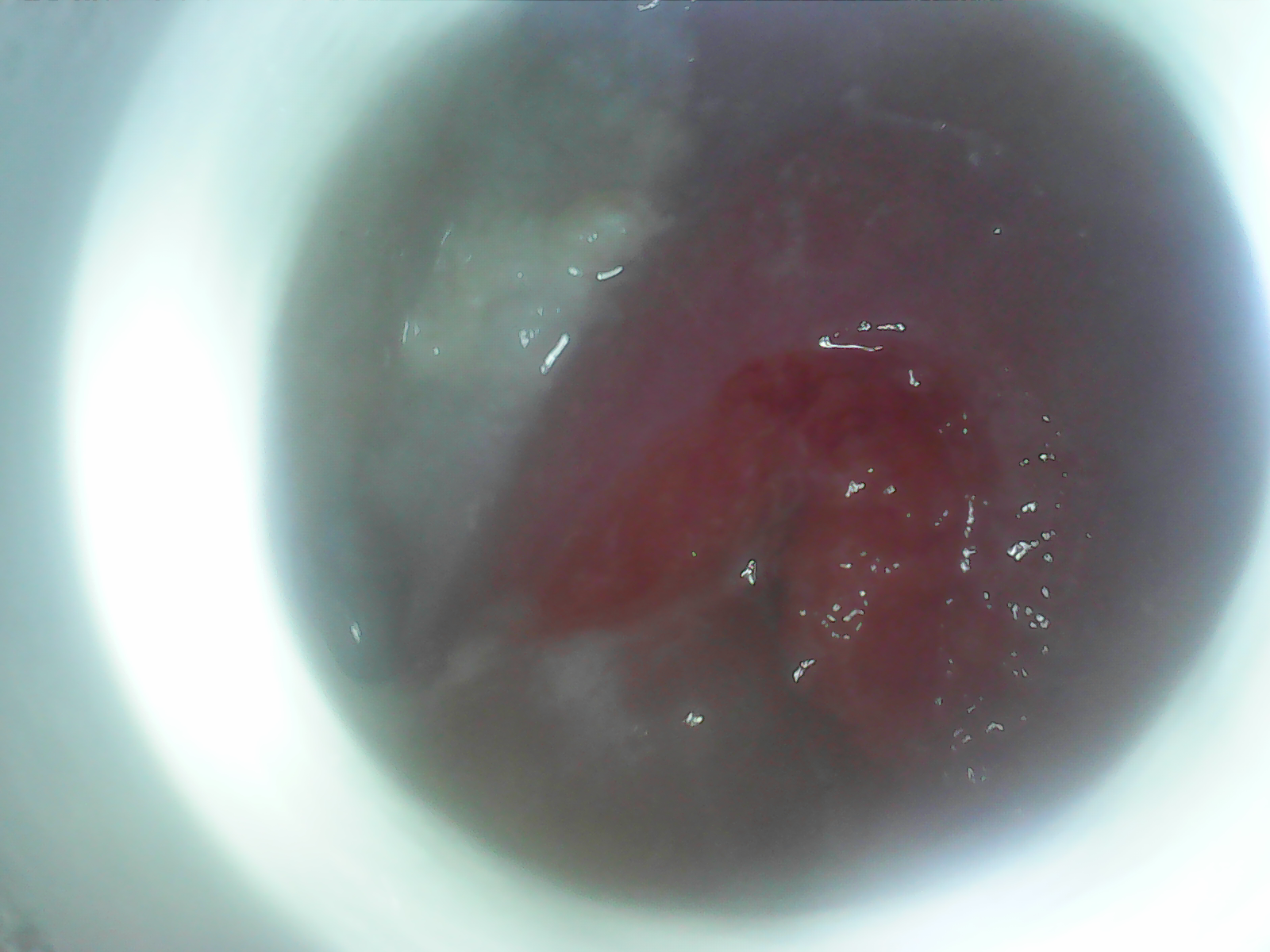

Supplement: S6 File — (ZIP) [file pone.0177782.s006.zip › S6 Fig 6.png]

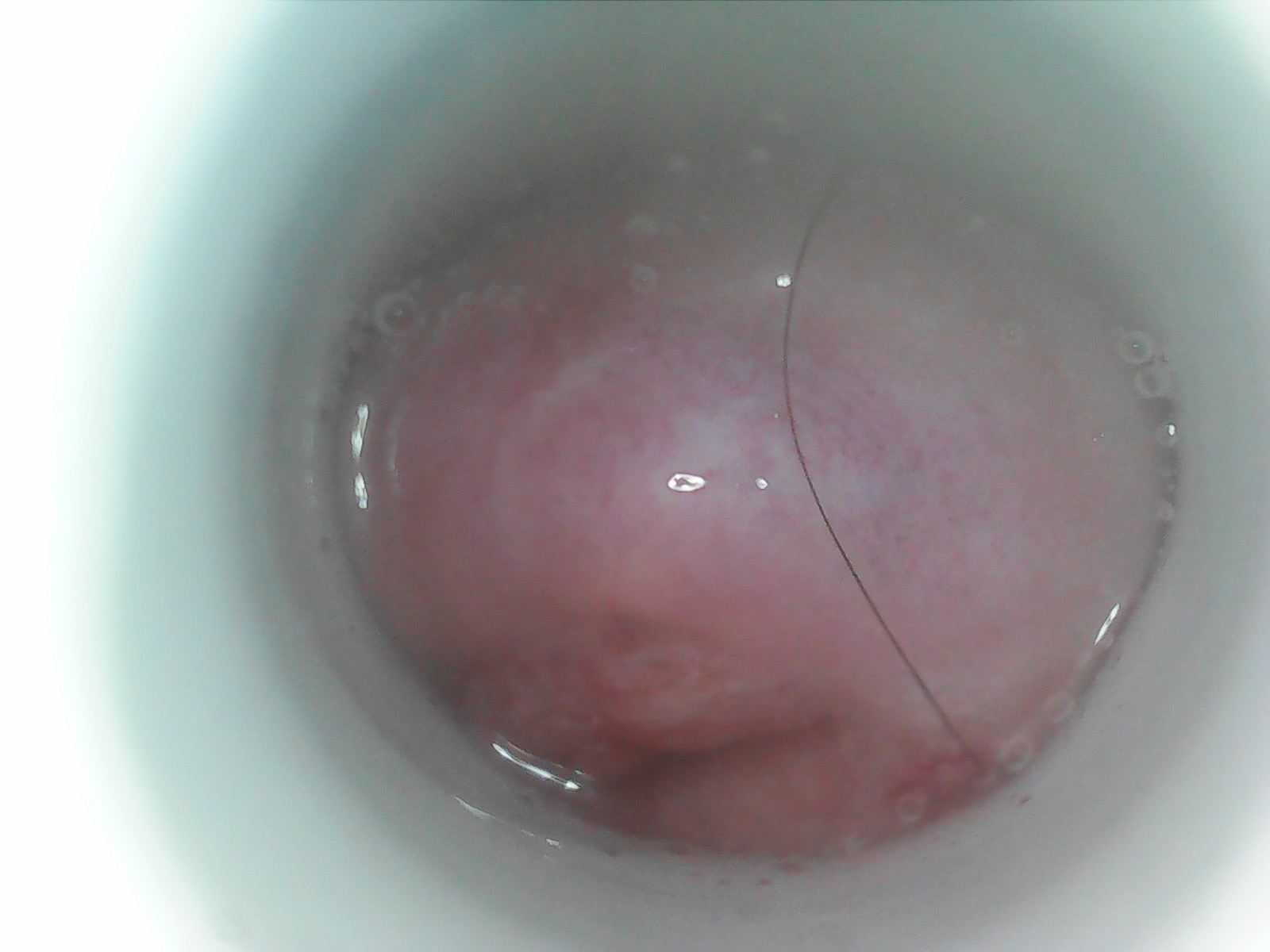

Supplement: S6 File — (ZIP) [file pone.0177782.s006.zip › S6 Fig 7.jpg]

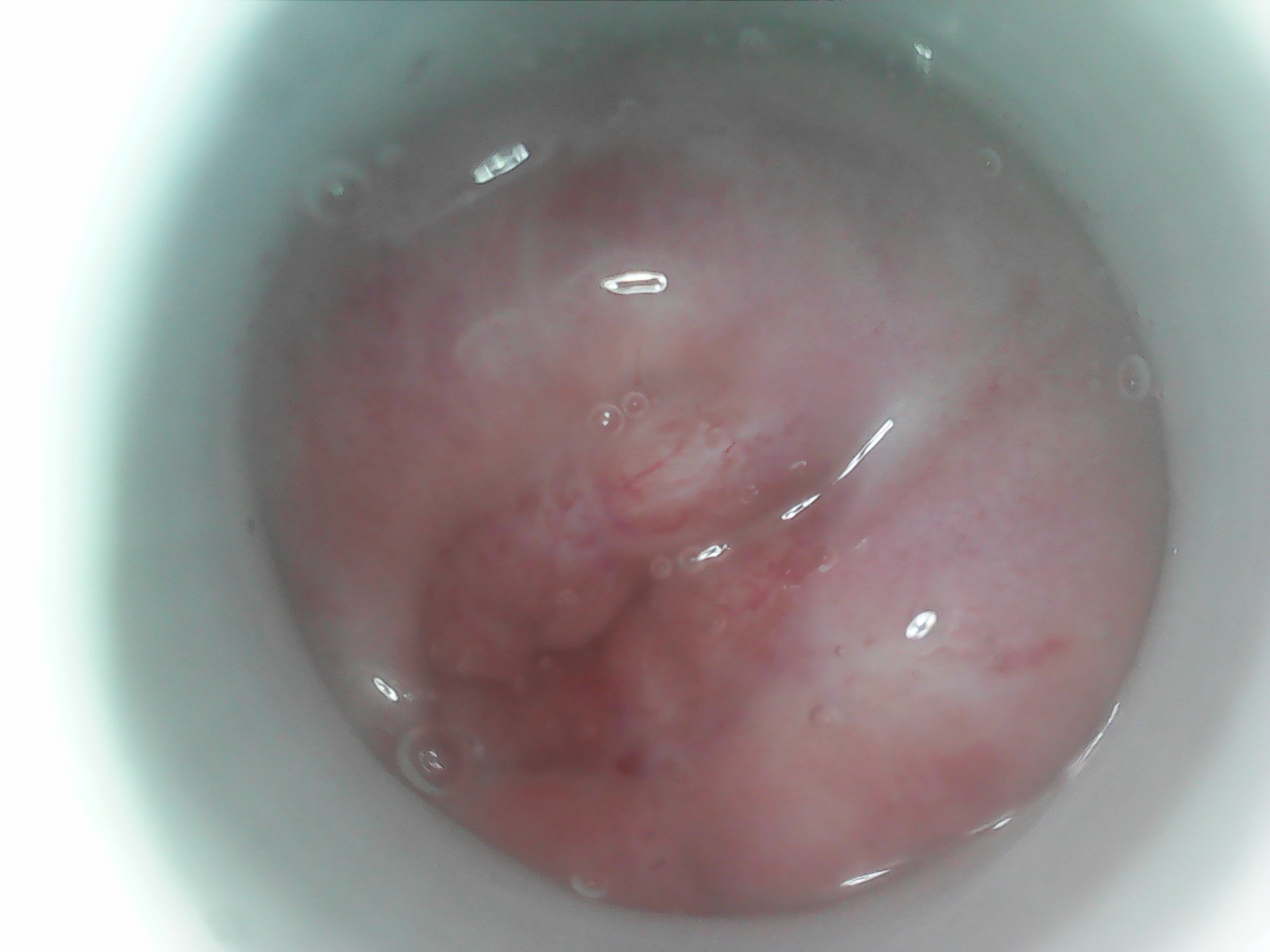

Supplement: S6 File — (ZIP) [file pone.0177782.s006.zip › S6 Fig 8.jpg]

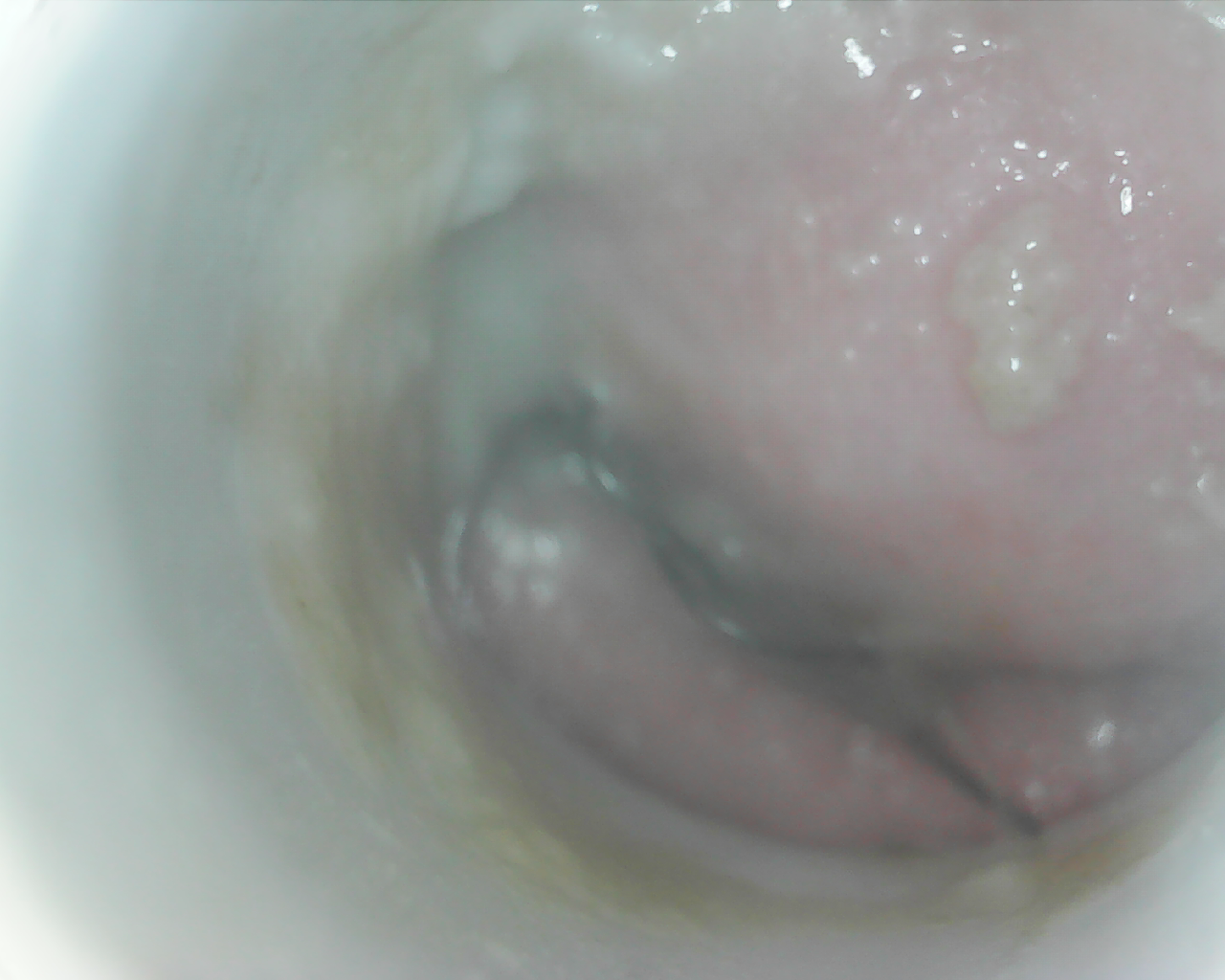

Supplement: S6 File — (ZIP) [file pone.0177782.s006.zip › S6 Fig 9.png]
