# Supplementary material for: Design and preliminary analysis of a vaginal inserter for speculum-free cervical cancer screening
Source: PLoS One. 2017 May 31;12(5):e0177782. doi: 10.1371/journal.pone.0177782 (PMC5451045; doi:10.1371/journal.pone.0177782)
Supplement: S4 File — (PDF) [file pone.0177782.s004.pdf]

Survey about perceptions regarding vaginal examinations

→ Survey to be completed **BEFORE** introduction of the Inserter

Date: _________________
Site: __________________
MRN:_________________
Patient ID:_____________
Patient Pre-examination questionnaire:

1. What is your age?

☐1 ≤29 ☐2 30-34 ☐3 35-39 ☐4 40-44 ☐5 45-49 ☐6 50-54
☐7 55-59 ☐8 60-64 ☐9 ≥65

2. How many times have you had a vaginal examination performed with a speculum?
☐1 Never ☐2 1-2 ☐3 3-5 ☐4 6-10 ☐5 More than 10

3. Are you a regular user of Tampons or Menstrual cups? Yes/ No

4. How vaginal births have you had? ____

5. How much of a barrier, if any, do you consider speculums to be to you getting screened for cervical cancer?
1- not a barrier 2- small barrier 3- medium barrier 4-large barrier

We would like to know how acceptable or unacceptable you find various approaches and tools for screening for cervical cancer.

Check if no previous experience with the speculum: ☐

**How willing are you to be screened for cervical cancer using:**	**Not at all willing**	**Slightly willing**	**Very willing**	**Extremely willing**
a. (From previous experience) The vaginal speculum. **Skip this question if no previous experience with the speculum.**	1☐	2☐	3☐	4☐
b. (Based of appearance only) the vaginal speculum	1☐	2☐	3☐	4☐
c. (Based off appearance only), the inserter	1☐	2☐	3☐	4☐
d. Physician examination with the speculum	1☐	2☐	3☐	4☐
e. Self-insertion with the speculum	1☐	2☐	3☐	4☐
f. Physician examination with the inserter	1☐	2☐	3☐	4☐
g. Self-examination with the inserter	1☐	2☐	3☐	4☐

We would like to hear about your preferences in cervical cancer screening.

**6. Select 3 for each question**	**Cost**	**Procedure time**	**Adequate assessment of cancer risk**	**Physician gender**	**How comfortable the screening is**	**How long it takes to get to the clinic**
a. In your opinion, what are the 3 **most important** features for your cervical cancer screening?	1☐	2☐	3☐	4☐	5☐	6☐

7. Do you have any additional comments or feedback on the Inserter?

____
____

Survey about Perceptions regarding vaginal examinations
→ Survey to be completed **AFTER** introduction of the Expander

PLEASE READ: Pay attention to whether you have any discomfort when you put the inserter in, when you take it out, and when you move it around to get a good picture of the cervix. It would also be helpful if you would pay attention to how easy or difficult using the inserter is.

Date: __________________
Site: ___________________

8. When you put the inserter in, how much discomfort did you feel, if any?

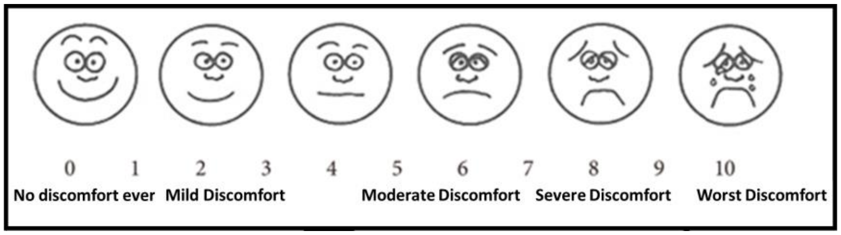

9. When you moved the inserter around to take a picture of your cervix, how much discomfort did you feel, if any?

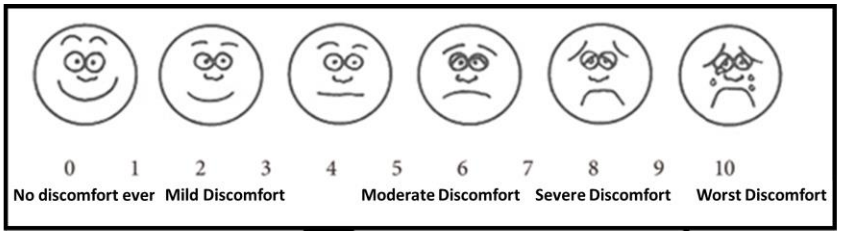

10. When you took the inserter out, how much discomfort did you feel, if any?

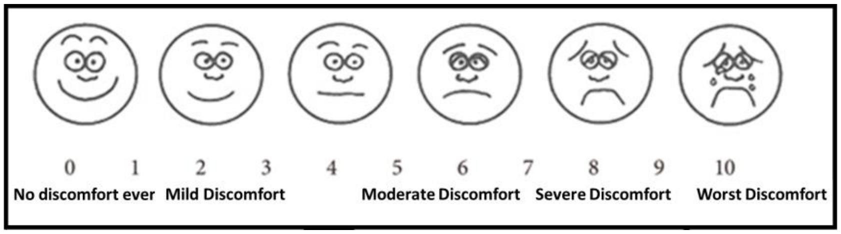

We would like to hear more about your experience with the inserter.

11. **Comparability Questions**<br>Check if no previous experience with the speculum: ☐<br>**After having completed a cervical cancer screening using the inserter:**	**Much worse**	**Slightly worse**	**Slightly better**	**Much better**
a. How did this experience compare to previous vaginal examinations you have had? **Skip this question if no previous vaginal examinations.**	1☐	2☐	3☐	4☐
b. In terms of **comfort**, how did the exam with the inserter compare to an exam with a speculum?	1☐	2☐	3☐	4☐
c. In terms of **ease,** how did examination with inserter compare to examination with speculum?	1☐	2☐	3☐	4☐
d. Would you say overall that the inserter is better or worse than the speculum?	1☐	2☐	3☐	4☐

12. We would love to hear any feedback you have on the inserter that might help us make it better. Please share any thoughts you have.

____

____

Survey about perceptions regarding vaginal examinations
→ Survey to be completed **BEFORE** introduction of the Inserter

Clinician Pre examination Questionnaire

Date: _________________
Site: __________________

First, please help us understand more about your experiences in healthcare.

1. **What is your current medical position?**
   ☐1 General Physician ☐2 Nurse ☐3 Physician Assistant
   ☐4 Ob-Gyn ☐5 Other_____________
2. **How many years have you been working in health care delivery?** ________
3. **How many years have you been providing cervical cancer screening?**_________
4. **Are you currently a resident or trainee?** ☐1 Yes ☐2 No
5. **Are you male or female?** ☐1 Female ☐2 Male
6. **What is your age?** ☐1 ≤29 ☐2 30-34 ☐3 35-39 ☐4 40-44 ☐5 45-49
   ☐6 50-54 ☐7 55-59 ☐8 60-64 ☐9 ≥65
7. **Where do you practice?** ***Select all that apply.***
   ☐1 Public hospital ☐2 Private office ☐3 Private hospital ☐4 Other *(please name)*: __________

We would like to hear about your preferences in cervical cancer screening.

8. **Select 3 for each question**	**Cost to the patient**	**Procedure time**	**Cost of the device**	**Training requirements**	**Maintenance costs**	**Ease of use**	**Physician comfort**	**Patient comfort**	**Quality of visualization of cervix**	**Device size**
a. In your opinion, what are the 3 **most important** features for cervical cancer screening devices?	1☐	2☐	3☐	4☐	5☐	6☐	7☐	8☐	9☐	10☐
b. In your opinion, what are the 3 **least important** features for cervical cancer screening devices?	1☐	2☐	3☐	4☐	5☐	6☐	7☐	8☐	9☐	10☐

This next section asks you about your experiences with the vaginal speculum.

9. **Do you use a speculum in your current screening procedures?** ☐1 **Yes** ☐2 **No** **(If "no," skip to question 10)**	**Metal Cusco**	**Plastic Cusco**	**Metal Graves**	**Plastic Graves**	**Metal Pederson**	**Plastic Pederson**
a. Which speculum types have you had experience with?	1☐	2☐	3☐	4☐	5☐	6☐
b. Which types do you currently use	1☐	2☐	3☐	4☐	5☐	6☐

10. **According to your patients, how much of a barrier to cervical cancer screening is each of the following?**	**Not a barrier**	**Small barrier**	**Medium barrier**	**Large barrier**
a. Male medical professional performing the exam?	1☐	2☐	3☐	4☐
b. Fear of pain from the speculum?	1☐	2☐	3☐	4☐
c. Access to a healthcare facility with cervical cancer screening?	1☐	2☐	3☐	4☐
d. Cost of the cervical cancer screening?	1☐	2☐	3☐	4☐
e. Lack of awareness of the need for cervical cancer screening?	1☐	2☐	3☐	4☐
f. Lack of knowledge about what cervical cancer screening involves?	1☐	2☐	3☐	4☐
g. Religious beliefs?	1☐	2☐	3☐	4☐
h. The time it takes to complete the procedure?	1☐	2☐	3☐	4☐
i. The time it takes to receive treatment, if the test indicates it is needed?	1☐	2☐	3☐	4☐
j. Husband's reluctance for his wife to be screened?	1☐	2☐	3☐	4☐

Survey about Perceptions regarding vaginal examinations
→ Survey to be completed **AFTER** introduction of the Expander

Clinician Post examination Questionnaire

Date: ________________
Site: _________________

**1. Please compare the** ***Inserter*** **to the device you usually use. Compared to your usual device,**	**Worse**	**Same**	**Better**
a. Ease of **using the** ***Inserter*** **was**	1☐	2☐	3☐
b. **Length of time needed to perform the procedure was**	1☐	2☐	3☐
c. **The training required for the** ***Inserter*** **was**	1☐	2☐	3☐
d. **The form factor (for translator to know: ergonomics, how it feels to hold) of the device of the** ***Inserter*** **was**	1☐	2☐	3☐
e. **The size of the** ***Inserter*** **was**	1☐	2☐	3☐
f. **The clinical workflow while using the** ***Inserter*** **was**	1☐	2☐	3☐
g. **Overall, compared to your usual device, the** ***Inserter*** **was**	1☐	2☐	3☐

**2. How difficult or easy did you find each of the following?** How difficult or easy was it to…	**Very Difficult**	**Somewhat Difficult**	**Somewhat easy**	**Very easy**	**I didn't do this**
a. **insert the** ***Inserter*****?**	1☐	2☐	3☐	4☐	5☐
b. **expand the vaginal walls with the** ***Inserter*****?**	1☐	2☐	3☐	4☐	5☐
c. **manipulate the** ***Inserter*** **to find the cervix?**	1☐	2☐	3☐	4☐	5☐
d. **remove the** ***Inserter*** **from the vagina?**	1☐	2☐	3☐	4☐	5☐
e. **capture images with the** ***Inserter*** **and** POC*ke*T Colposcope	1☐	2☐	3☐	4☐	5☐
f. **clean the** ***Inserter*****?**	1☐	2☐	3☐	4☐	5☐
g. **sterilize the** ***Inserter*****?**	1☐	2☐	3☐	4☐	5☐

3. How confident are you in your ability to operate the Inserter?
☐1Not at all confident ☐2Slightly confident ☐3Moderately confident ☐4Very confident ☐5Extremely confident

4. Would you use the Inserter in your practice routinely/recommend it to patients?
☐1 YES ☐2 NO
Please explain:
____
____

5. Would you like your hospital to purchase an Inserter?
☐1 YES ☐2 NO
Please explain:
____
____

6. Do you have any additional comments or feedback on the Inserter?
____
____
